# Evaluation of 3,3′-Triazolyl Biisoquinoline *N*,*N*′-Dioxide Catalysts for Asymmetric Hydrosilylation of Hydrazones with Trichlorosilane

**DOI:** 10.3390/catal11091103

**Published:** 2021-09-14

**Authors:** Shiyu Sun, Changgong Xu, Jamielyn Jarvis, Phillip Nader, Brandon Naumann, Abigail Soliven, Roberto Peverati, Norito Takenaka

**Affiliations:** 1 Chemistry Program, Department of Biomedical and Chemical Engineering and Sciences, Florida Institute of Technology, 150 West University Boulevard, Melbourne, FL 32901, USA; 2 Department of Chemistry, Purdue University, West Lafayette, IN 47907, USA

**Keywords:** Lewis base catalysis, trichlorosilane, hydrosilylation, hydrazone, computational chemistry

## Abstract

A new class of axial-chiral biisoquinoline *N*,*N*′-dioxides was evaluated as catalysts for the enantioselective hydrosilylation of acyl hydrazones with trichlorosilane. While these catalysts provided poor to moderate reactivity and enantioselectivity, this study represents the first example of the organocatalytic asymmetric reduction of acyl hydrazones. In addition, the structures and energies of two possible diastereomeric catalyst–trichlorosilane complexes (**2a**–HSiCl_3_) were analyzed using density functional theory calculations.

## 1. Introduction

**Scheme 1.**
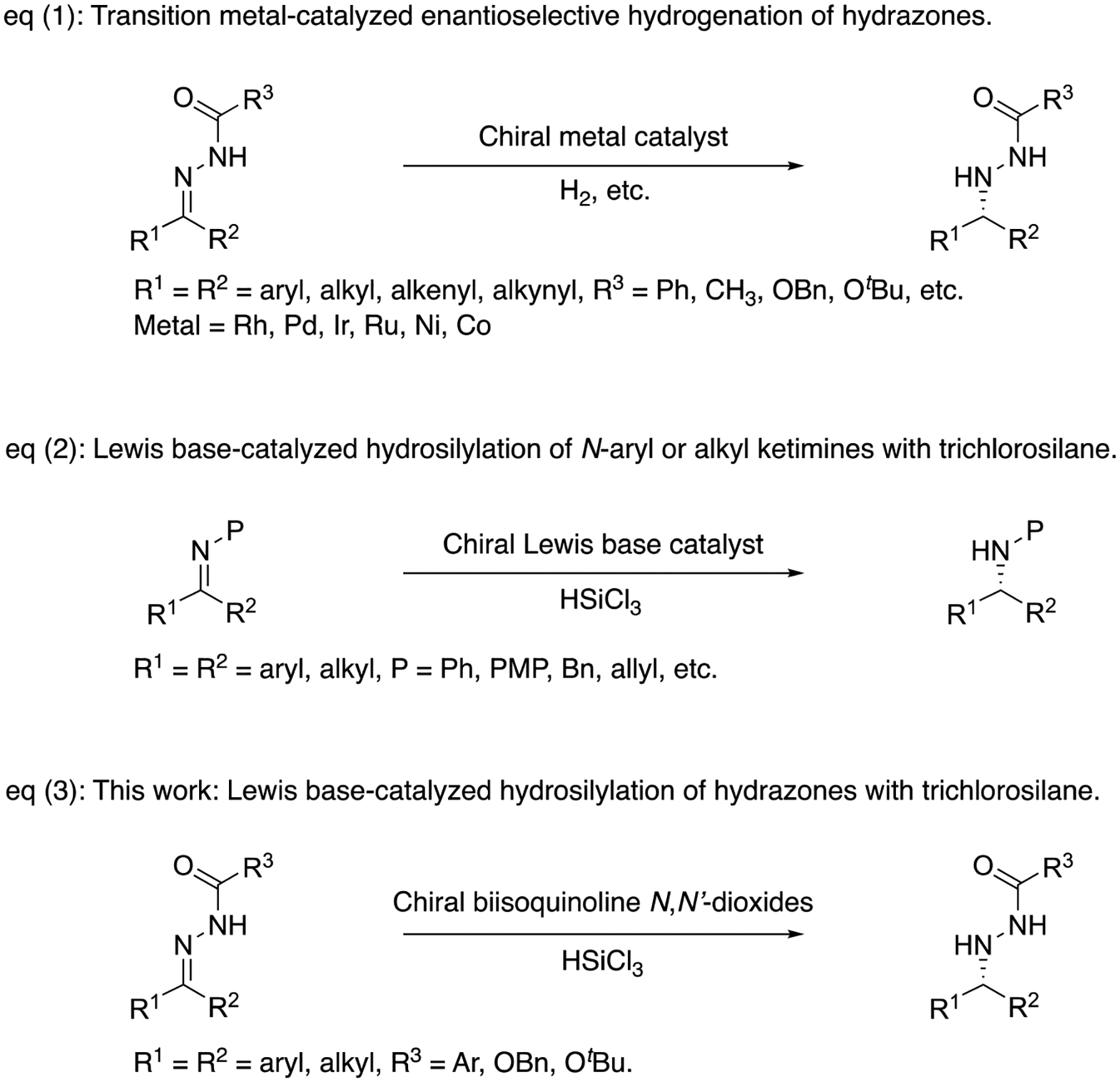
Catalytic asymmetric reduction of C=N bonds.

The chiral hydrazine is an important structural motif found in pharmaceuticals, agrochemicals, natural products, synthetic chiral catalysts, etc. and the catalytic asymmetric reduction of readily available, bench-stable acyl hydrazones provides direct access to such chiral hydrazine derivatives in an enantio-enriched form [1–24]. In 1992, Burk and Feaster reported the first example of the catalytic enantioselective hydrogenation of acyl hydrazones by employing a rhodium/DuPhos catalyst [13]. Since this initial breakthrough, many excellent transition metal-catalyzed methods for the asymmetric reduction of hydrazones have been developed. The majority of these examples utilized rhodium-based catalysts [3–13] but palladium- [14–17], iridium- [18], ruthenium- [19], nickel- [20–23] and cobalt- [24] based catalysts are also reported (Scheme 1, Equation (1)). While their reaction scopes are impressive, no metal-free ‘green’ counterpart (i.e., organocatalysis method) has been reported to the best of our knowledge, except for two scattered examples. There is a patent literature that described one example of the asymmetric hydrosilylation of a tosylhydrazone with trichlorosilane catalyzed by a chiral *N*-formylpyrrolidine (4-methylbenzensulfonic acid 2-(1-phenylethylidene)hydrazide was reduced to the corresponding hydrazine in 94% yield with 36% ee) [25]. The other example is reported by Wang and Sun [26]. Here, it was the trichlorosilane-mediated reductive amination of acetophenone with phenylhydrazine catalyzed by a chiral bis-sulfinamide, which afforded the corresponding 1,1-disubstituted hydrazine in 93% yield with 74% ee.

Among the reported reducing agents that are amenable for organocatalysis (selected reviews; [27–29]), trichlorosilane is particularly attractive because it is a readily available, inexpensive and easy-to-handle liquid (selected reviews; [30–32]). Furthermore, it only produces innoxious NaCl and SiO_2_ as by-products upon quenching with aqueous NaOH or NaHCO_3_ solutions, which are easily separable from the reaction products (i.e., organic compounds). Trichlorosilane reversibly forms a hypervalent silicon complex with Lewis-bases that is believed to be the active reducing species. Since Matsumura’s seminal work, that employed *N*-formylproline-derived catalysts [33,34], and the milestone achieved by Malkov and Kočovský with their *N*-methyl-_L_-valine-based catalysts [35–37], numerous chiral Lewis-base catalysts have been reported for the asymmetric hydrosilylation of ketimines with trichlorosilane (selected reviews; [30–32], selected references; [38–45]). While the majority of these catalysts are amide-based Lewis-bases, other kinds of Lewis-bases are also reported, which includes pyridine *N*-oxides (selected references; [46–51]), phosphine oxides (selected references; [52–54]), and sulfinamides (selected references; [55,56]). Despite a plethora of reports in this area, the Lewis-base-catalyzed trichlorosilane-mediated reduction of ketimines currently remains limited to *N*-aryl and alkyl protected ones (Scheme 1, Equation (2)). The lack of acyl hydrazones as substrates for this method is presumably because their N–C=O unit could possibly bind trichlorosilane competitively with amide-based catalysts to produce racemic products. As a matter of fact, the only examples of the hydrazones that were enantioselectively reduced with chiral Lewis-bases and trichlorosilane are *N*-tosyl- and *N*-phenylhydrazones as mentioned above [25,26]. In this context, we became interested in evaluating axial-chiral 3,3′-triazolyl biisoquinoline *N*,*N*′-dioxide catalysts [57] for the hydrosilylation of acyl hydrazones with trichlorosilane (Scheme 1, Equation (3)). Herein describes our preliminary investigations in this area.

## 2. Results and Discussion

**Scheme 2.**
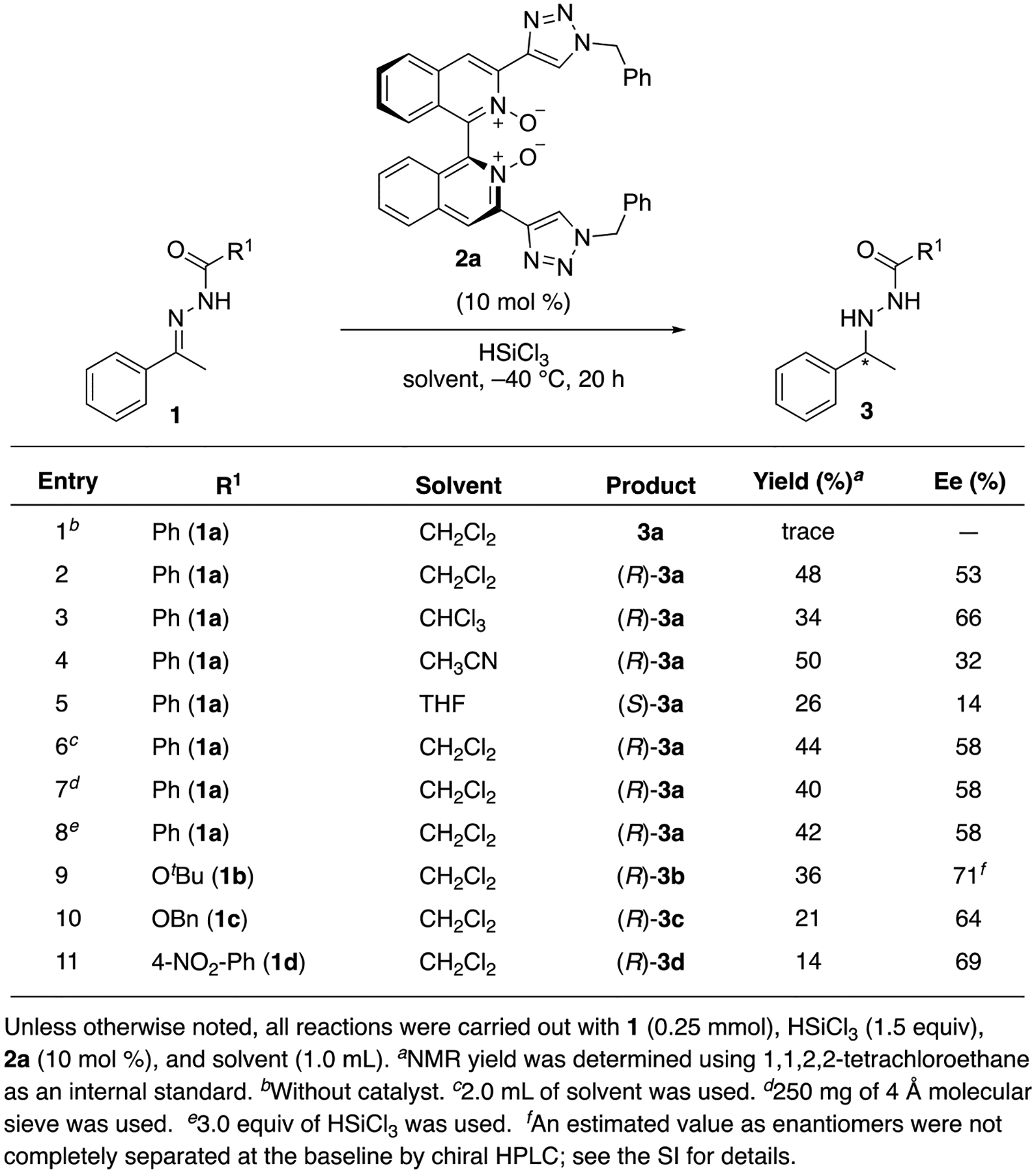
Evaluation of the reaction parameters.

We recently developed the modular method to synthesize axial-chiral 3,3′-triazolyl biisoquinoline *N*,*N*′-dioxides from readily available triazoles and optically pure 3,3′-dibromo-biisoquinoline *N*,*N*′-dioxide [57] as part of our longstanding interests in developing new chiral Lewis-bases [58–62]. Since this new class of catalysts was found capable of activating trichlorosilane at relatively low temperatures, we envisioned that they might be able to catalyze the reduction of acyl hydrazones under conditions where no background reaction would take place. We set out on our investigation by employing benzoyl hydrazone **1a** as a model substrate (Scheme 2). To our delight, the background reaction was found negligible at −40 °C, and catalyst **2a** provided hydrazine (*R*)-**3a** in 48% yield with 53% ee (entries 1 and 2). Next, we looked at several solvents that are commonly used for trichlorosilane-mediated reactions. Chloroform provided the product with a lower yield but with a slightly higher enantioselectivity (34% yield, 66% ee). Acetonitrile gave **3a** in a comparable yield but with a lower ee of 32%. The reaction in tetrahydrofuran afforded the opposite enantiomer (*S*)-**3a** with a much lower yield and selectivity (entry 5). Overall, dichloromethane was found optimum. We tested with twice as much solvent since benzoyl hydrazone **1a** was not fully dissolved under the reaction conditions (entry 6). However, it did not improve the result. Previously, we found that 4 Å molecular sieve was an effective acid scavenger for adventitious HCl in trichlorosilane [63], but its use did not positively impact the outcome in the present case (entry 7). The use of 3.0 equivalent of trichlorosilane did not improve the yield, either (entry 8). As the protecting groups on hydrazones are known to influence their reactivities and enantioselectivities in many cases (e.g., see; [19]), we evaluated the Boc and Cbz protected hydrazones (**1b** and **1c** in entries 9 and 10, respectively). While enantiomeric excesses of the corresponding products were slightly higher than that of the benzoyl counterpart, both Boc and Cbz protecting groups adversely affected the yields. Since the C=O unit of Boc or Cbz group is more Lewis basic than that of the benzoyl counterpart, we tested a less Lewis basic hydrazone (**1d**). However, the yield decreased to 14% albeit with a slightly higher enantioselectivity (entry 11). Overall, the present method was found to be quite sensitive to reaction solvents and the hydrazone protecting groups.

**Scheme 3.**
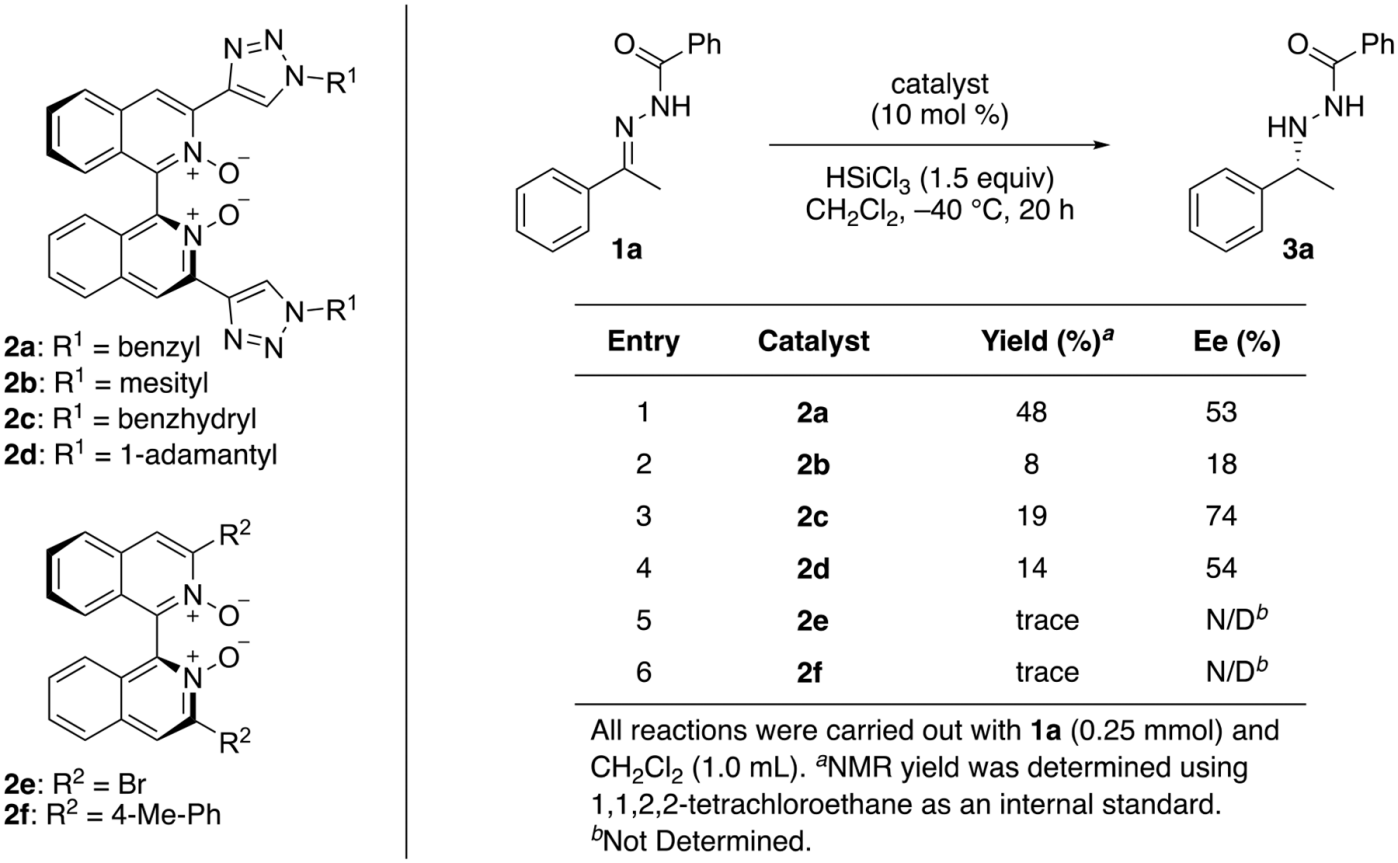
Evaluation of 3,3′-substituents of axial-chiral biisoquinoline catalysts.

Next, we evaluated different triazolyl groups on the biisoquinoline that are expected to play important roles on the catalyst’s reactivity and selectivity (Scheme 3, entries 1–4). Catalyst **2a** was clearly superior to the other three catalysts (**2b**–**d**) in terms of their reactivity. Catalyst **2c** was more enantioselective than others, albeit with a low yield. These results indicate that the reactivity and selectivity of this new class of catalysts can be tuned by changing the triazolyl groups. We also compared these triazolyl catalysts to conventional 3,3′-substituted biisoquinoline *N*,*N*′-dioxides (entries 5 and 6). To our surprise, neither **2e** nor **2f** promoted the reaction although **2f** was as reactive as **2b**–**d** for the hydrosilylation of an *N*-phenyl ketimine with trichlorosilane [57]. Nonetheless, these results clearly demonstrated that this new class of axial-chiral biisoquinolines is indeed complementary to the existing Lewis-base catalysts and bode well for the development of their applications.

**Scheme 4.**
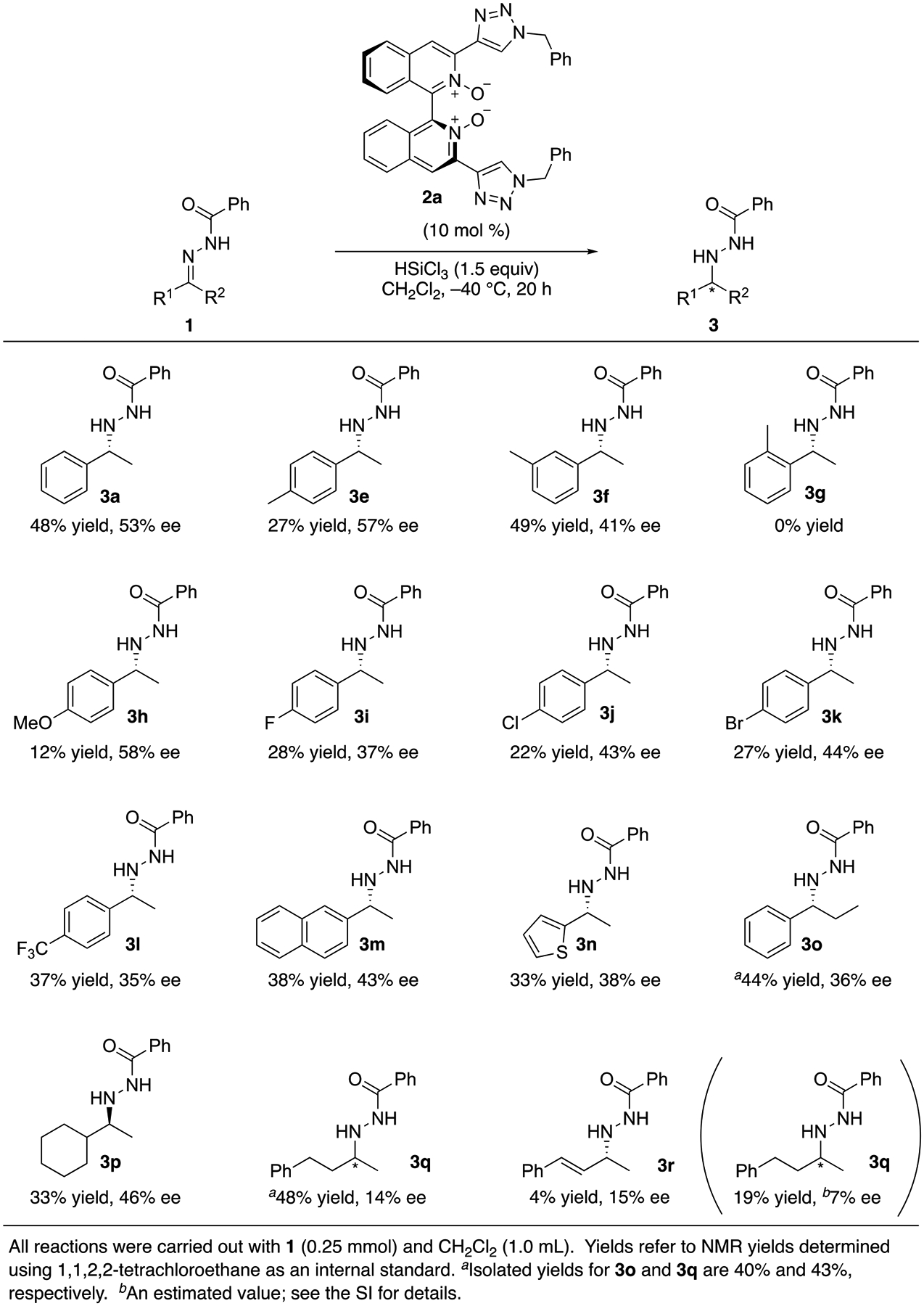
Evaluation of the extent to which **2a** catalyzed the reduction.

As we determined the basic reaction parameters, we proceeded to evaluate the extent to which the present catalytic system could enantioselectively promote the hydrosilylation of various benzoyl hydrazones with trichlorosilane (Scheme 4). To our surprise, a paramethyl substitution (**3e**)—which is a minimal change from the model substrate (**3a**)—had a detrimental effect on the chemical yield while the corresponding meta-substitution (**3f**) did not. An ortho-methyl substitution (that is known to push the aromatic ring out of conjugation with a C=N bond) completely shut down the reaction (**3g**). Eventually, it was gleaned that the para-substitutions have adverse effects on the reactivity but not much on the enantioselectivity regardless of their electronic nature (**3e**, **3h**–**m**) (these enantioselectivities are approximately the same). A heteroaromatic hydrazone was moderately less reactive and selective than the model substrate (**3n**). Although an ethyl group at the C=N bond is in general expected to lead to an increased steric demand in the TS, it did not affect the reactivity in the present case (**3o**). It is noteworthy that a cyclohexyl counterpart provided the opposite sense of enantioselection to the model substrate (**3p**). Differentiation of the two similar alkyl groups franking the C=N bond was difficult by the present catalytic system (**3q**). Unreacted hydrazones and corresponding ketones were the major components of the crude reaction mixtures besides the desired products, and no significant amounts of by-products were observed for **1a**–**1q**. An α,β-unsaturated hydrazone was not a viable substrate for this method as the conjugate reduction took place (**3r**) [64].

**Scheme 5.**
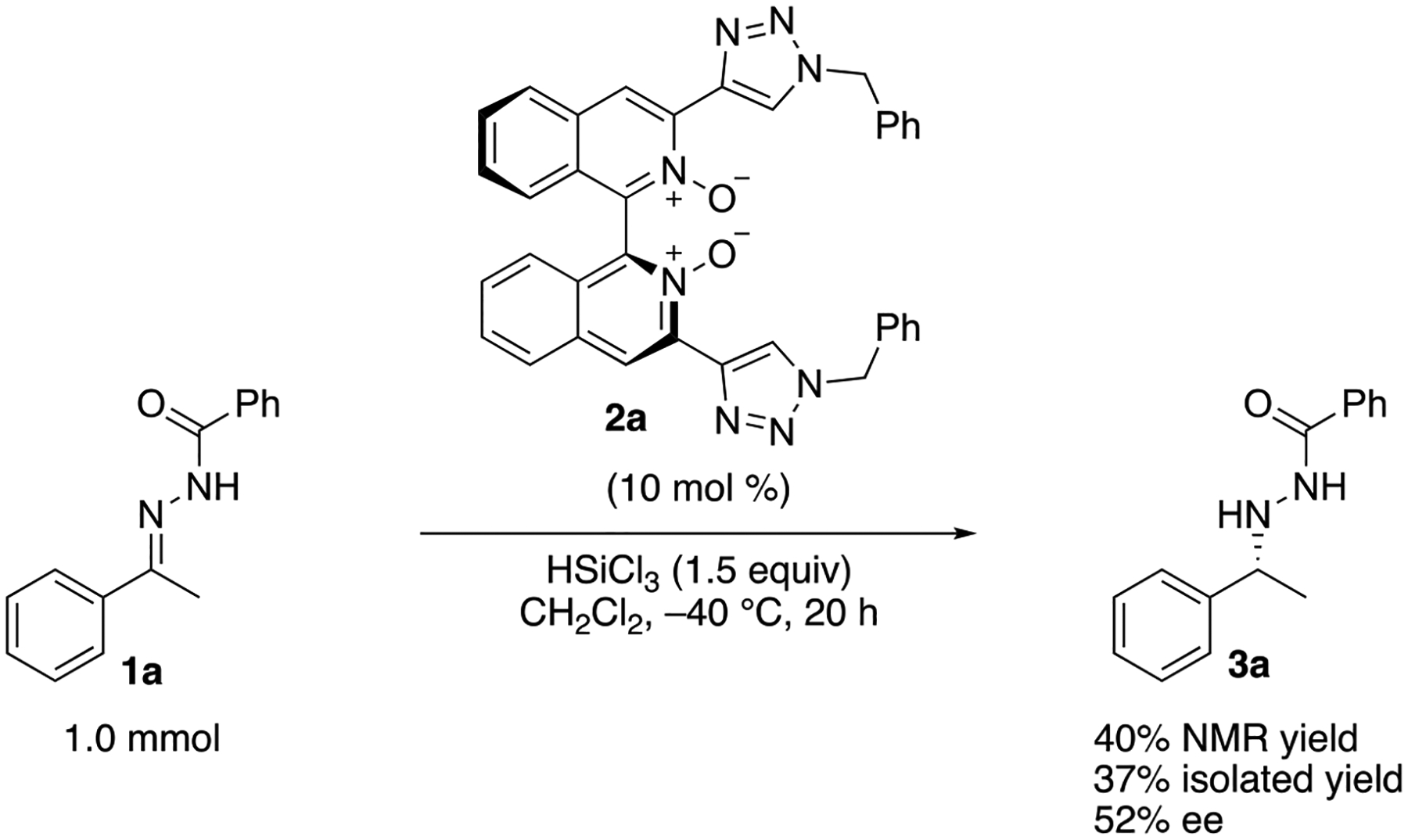
The 1.0 mmol scale reaction.

We also tested a 1.0 mmol scale reaction with the model substrate. To our delight, it provided essentially the same result (Scheme 5), demonstrating a potential robustness of the method. Furthermore, catalyst **2a** was quantitatively recovered after a flash column chromatography on silica gel (see Supplementary Materials for details). The recovered catalyst promoted the model reaction (**1a** on 0.25 mmol scale) with no loss in reactivity and enantioselectivity.

**Figure 1.**
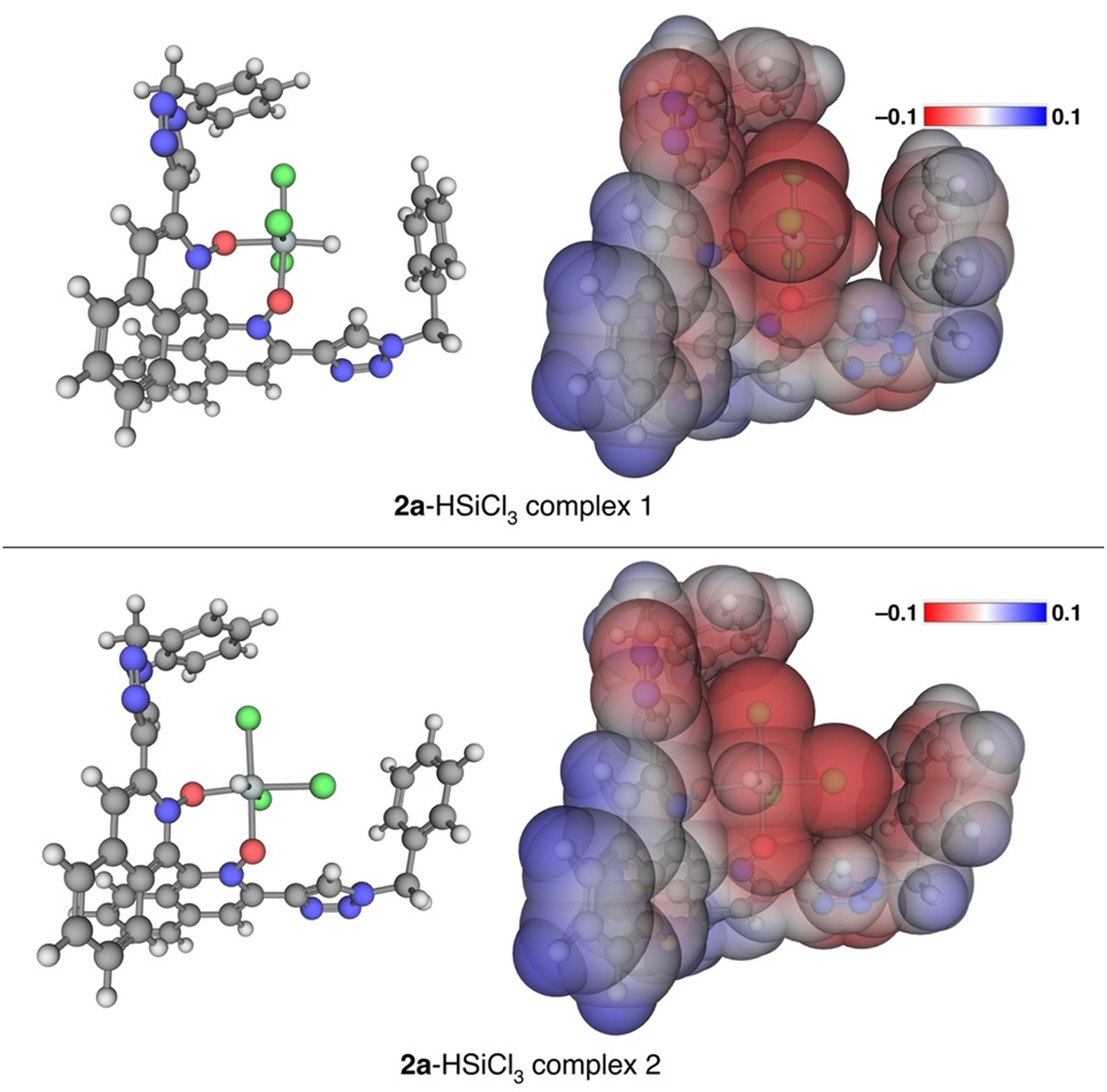
Computed structures of the two lowest energy minima for the **2a**-HSiCl_3_ complex (i.e., two diastereomeric complexes) calculated with PBEh-3c//C-PCM (DCM). Both are shown with balls-and-sticks (**left**) and space filling (**right**) models. Molecular electrostatic potentials are also shown in the space filling models. Complex 1 (**top**) is 1.91 kcal/mol lower in energy than complex 2 (**bottom**).

The structure of the active reducing species generated from a chiral catalyst and HSiCl_3_ is considered to play a central role for the enantioselectivity of a reaction. Even with notable advances made in this area (selected references; [35–45]), it remains largely elusive and significantly challenging to control the relative populations and reactivities of diastereomeric reducing species that are reversibly produced from a chiral Lewis-base and trichlorosilane. *C*_2_-symmetric **2a** and HSiCl_3_ can give rise to two diastereomeric complexes that are expected to have different enantioselectivities (as long as **2a** acts as a bidentate Lewis-base). Therefore, the binding geometry of **2a** to HSiCl_3_ was investigated computationally with the aim of shedding some light on the structure of the active reducing species. To our delight, **2a** was found to bind to HSiCl_3_ through its two oxygen atoms (i.e., a *C*_2_-symmetric bidentate ligand), generating two diastereomeric complexes (Figure 1). Complex 1 was found to be 1.91 kcal/mol lower in energy than the complex 2. The analysis of their electrostatic potentials revealed an anion–π-type interaction between the hydrogen atom in complex 1 or one of the chlorine atoms in complex 2 and the phenyl ring. It should be mentioned that a pileup of electron density occurs at the peripheral atoms of a hypervalent silicon complex of this kind [59,65,66]. This anion–π-type interaction appears to effectively lock the conformation of the benzyl group at least at the ground state, leading to a well-defined chiral pocket around the hypervalent silicon atom. This computationally identified non-covalent attractive interaction could offer a possible basis to rationalize why **2a** (benzyl) was as enantioselective as **2d** (1-adamantyl), and why **2c** (benzhydryl) was substantially more enantioselective than **2d** (53% ee, 54% ee and 74% ee, respectively; Scheme 3).

## 3. Conclusions

Axial-chiral 3,3′-triazolyl biisoquinoline *N*,*N*′-dioxides offer potential for functioning as effective catalysts for the asymmetric hydrosilylation of acyl hydrazones with trichlorosilane. Since catalyst’s triazolyl units indeed tuned the reactivity and enantioselectivity of the reaction and our modular synthesis allows ready access to a variety of 3,3′-triazolyl biisoquinoline *N*,*N*′-dioxides, potential for the identification of more effective catalysts than those presented herein clearly exits.

## Supplementary Material

SI_Takenaka_2021_Evaluation of 3,3'-triazolyl biisoquinoline N,N'-dioxide Catalysts for Asymmetric Hydrosilylation of Hydrazones with trichlorosilane

## Data Availability

Data is contained within the article and Supporting Information.

## References

[1] Abdine RAA; Hedouin G; Colobert F; Wencel-Delord J Metal-Catalyzed Asymmetric Hydrogenation of C=N Bonds. ACS Catal 2021, 11, 215–247.

[2] Xie J-H; Zhu S-F; Zhou Q-L Transition Metal-Catalyzed Enantioselective Hydrogenation of Enamines and Imines. Chem. Rev 2011, 111, 1713–1760. 21166392 10.1021/cr100218m

[3] Fan D; Hu Y; Jiang F; Zhang Z; Zhang W Rhodium-Catalyzed Chemo- and Enantioselective Hydrogenation of Alkynyl-Aryl Hydrazones. Adv. Synth. Catal 2018, 360, 2228–2232.

[4] Hu Q; Hu Y; Liu Y; Zhang Z; Liu Y; Zhang W Rh-Catalyzed Chemo- and Enantioselective Hydrogenation of Allylic Hydrazones. Chem. A Eur. J 2017, 23, 1040–1043. 10.1002/chem.201605579 27905139

[5] Haddad N; Qu B; Rodriguez S; van der Veen L; Reeves DC; Gonnella NC; Lee H; Grinberg N; Ma S; Krishnamurthy D; Catalytic asymmetric hydrogenation of heterocyclic ketone-derived hydrazones, pronounced solvent effect on the inversion of configuration. Tetrahedron Lett 2011, 52, 3718–3722.

[6] Yoshikawa N; Tan L; McWilliams JC; Ramasamy D; Sheppard R Catalytic Enantioselective Hydrogenation of N-Alkoxycarbonyl Hydrazones: A Practical Synthesis of Chiral Hydrazines. Org. Lett 2010, 12, 276–279. 20017501 10.1021/ol902602c

[7] Gavryushin A; Polborn K; Knochel P Novel chiral diphosphine ligands with a pinene core obtained via an allylphosphinite–allylphosphine oxide rearrangement. Tetrahedron Asymmetry 2004, 15, 2279–2288.

[8] Tappe K; Knochel P New efficient synthesis of Taniaphos ligands: Application in ruthenium- and rhodium-catalyzed enantioselective hydrogenations. Tetrahedron Asymmetry 2004, 15, 91–102.

[9] Ireland T; Tappe K; Grossheimann G; Knochel P Synthesis of a New Class of Chiral 1,5-Diphosphanylferrocene Ligands and Their Use in Enantioselective Hydrogenation. Chem. A Eur. J 2002, 8, 843–852. 10.1002/1521-3765(20020215)8:4<843::aid-chem843>3.0.co;2-9 11857698

[10] Yamazaki A; Achiwa I; Horikawa K; Tsurubo M; Achiwa K Preparation and Application of a Novel Type of Chiral Biphosphine Ligand (NORPHOS-7X) Containing a Hetero-Functional Group. Synlett 1997, 1997, 455–456.

[11] Burk MJ; Martinez JP; Feaster JE; Cosford N Catalytic Asymmetric Reductive Amination of Ketones via Highly Enantioselective Hydrogenation of the C=N Double Bond. Tetrahedron 1994, 50, 4399–4428.

[12] Burk MJ; Gross MF New Chiral 1,1′-Bis(phospholano)ferrocene Ligands for Asymmetric Catalysis. Tetrahedron Lett 1994, 35, 9363–9366.

[13] Burk MJ; Feaster JE Enantioselective Hydrogenation of the C=N Group: A Catalytic Asymmetric Reductive Animation Procedure. J. Am. Chem. Soc 1992, 114, 6266–6267.

[14] Wang Y-Q; Lu S-M; Zhou Y-G Highly Enantioselective Pd-Catalyzed Asymmetric Hydrogenation of Activated Imines. J. Org. Chem 2007, 72, 3729–3734. 17444684 10.1021/jo0700878

[15] Chen Z-P; Hu S-B; Chen M-W; Zhou Y-G Synthesis of Chiral Fluorinated Hydrazines via Pd-Catalyzed Asymmetric Hydrogenation. Org. Lett 2016, 18, 2676–2679. 27196122 10.1021/acs.orglett.6b01118

[16] Chen Z-P; Hu S-B; Zhou J; Zhou Y-G Synthesis of Chiral Trifluoromethyl-Substituted Hydrazines via Pd-Catalyzed Asymmetric Hydrogenation and Reductive Amination. ACS Catal 2015, 5, 6086–6089.

[17] Chen Z-P; Chen M-W; Shi L; Yu C-B; Zhou Y-G Pd-catalyzed asymmetric hydrogenation of fluorinated aromatic pyrazol-5-ols via capture of active tautomers. Chem. Sci 2015, 6, 3415–3419. 29511507 10.1039/c5sc00835b PMC5659069

[18] Chang M; Liu S; Huang K; Zhang X Direct Catalytic Asymmetric Reductive Amination of Simple Aromatic Ketones. Org. Lett 2013, 15, 4354–4357. 23937659 10.1021/ol401851c

[19] Schuster CH; Dropinski JF; Shevlin M; Li H; Chen S Ruthenium-Catalyzed Enantioselective Hydrogenation of Hydrazones. Org. Lett 2020, 22, 7562–7566. 32946691 10.1021/acs.orglett.0c02756

[20] Li B; Liu D; Hu Y; Chen J; Zhang Z; Zhang W Nickel-Catalyzed Asymmetric Hydrogenation of Hydrazones. Eur. J. Org. Chem 2021, 2021, 3421–3425.

[21] Yang P; Zhang C; Ma Y; Zhang C; Li A; Tang B; Zhou JS Nickel-Catalyzed N-Alkylation of Acylhydrazines and Arylamines Using Alcohols and Enantioselective Examples. Angew. Chem. Int. Ed 2017, 56, 14702–14706. 10.1002/anie.201708949 28960690

[22] Yang P; Lim LH; Chuanprasit P; Hirao H; Zhou JS Nickel-Catalyzed Enantioselective Reductive Amination of Ketones with Both Arylamines and Benzhydrazide. Angew. Chem. Int. Ed 2016, 55, 12083–12087. 10.1002/anie.201606821 27572720

[23] Xu H; Yang P; Chuanprasit P; Hirao H; Zhou JS Nickel-Catalyzed Asymmetric Transfer Hydrogenation of Hydrazones and Other Ketimines. Angew. Chem. Int. Ed 2015, 54, 5112–5116. 10.1002/anie.201501018 25737093

[24] Hu Y; Zhang Z; Zhang J; Liu Y; Gridnev ID; Zhang W Cobalt-Catalyzed Asymmetric Hydrogenation of C=N Bonds Enabled by Assisted Coordination and Nonbonding Interactions. Angew. Chem. Int. Ed 2019, 58, 15767–15771. 10.1002/anie.201909928 31464078

[25] Iwasaki F; Matsumura K; Onomura O Jpn. Kokai Tokkyo Koho (2001). Patent JP 2001270851A, 2 October 2001.

[26] Wang T; Di X; Wang C; Zhou L; Sun J Reductive Hydrazination with Trichlorosilane: A Method for the Preparation of 1,1-Disubstituted Hydrazines. Org. Lett 2016, 18, 1900–1903. 27049166 10.1021/acs.orglett.6b00675

[27] Chen W; Tan C; Wang H; Ye X The Development of Organocatalytic Asymmetric Reduction of Carbonyls and Imines Using Silicon Hydrides. Eur. J. Org. Chem 2021, 2021, 3091–3112.

[28] Faísca Phillips AM; Pombeiro AJL Recent advances in organocatalytic enantioselective transfer hydrogenation. Org. Biomol. Chem 2017, 15, 2307–2340. 28229149 10.1039/c7ob00113d

[29] Rossi S; Benaglia M; Massolo E; Raimondi L Organocatalytic strategies for enantioselective metal-free reductions. Catal. Sci. Technol 2014, 4, 2708.

[30] Kočovský P; Malkov AV Lewis Bases as Catalysts in the Reduction of Imines and Ketones with Silanes (n→σ*). In Lewis Base Catalysis in Organic Synthesis; Wiley-VCH Verlag GmbH & Co. KGaA: Weinheim, Germany, 2016; Volume 3, pp. 1077–1112. ISBN 9783527675142.

[31] Jones S; Warner CJA Trichlorosilane mediated asymmetric reductions of the C=N bond. Org. Biomol. Chem 2012, 10, 2189. 22307888 10.1039/c2ob06854k

[32] Guizzetti S; Benaglia M Trichlorosilane-Mediated Stereoselective Reduction of C=N Bonds. Eur. J. Org. Chem 2010, 2010, 5529–5541.

[33] Onomura O; Kouchi Y; Iwasaki F; Matsumura Y New organic activators for the enantioselective reduction of aromatic imines with trichlorosilane. Tetrahedron Lett 2006, 47, 3751–3754. 10.1021/ol0613822 16898818

[34] Iwasaki F; Onomura O; Mishima K; Kanematsu T; Maki T; Matsumura Y First chemo- and stereoselective reduction of imines using trichlorosilane activated with N-formylpyrrolidine derivatives. Tetrahedron Lett 2001, 42, 2525–2527.

[35] Malkov AV; Vranková K; Stončius S; Kočovský P Asymmetric Reduction of Imines with Trichlorosilane, Catalyzed by Sigamide, an Amino Acid-Derived Formamide: Scope and Limitations. J. Org. Chem 2009, 74, 5839–5849. 19530670 10.1021/jo900561h

[36] Malkov AV; Stončius S; MacDougall KN; Mariani A; McGeoch GD; Kočovský P Formamides derived from N-methyl amino acids serve as new chiral organocatalysts in the enantioselective reduction of aromatic ketimines with trichlorosilane. Tetrahedron 2006, 62, 264–284.

[37] Malkov AV; Mariani A; MacDougall KN; Kočovský P Role of Noncovalent Interactions in the Enantioselective Reduction of Aromatic Ketimines with Trichlorosilane. Org. Lett 2004, 6, 2253–2256. 15200333 10.1021/ol049213+

[38] Genoni A; Benaglia M; Mattiolo E; Rossi S; Raimondi L; Barrulas PC; Burke AJ Synthesis of an advanced precursor of Rivastigmine: Cinchona -derived quaternary ammonium salts as organocatalysts for stereoselective imine reductions. Tetrahedron Lett 2015, 56, 5752–5756.

[39] Barrulas PC; Genoni A; Benaglia M; Burke AJ Cinchona-Derived Picolinamides: Effective Organocatalysts for Stereoselective Imine Hydrosilylation. Eur. J. Org. Chem 2014, 2014, 7339–7342.

[40] Hu X-Y; Zhang M-M; Shu C; Zhang Y-H; Liao L-H; Yuan W-C; Zhang X-M Enantioselective Lewis-Base-Catalyzed Asymmetric Hydrosilylation of Substituted Benzophenone N-Aryl Imines: Efficient Synthesis of Chiral (Diarylmethyl)amines. Adv. Synth. Catal 2014, 356, 3539–3544.

[41] Zhang Z; Rooshenas P; Hausmann H; Schreiner P Asymmetric Transfer Hydrogenation of Ketimines with Trichlorosilane: Structural Studies. Synthesis 2009, 2009, 1531–1544.

[42] Guizzetti S; Benaglia M; Rossi S Highly Stereoselective Metal-Free Catalytic Reduction of Imines: An Easy Entry to Enantiomerically Pure Amines and Natural and Unnatural α-Amino Esters. Org. Lett 2009, 11, 2928–2931. 19480447 10.1021/ol900945h

[43] Wang C; Wu X; Zhou L; Sun J A highly enantioselective organocatalytic method for reduction of aromatic N-alkyl ketimines. Chem. A Eur. J 2008, 14, 8789–8792. 10.1002/chem.200801479 18752235

[44] Zheng H; Deng J; Lin W; Zhang X Enantioselective hydrosilylation of ketimines with trichlorosilane promoted by chiral N-picolinoylaminoalcohols. Tetrahedron Lett 2007, 48, 7934–7937.

[45] Malkov AV; Liddon AJPS; Ramírez-López P; Bendová L; Haigh D; Kočovský P Remote Chiral Induction in the Organocatalytic Hydrosilylation of Aromatic Ketones and Ketimines. Angew. Chem. Int. Ed 2006, 45, 1432–1435. 10.1002/anie.200503941 16425324

[46] Žeimyte S; Stončius S Chiral bipyridine-annulated bicyclo [3.3.1] nonane N-oxide organocatalysts for stereoselective allylation˙ and hydrosilylation reactions. Tetrahedron 2021, 78, 131831.

[47] Wrzeszcz Z; Siedlecka R Heteroaromatic N-Oxides in Asymmetric Catalysis: A Review. Molecules 2020, 25, 330. 10.3390/molecules25020330 PMC7024222 31947566

[48] Dong M; Wang J; Wu S; Zhao Y; Ma Y; Xing Y; Cao F; Li L; Li Z; Zhu H Catalytic Mechanism Study on the 1,2- and 1,4-Transfer Hydrogenation of Ketimines and β-Enamino Esters Catalyzed by Axially Chiral Biscarboline-Based Alcohols. Adv. Synth. Catal 2019, 361, 4602–4610.

[49] Xing Y; Wu S; Dong M; Wang J; Liu L; Zhu H Synthesis and application of axially chiral biscarbolines with functional N-O and sulfone for 1,2-transfer hydrogenations of ketimines. Tetrahedron 2019, 75, 130495.

[50] Pei Y-N; Deng Y; Li J-L; Liu L; Zhu H-J New chiral biscarboline N,N′-dioxide derivatives as catalyst in enantioselective reduction of ketoimines with trichlorosilane. Tetrahedron Lett 2014, 55, 2948–2952.

[51] Pan W; Deng Y; He J-B; Bai B; Zhu H-J Highly efficient asymmetric-axle-supported N–O amides in enantioselective hydrosilylation of ketimines with trichlorosilane. Tetrahedron 2013, 69, 7253–7257.

[52] Warner CJA; Berry SS; Jones S Evaluation of bifunctional chiral phosphine oxide catalysts for the asymmetric hydrosilylation of ketimines. Tetrahedron 2019, 75, 130733.

[53] Warner CJA; Reeder AT; Jones S P-Chiral phosphine oxide catalysed reduction of prochiral ketimines using trichlorosilane. Tetrahedron Asymmetry 2016, 27, 136–141.

[54] Cauteruccio S; Dova D; Benaglia M; Genoni A; Orlandi M; Licandro E Synthesis, Characterisation, and Organocatalytic Activity of Chiral Tetrathiahelicene Diphosphine Oxides. Eur. J. Org. Chem 2014, 2014, 2694–2702.

[55] Pei D; Wang Z; Wei S; Zhang Y; Sun J S-Chiral Sulfinamides as Highly Enantioselective Organocatalysts. Org. Lett 2006, 8, 5913–5915. 17134304 10.1021/ol062633+

[56] Pei D; Zhang Y; Wei S; Wang M; Sun J Rationally-Designed S- Chiral Bissulfinamides as Highly Enantioselective Organocatalysts for Reduction of Ketimines. Adv. Synth. Catal 2008, 350, 619–623.

[57] Sun S; Reep C; Zhang C; Captain B; Peverati R; Takenaka N Design and synthesis of 3,3′-triazolyl biisoquinoline N,N′-dioxides via Hiyama cross-coupling of 4-trimethylsilyl-1,2,3-triazoles. Tetrahedron Lett.. in press. 10.1016/j.tetlet.2021.153338 PMC8673909 34924634

[58] Morgante P; Captain B; Chouinard CD; Peverati R; Takenaka N Synthesis of electrophilic N-heterocyclic carbenes based on azahelicene. Tetrahedron Lett 2020, 61, 152143.

[59] Reep C; Morgante P; Peverati R; Takenaka N Axial-Chiral Biisoquinoline N, N ^0^-Dioxides Bearing Polar Aromatic C-H Bonds as Catalysts in Sakurai-Hosomi-Denmark Allylation. Org. Lett 2018, 20, 5757–5761. 30199262 10.1021/acs.orglett.8b02457

[60] Peng Z; Takenaka N Applications of Helical-Chiral Pyridines as Organocatalysts in Asymmetric Synthesis. Chem. Rec 2013, 13, 28–42. 23172849 10.1002/tcr.201200010

[61] Chen J; Captain B; Takenaka N Helical Chiral 2,2′-Bipyridine N- Monoxides as Catalysts in the Enantioselective Propargylation of Aldehydes with Allenyltrichlorosilane. Org. Lett 2011, 13, 1654–1657. 21366250 10.1021/ol200102c

[62] Takenaka N; Sarangthem RS; Captain B Helical Chiral Pyridine N -Oxides: A New Family of Asymmetric Catalysts. Angew. Chem. Int. Ed 2008, 47, 9708–9710. 10.1002/anie.200803338 18988214

[63] Xu C; Reep C; Jarvis J; Naumann B; Captain B; Takenaka N Asymmetric Catalytic Ketimine Mannich Reactions and Related Transformations. Catalysts 2021, 11, 712. 34745653 10.3390/catal11060712 PMC8570560

[64] Sugiura M; Sato N; Kotani S; Nakajima M Lewis base-catalyzed conjugate reduction and reductive aldol reaction of α,β-unsaturated ketones using trichlorosilane. Chem. Commun 2008, 2, 4309. 10.1039/b807529h 18802553

[65] Lu T; Porterfield MA; Wheeler SE Explaining the Disparate Stereoselectivities of N-Oxide Catalyzed Allylations and Propargylations of Aldehydes. Org. Lett 2012, 14, 5310–5313. 23050736 10.1021/ol302493d

[66] Denmark SE; Beutner GL Lewis Base Catalysis in Organic Synthesis. Angew. Chem. Int. Ed 2008, 47, 1560–1638. 10.1002/anie.200604943 18236505

