# Supplementary material for: Evaluation of 3,3′-Triazolyl Biisoquinoline N,N′-Dioxide Catalysts for Asymmetric Hydrosilylation of Hydrazones with Trichlorosilane
Source: Catalysts. Author manuscript; Available in PMC 2022 Oct 24. (PMC9589403; doi:10.3390/catal11091103)
Supplement: SI_Takenaka_2021_Evaluation of 3,3'-triazolyl biisoquinoline N,N'-dioxide Catalysts for Asymmetric Hydrosilylation of Hydrazones with trichlorosilane [file NIHMS1808197-supplement-SI.pdf]

Evaluation of 3,3'-Triazolyl Biisoquinoline N,N'-dioxide Catalysts for Asymmetric Transfer Hydrogenation of Hydrazones with Trichlorosilane

Shiyu Sun[1], Changgong Xu[1], Jamielyn Jarvis[1], Phillip Nader[1], Brandon Naumann[1], Abigail Soliven[2], Roberto Peverati[1,*] and Norito Takenaka[1,*]

[1]Chemistry Program, Department of Biomedical and Chemical Engineering and Sciences,
Florida Institute of Technology, 150 West University Boulevard,
Melbourne, Florida 32901-6975, United States
rpeverati@fit.edu, ntakenaka@fit.edu

[2]Department of Chemistry, Purdue University, West Lafayette, Indiana 47907, United States

Supporting Information

Table of Contents

1. General Information S2

2. Experimental Procedures S3

3. Computational Procedures S18

4. Reference S19

5. ^{1}H, ^{13}C NMR and HPLC Spectra S21

1. General Information.

All reactions were carried out in oven- or flame-dried glassware under an atmosphere of dry argon or nitrogen unless otherwise noted. Except as otherwise indicated, all reactions were magnetically stirred and monitored by analytical thin-layer chromatography using SiliCycle® Inc. and EMD Millipore pre-coated silica gel plates with F_{254} indicator. Visualization was accomplished by UV light (254 nm) with combination of potassium permanganate. Flash column chromatography was performed according to the method of Still [1] using silica gel 60 (mesh 230-400) supplied by SiliCycle® Inc. Yields refer to chromatographically and spectroscopically pure compounds, unless otherwise stated.

Commercial grade reagents and solvents were purchased from Sigma-Aldrich, Alfa-Aesar, Acros, Fisher, TCI, and VWR, and were used as received without further purification except as indicated below. Trichlorosilane was distilled over calcium hydride under an atmosphere of dry nitrogen prior to use. Dichloromethane, chloroform, and acetonitrile were freshly distilled over calcium hydride under an atmosphere of dry nitrogen prior to use. Tetrahydrofuran was freshly distilled over sodium and benzophenone under an atmosphere of dry nitrogen prior to use.

All racemic hydrazine samples were prepared by sodium cyanoborohydride reduction of corresponding hydrazones.

All ^{1}H NMR and ^{13}C NMR spectra were obtained using a Bruker 400 Ultrashield or an Oxford AS400 Spectrometer (^{1}H 400 MHz, ^{13}C 100 MHz) at ambient temperature in $CDCl_3$ purchased from Cambridge Isotope Laboratories, Inc. Chemical shifts in ^{1}H NMR spectra are reported in parts per million (ppm) respective to tetramethylsilane (δ

0.00 ppm) unless otherwise noted. The proton spectra are reported as follows δ (multiplicity, coupling constant *J*, number of protons). Multiplicities are indicated by s (singlet), d (doublet), t (triplet), q (quartet), m (multiplet), and br (broad). Chemical shifts in ^{13}C NMR spectra are reported in ppm respective to $CDCl_3$ (δ 77.0 ppm). All ^{13}C NMR spectra were recorded with complete proton decoupling. Infrared (IR) spectra were recorded using a Nicolet iS5 FT-IR instrument. MS data were obtained using an Agilent 6100 Quadrupole LC/MS. HRMS data were obtained at USF Mass Spec and Peptide Core Facility in Department of Chemistry at University of South Florida. Optical rotations were measured using a Jasco P2000 Polarimeter at 589 nm and were reported as $[\alpha]_D^{T\,°C}$, where C is reported in g/mL.

2. Experimental Procedures.

Preparation of Hydrazones.

All hydrazones were prepared accordingly to the reported procedure. [2]

Preparation of Catalysts.

Catalysts **2a-d** were prepared accordingly to our published procedure. [3]

Catalysts **2e**,**f** were prepared accordingly to our published procedure. [4]

Catalytic Asymmetric Transfer Hydrogenation with Trichlorosilane.

Representative Procedure:

(*R*)-*N*'-(1-Phenylethyl)benzohydrazide (3a):

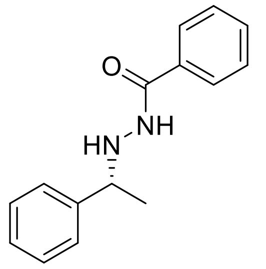

A flame-dried test tube with a magnetic stir bar was charged with hydrazone **1a** (60 mg, 0.25 mmol), catalyst **2a** (15 mg, 0.025 mmol) and CH_2Cl_2 (1.0 mL), cooled to –50 °C, and then treated slowly with a solution of $HSiCl_3$ in CH_2Cl_2 (250 µL, 1.5 M). The reaction mixture was stirred at –40 °C for 20 hours, and then quenched by pouring it into 30 mL of saturated aqueous $NaHCO_3$ solution cooled to 0 °C. The resulting mixture was vigorously stirred for 30 min at room temperature and extracted twice with 15 mL of CH_2Cl_2. The combined organic layers were dried over Na_2SO_4, filtered, and condensed *in vacuo*. A ^{1}H NMR spectrum of the crude reaction mixture was taken with 1,1,2,2-tetracholoroethane as an internal standard (48% NMR yield). A fraction of the crude mixture was purified by prep TLC using 20% EtOAc in CH_2Cl_2 as an eluent for characterization purposes.

All spectral data were consistent with the literature values.[2]

^{1}H NMR (400 MHz, $CDCl_3$) δ 7.63-7.60 (m, 2H), 7.50-7.46 (m, 1H), 7.42-7.34 (m, 7H), 7.32-7.27 (m, 1H), 5.10 (br d, *J* = 4.8 Hz, 1H), 4.26 (q, *J* = 6.8 Hz, 1H), 1.44 (d, *J* = 6.8 Hz, 3H).

ee = 53 %; $[\alpha]^{22}_{D}$ = +7.4 (c = 0.00067, CH_2Cl_2); The enantiomeric excess and the absolute stereochemistry were determined by HPLC analysis:[2] t_R (major) = 19.95 min; t_R (minor) = 29.01 min (Daicel Chiralcel® OJ-H with an OJ-H guard column, hexane/2-propanol = 90:10, 0.5mL/min).

The 1.0 mmol scale reaction:

A flame-dried Schlenck tube with a magnetic stir bar was charged with hydrazone **1a** (238 mg, 1.00 mmol), catalyst **2a** (60 mg, 0.10 mmol) and CH_2Cl_2 (4.0 mL), cooled to –50 °C, and then treated slowly with a solution of $HSiCl_3$ in CH_2Cl_2 (1.0 mL, 1.5 M). The reaction mixture was stirred at –40 °C for 20 hours, and then quenched by pouring it into 120 mL of saturated aqueous $NaHCO_3$ solution cooled to 0 °C. The resulting mixture was vigorously stirred for 30 min. at room temperature and extracted twice with 60 mL of CH_2Cl_2. The combined organic layers were dried over Na_2SO_4, filtered, and condensed *in vacuo*. A ^{1}H NMR spectrum of the crude reaction mixture was taken with 1,1,2,2-tetracholoroethane as an internal standard (40% NMR yield). The crude material was purified by flash column chromatography on silica gel with 2% Et_2O in CH_2Cl_2 to afford the title compound as a white solid (90 mg, 37%), followed by 50% EtOAc in CH_2Cl_2 to recover catalyst **2a** (60 mg, >99%).

(+)-*tert*-Butyl 2-(1-phenylethyl)hydrazinecarboxylate (3b):

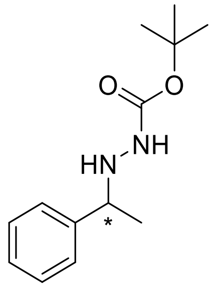

A ^{1}H NMR spectrum of the crude reaction mixture was taken with 1,1,2,2-tetracholoroethane as an internal standard (36% NMR yield). A fraction of the crude

mixture was purified by prep TLC using 5% EtOAc in CH_2Cl_2 as an eluent for characterization purposes.

All spectral data were consistent with the literature values.[5]

^{1}H NMR (400 MHz, $CDCl_3$) δ 7.35-7.26 (m, 5H), 5.97 (br s, 1H), 4.18 (br s, 2H), 1.44 (s, 9H), 1.33 (d, J = 6.8 Hz, 3H).

ee = approximately 71 %; $[\alpha]^{23}_{D}$ = +39.3 (c = 0.00067, CH_2Cl_2); The enantiomeric excess was estimated by HPLC analysis as both enantiomers were not fully separated at the base line: t_R (major) = 20.63 min; t_R (minor) = 17.25 min (Daicel Chiralcel® OJ-H with an OJ-H guard column, hexane/2-propanol = 99:1, 0.5mL/min).

(*R*)-Benzyl-2-(1-phenylethyl)hydrazine-1-carboxylate (3c):

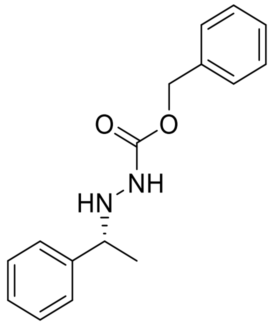

A ^{1}H NMR spectrum of the crude reaction mixture was taken with 1,1,2,2-tetracholoroethane as an internal standard (21% NMR yield). A fraction of the crude mixture was purified by prep TLC using 5% EtOAc in CH_2Cl_2 as an eluent for characterization purposes.

All spectral data were consistent with the literature values.[6]

^{1}H NMR (400 MHz, $CDCl_3$) δ 7.35-7.27 (m, 10H), 6.13 (br s, 1H), 5.12 (s, 2H), 4.21 (br s, 2H), 1.34 (d, J = 6.4 Hz, 3H).

ee = 64 %; $[\alpha]^{23}_{D}$ = +60.4 (c = 0.00067, CH_2Cl_2); The enantiomeric excess and the absolute stereochemistry were determined by HPLC analysis:[6] t_R (major) = 43.57 min; t_R (minor) = 33.45 min (Daicel Chiralcel® OJ-H with an OJ-H guard column, hexane/2-propanol = 90:10, 0.5mL/min).

(*R*)-1-Phenyl-1-(2-*p*-nitrobenzoylhydrazino)ethane (3d):

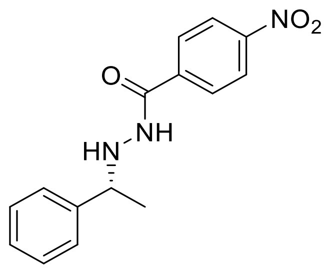

A ^{1}H NMR spectrum of the crude reaction mixture was taken with 1,1,2,2-tetracholoroethane as an internal standard (14% NMR yield). A fraction of the crude mixture was purified by prep TLC using 20% EtOAc in CH_2Cl_2 as an eluent for characterization purposes.

^{1}H NMR (400 MHz, $CDCl_3$) δ 8.24 (d, *J* = 8.8 Hz, 2H), 7.77 (d, *J* = 8.8 Hz, 2H), 7.54 (d, *J* = 5.6 Hz, 1H), 7.41-7.29 (m, 5H), 5.11 (br d, *J* = 4.8 Hz, 1H), 4.26 (q, *J* = 6.4 Hz, 1H), 1.45 (d, *J* = 6.4 Hz, 3H).

^{13}C NMR (100 MHz, $CDCl_3$) δ 165.2, 149.8, 142.7, 138.5, 128.7, 128.1, 127.8, 127.2, 123.9, 60.1, 21.3.

IR (thin film): 3276, 2973, 1642, 1522, 1343, 867, 848, 760, 715, 699 cm^{-1}

HRMS (ESI): Exact mass calculated for $C_{15}H_{16}N_3O_3^+$ $[M+H]^+$ expected: 286.1186, found: 286.1188.

ee = 69 %; [α] ^{23} $_{D}$ = +84.9 (c = 0.00067, CH_2Cl_2); The enantiomeric excess and the absolute stereochemistry were determined by HPLC analysis:[7] t_R (major) = 57.56 min; t_R (minor) = 51.44 min (Daicel Chiralcel® OJ-H with an OJ-H guard column, hexane/2-propanol = 90:10, 0.5mL/min).

(*R*)-*N'*-(1-(*p*-Tolyl)ethyl)benzohydrazide (3e):

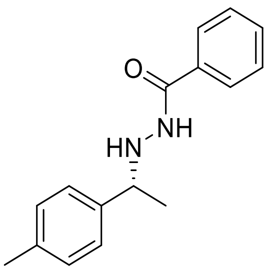

A ^{1}H NMR spectrum of the crude reaction mixture was taken with 1,1,2,2-tetracholoroethane as an internal standard (27% NMR yield). A fraction of the crude mixture was purified by prep TLC using 20% EtOAc in CH_2Cl_2 as an eluent for characterization purposes.

All spectral data were consistent with the literature values.[2]

^{1}H NMR (400 MHz, $CDCl_3$) δ 7.64-7.61 (m, 2H), 7.51-7.47 (m, 1H), 7.41-7.38 (m, 3H), 7.30 (d, *J* = 8.0 Hz, 2H), 7.17 (d, *J* = 8.0 Hz, 2H), 5.09 (b rs, 1H), 4.23 (q, *J* = 6.8 Hz, 1H), 2.36 (s, 3H), 1.42 (d, *J* = 6.8 Hz, 3H).

ee = 57 %; [α] ^{23} $_{D}$ = +8.5 (c = 0.00067, CH_2Cl_2); The enantiomeric excess and the absolute stereochemistry were determined by HPLC analysis:[2] t_R (major) = 17.27 min; t_R (minor) = 31.13 min (Daicel Chiralcel® OJ-H with an OJ-H guard column, hexane/2-propanol = 90:10, 0.5mL/min).

(*R*)-*N*'-(1-(m-Tolyl)ethyl)benzohydrazide (3f):

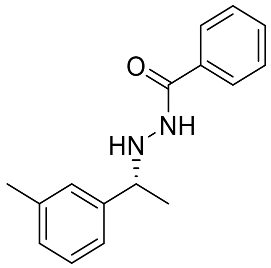

A ^{1}H NMR spectrum of the crude reaction mixture was taken with 1,1,2,2-tetracholoroethane as an internal standard (49% NMR yield). A fraction of the crude mixture was purified by prep TLC using 20% EtOAc in CH_2Cl_2 as an eluent for characterization purposes.

All spectral data were consistent with the literature values.[2]

^{1}H NMR (400 MHz, $CDCl_3$) δ 7.63 (d, *J* = 7.2 Hz, 2H), 7.51-7.38 (m, 4H), 7.26-7.19 (m, 3H), 7.11 (d, *J* = 7.2 Hz, 1H), 5.10 (br s, 1H), 4.22 (q, *J* = 6.8 Hz, 1H), 2.36 (s, 3H), 1.43 (d, *J* = 6.8 Hz, 3H).

ee = 41 %; $[\alpha]^{22}_{D}$ = +29.0 (c = 0.0013, CH_2Cl_2); The enantiomeric excess and the absolute stereochemistry were determined by HPLC analysis:[2] t_R (major) = 25.65 min; t_R (minor) = 29.36 min (Daicel Chiralcel® OJ-H with an OJ-H guard column, hexane/2-propanol = 95:5, 0.5mL/min).

(*R*)-*N*'-(1-(4-Methoxyphenyl)ethyl)benzohydrazide (3h):

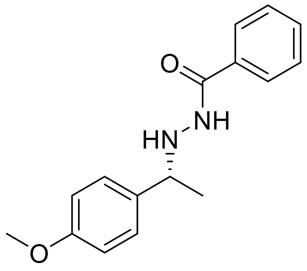

A ^{1}H NMR spectrum of the crude reaction mixture was taken with 1,1,2,2-tetracholoroethane as an internal standard (12% NMR yield). A fraction of the crude mixture was purified by prep TLC using 20% EtOAc in CH_2Cl_2 as an eluent for characterization purposes.

All spectral data were consistent with the literature values.[2]

^{1}H NMR (400 MHz, $CDCl_3$) δ 7.64-7.61 (m, 2H), 7.51-7.47 (m, 1H), 7.42-7.38 (m, 3H), 7.34-7.31 (m , 2H), 6.91-6.88 (m, 2H), 5.08 (b rs, 1H), 4.22 (q, *J* = 6.8 Hz, 1H), 3.82 (s, 3H), 1.42 (d, *J* = 6.8 Hz, 3H).

ee = 58 %; $[\alpha]^{22}_{D}$ = +23.8 (c = 0.0013, CH_2Cl_2); The enantiomeric excess and the absolute stereochemistry were determined by HPLC analysis:[2] t_R (major) = 35.59 min; t_R (minor) = 59.24 min (Daicel Chiralcel® OJ-H with an OJ-H guard column, hexane/2-propanol = 90:10, 0.5mL/min).

(*R*)-*N'*-(1-(4-Fluorophenyl)ethyl)benzohydrazide (3i):

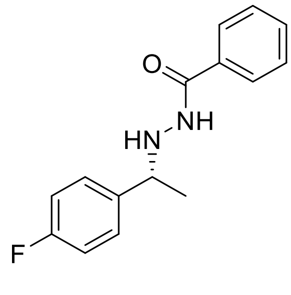

A ^{1}H NMR spectrum of the crude reaction mixture was taken with 1,1,2,2-tetracholoroethane as an internal standard (28% NMR yield). A fraction of the crude mixture was purified by prep TLC using 20% EtOAc in CH_2Cl_2 as an eluent for characterization purposes.

All spectral data were consistent with the literature values.[2]

^{1}H NMR (400 MHz, $CDCl_3$) δ 7.63-7.61 (m, 2H), 7.52-7.35 (m, 6H), 7.04 (dd, J= 8.4, 8.4 Hz, 2H), 5.06 (br s, 1H), 4.26 (q, *J* = 6.8 Hz, 1H), 1.41 (d, *J* = 6.8 Hz, 3H).

ee = 37 %; $[\alpha]^{23}_{D}$ = +3.4 (c = 0.00067, CH_2Cl_2); The enantiomeric excess and the absolute stereochemistry were determined by HPLC analysis:[2] t_R (major) = 21.37 min; t_R (minor) = 27.53 min (Daicel Chiralcel® OJ-H with an OJ-H guard column, hexane/2-propanol = 90:10, 0.5mL/min).

(*R*)-*N'*-(1-(4-Chlorophenyl)ethyl)benzohydrazide (3j):

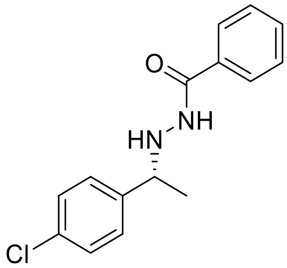

A ^{1}H NMR spectrum of the crude reaction mixture was taken with 1,1,2,2-tetracholoroethane as an internal standard (22% NMR yield). A fraction of the crude mixture was purified by prep TLC using 20% EtOAc in CH_2Cl_2 as an eluent for characterization purposes.

All spectral data were consistent with the literature values.[2]

^{1}H NMR (400 MHz, $CDCl_3$) δ 7.62 (d, *J* = 7.2 Hz, 2H), 7.52-7.48 (m, 1H), 7.42-7.29 (m, 7H), 5.05 (br d, *J* = 4.8 Hz, 1H), 4.27-4.23 (m, 1H), 1.41 (d, *J* = 6.4 Hz, 3H).

ee = 43 %; $[\alpha]^{23}_{D}$ = +50.4 (c = 0.00067, CH_2Cl_2); The enantiomeric excess and the absolute stereochemistry were determined by HPLC analysis:[2] t_R (major) = 20.97 min; t_R (minor) = 26.49 min (Daicel Chiralcel® OJ-H with an OJ-H guard column, hexane/2-propanol = 90:10, 0.5mL/min).

(*R*)-*N'*-(1-(4-Bromophenyl)ethyl)benzohydrazide (3k):

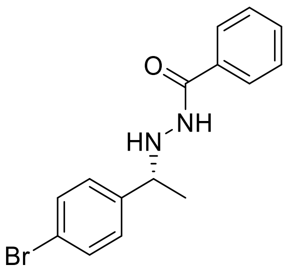

A ^{1}H NMR spectrum of the crude reaction mixture was taken with 1,1,2,2-tetracholoroethane as an internal standard (27% NMR yield). A fraction of the crude mixture was purified by prep TLC using 20% EtOAc in CH_2Cl_2 as an eluent for characterization purposes.

All spectral data were consistent with the literature values.[2]

^{1}H NMR (400 MHz, $CDCl_3$) δ 7.62 (d, *J* = 7.6 Hz, 2H), 7.52-7.39 (m, 6H), 7.29 (d, *J* = 8.4 Hz, 2H), 5.06 (br s, 1H), 4.24 (q, *J* = 6.4 Hz, 1H), 1.40 (d, *J* = 6.4 Hz, 3H).

ee = 44 %; $[\alpha]^{23}_{D}$ = +50.7 (c = 0.002, CH_2Cl_2); The enantiomeric excess and the absolute stereochemistry were determined by HPLC analysis:[2] t_R (major) = 23.36 min; t_R (minor) = 29.21 min (Daicel Chiralcel® OJ-H with an OJ-H guard column, hexane/2-propanol = 90:10, 0.5mL/min).

(*R*)-*N'*-(1-(4-(Trifluoromethyl)phenyl)ethyl)benzohydrazide (3l):

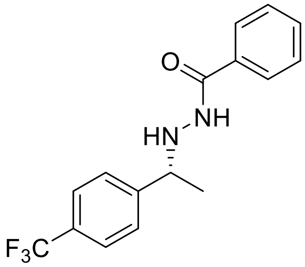

A ^{1}H NMR spectrum of the crude reaction mixture was taken with 1,1,2,2-tetracholoroethane as an internal standard (37% NMR yield). A fraction of the crude mixture was purified by prep TLC using 20% EtOAc in CH_2Cl_2 as an eluent for characterization purposes.

All spectral data were consistent with the literature values.[2]

^{1}H NMR (400 MHz, $CDCl_3$) δ 7.63-7.61 (m, 4H), 7.53 (d, *J* = 8.0 Hz, 2H), 7.51-7.48 (m, 1H), 7.42-7.38 (m, 3H), 5.07 (br d, *J* = 6.8 Hz, 1H), 4.37-4.32 (m, 1H), 1.44 (d, *J* = 6.8 Hz, 3H).

ee = 35 %; $[\alpha]^{23}_{D}$ = +56.1 (c = 0.00067, CH_2Cl_2); The enantiomeric excess and the absolute stereochemistry were determined by HPLC analysis:[6]: t_R (major) = 28.48 min; t_R (minor) = 25.77 min (Daicel Chiralpak® AS-H with an AS-H guard column, hexane/2-propanol = 80:20, 0.5mL/min).

(*R*)-*N'*-(1-(Naphthalen-2-yl)ethyl)benzohydrazide (3m):

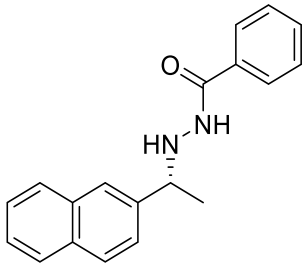

A ^{1}H NMR spectrum of the crude reaction mixture was taken with 1,1,2,2-tetracholoroethane as an internal standard (38% NMR yield). A fraction of the crude mixture was purified by prep TLC using 20% EtOAc in CH_2Cl_2 as an eluent for characterization purposes.

All spectral data were consistent with the literature values.[8]

^{1}H NMR (400 MHz, $CDCl_3$) δ 7.87-7.81 (m, 4H), 7.61-7.56 (m, 3H), 7.50-7.44 (m, 4H), 7.37-7.34 (m, 2H), 5.19 (br d, J = 5.2 Hz, 1H), 4.43 (q, J = 6.8 Hz, 1H), 1.51 (d, J = 6.8 Hz, 3H).

ee = 43 %; $[\alpha]^{23}_{D}$ = +82.4 (c = 0.00067, CH_2Cl_2); The enantiomeric excess and the absolute stereochemistry were determined by HPLC analysis:[8] t_R (major) = 36.05 min; t_R (minor) = 43.91 min (Daicel Chiralcel® OJ-H with an OJ-H guard column, hexane/2-propanol = 85:15, 0.5mL/min).

(*R*)-*N'*-(1-(Thiophen-2-yl)ethyl)benzohydrazide (3n):

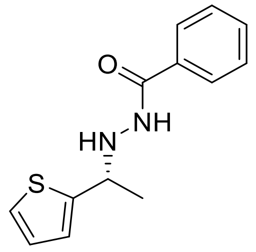

A ^{1}H NMR spectrum of the crude reaction mixture was taken with 1,1,2,2-tetracholoroethane as an internal standard (33% NMR yield). A fraction of the crude mixture was purified by prep TLC using 20% EtOAc in CH_2Cl_2 as an eluent for characterization purposes.

All spectral data were consistent with the literature values.[2]

^{1}H NMR (400 MHz, $CDCl_3$) δ 7.67 (d, J = 7.2 Hz, 2H), 7.53-7.49 (m, 2H), 7.44-7.40 (m, 2H), 7.28-7.26 (m, 1H), 7.00-6.97 (m, 2H), 5.14 (br d, J = 4.8 Hz, 1H), 4.60-4.56 (m, 1H), 1.53 (d, J = 6.8 Hz, 3H).

ee = 38 %; $[\alpha]^{23}_{D}$ = +32.5 (c = 0.001, CH_2Cl_2); The enantiomeric excess and the absolute stereochemistry were determined by HPLC analysis:[2] t_R (major) = 26.72 min;

t_R (minor) = 32.39 min (Daicel Chiralcel® OJ-H with an OJ-H guard column, hexane/2-propanol = 90:10, 0.5mL/min).

(*R*)-*N'*-(1-Phenylpropyl)benzohydrazide (3o):

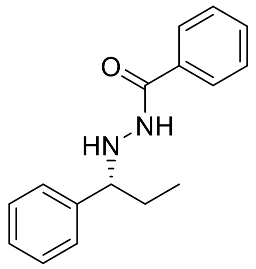

A ^{1}H NMR spectrum of the crude reaction mixture was taken with 1,1,2,2-tetracholoroethane as an internal standard (44% NMR yield). A fraction of the crude mixture was purified by prep TLC using 20% EtOAc in CH_2Cl_2 as an eluent for characterization purposes, which provided 2.5 mg of the title compound. The remaining crude mixture was purified by flash column chromatography on silica gel with 2% Et_2O in CH_2Cl_2 to afford 23 mg of the title compound (total 25.5 mg, 40%).

All spectral data were consistent with the literature values.[6]

^{1}H NMR (400 MHz, $CDCl_3$) δ 7.60-7.58 (m, 2H), 7.49-7.46 (m, 1H), 7.40-7.33 (m, 7H), 7.32-7.27 (m, 1H), 5.18 (br d, *J* = 5.6 Hz, 1H), 4.00 (br dd, *J* = 6.8, 7.2 Hz, 1H), 1.94-1.83 (m, 1H), 1.77-1.66 (m, 1H), 0.87 (t, *J* = 7.6 Hz, 3H).

ee = 36 %; $[\alpha]^{23}_{D}$ = +37.2 (c = 0.0013, CH_2Cl_2); The enantiomeric excess and the absolute stereochemistry were determined by HPLC analysis:[6] t_R (major) = 16.49 min; t_R (minor) = 19.55 min (Daicel Chiralcel® OJ-H with an OJ-H guard column, hexane/2-propanol = 90:10, 0.5mL/min).

(*S*)-*N'*-(1-Cyclohexylethyl)benzohydrazide (3p):

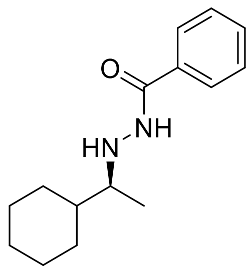

A ^{1}H NMR spectrum of the crude reaction mixture was taken with 1,1,2,2-tetracholoroethane as an internal standard (33% NMR yield). A fraction of the crude mixture was purified by prep TLC using 20% EtOAc in CH_2Cl_2 as an eluent for characterization purposes.

All spectral data were consistent with the literature values.[9]

^{1}H NMR (400 MHz, $CDCl_3$) δ 7.76-7.74 (m, 2H), 7.54-7.43 (m, 4H), 4.89 (br s, 1H), 2.94-2.88 (m, 1H), 1.78-1.67 (m, 6H), 1.47-1.40 (m, 1H), 1.31-1.09 (m, 4H), 1.06 (d, *J* = 6.4 Hz, 3H).

ee = 46 %; $[\alpha]^{21}_{D}$ = +4.1 (c = 0.001, CH_2Cl_2); The enantiomeric excess and the absolute stereochemistry were determined by HPLC analysis:[9] t_R (major) = 36.08 min; t_R (minor) = 41.83 min (Daicel Chiralcel® OJ-H with an OJ-H guard column, hexane/2-propanol = 99:1, 0.5mL/min).

(+)-*N'*-(4-Phenylbutan-2-yl)benzohydrazide (3q):

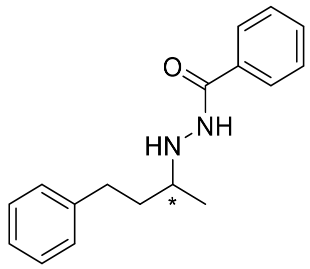

A ^{1}H NMR spectrum of the crude reaction mixture was taken with 1,1,2,2-tetracholoroethane as an internal standard (48% NMR yield). A fraction of the crude mixture was purified by prep TLC using 20% EtOAc in CH_2Cl_2 as an eluent for characterization purposes, which provided 3 mg of the title compound. The remaining crude mixture was purified by flash column chromatography on silica gel with 2% Et_2O in CH_2Cl_2 and then with 5% Et_2O in CH_2Cl_2 to afford 26 mg of the title compound (total 29 mg, 43%).

^{1}H NMR (400 MHz, $CDCl_3$) δ 7.74-7.72 (m, 2H), 7.55-7.43 (m, 4H), 7.31-7.17 (m, 5H), 4.91 (br s, 1H), 3.17-3.12 (m, 1H), 2.80-2.65 (m, 2H), 1.94-1.85 (m, 1H), 1.73-1.61 (m, 1H), 1.18 (d, J = 6.4 Hz, 3H).

^{13}C NMR (100 MHz, $CDCl_3$) δ 167.5, 142.1, 132.9, 131.8, 128.7, 128.4, 128.3, 126.8, 125.8, 55.6, 36.7, 32.1, 18.6.

IR (thin film): 3315, 1640, 1539, 1472, 1456, 1377, 905, 894, 854, 729, 696 cm^{-1}

HRMS (ESI): Exact mass calculated for $C_{17}H_{21}N_2O^+$ $[M+H]^+$ expected: 269.1648, found: 269.1652.

ee = 14 %; $[\alpha]^{21}_{D}$ = +4.7 (c = 0.002, CH_2Cl_2); The enantiomeric excess was determined by HPLC analysis: t_R (major) = 37.17 min; t_R (minor) = 30.40 min (Daicel Chiralcel® OD-H with an OD-H guard column, hexane/2-propanol = 90:10, 0.5mL/min).

(*R*)-*N'*-(4-Phenyl-3-buten-2-yl)benzohydrazide (3r):

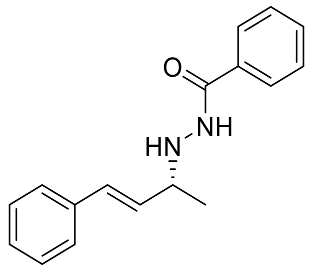

A ^{1}H NMR spectrum of the crude reaction mixture was taken with 1,1,2,2-tetracholoroethane as an internal standard (**3r:** 4% NMR yield; **3q**: 19% NMR yield). A fraction of the crude mixture was purified by prep TLC using 20% EtOAc in CH_2Cl_2 as an eluent for characterization purposes. Compounds **3r** and **3q** were isolated as an inseparable mixture.

The mixture of **3r** and **3q** was analyzed by LC/MS (ES-APCI): t_R (**3r**) = 31.567 min, calculated for $C_{17}H_{19}N_2O^+$ $[M+H]^+$ expected: 267.1, found: 267.2; t_R (**3q**) = 31.344 min, calculated for $C_{17}H_{21}N_2O^+$ $[M+H]^+$ expected: 269.2, found: 269.2 (LC column: Poroshell120-EC-C18 43.0*50 mm, 2.7 um; 24 °C; H_2O/Methanol=90:10; 0.5 ml/min). Racemic **3r** was prepared by sodium cyanoborohydride reduction of the corresponding hydrazone, which also produced **3q** (**3r**:**3q** = *ca.* 4:1). While enantiomers of **3q** were fully separated on Daicel Chiralcel® OD-H (*vide supra*), enantiomers of **3r** were not separable from those of **3q**. We found out that enantiomers of **3r** were separable from those of **3q** under following HPLC condition: Daicel Chiralpak® AD-H with an AD-H guard column, hexane/2-propanol = 92:8,0.5mL/min. The first peak (t_R = 36.56) and second peak (t_R = 40.21) were the corresponding enantiomers of **3r**, and the third peak (t_R = 45.41) and fourth peak (t_R = 48.85) were the corresponding enantiomers of **3q**. Therefore, we were able to determine the ee of **3r**, as its enantiomers were fully

separated at the base line on the Daicel Chiralpak® AD-H column. However, the enantiomeric excess of **3q** was provided as an estimated value, for the enantiomers of **3q** were not fully separated at the base line by chiral HPLC on the Daicel Chiralpak® AD-H column.

3r : ee = 15 %; The enantiomeric excess and the absolute stereochemistry were determined by HPLC analysis:[10] t_R (major) = 40.47 min; t_R (minor) = 36.56 min (Daicel Chiralcel® AD-H with an AD-H guard column, hexane/2-propanol = 92:8, 0.5mL/min)

3q : ee = approximately 7 %; The enantiomeric excess was estimated by HPLC analysis as both enantiomers were not fully separated at the base line: t_R (major) = 45.25 min; t_R (minor) = 48.99 min (Daicel Chiralpak® AD-H with an AD-H guard column, hexane/2-propanol = 92:8, 0.5mL/min).

3. Computational Procedures.

All calculations have been performed with the Q-Chem 4 quantum chemistry code,[11] using the PBEh-3c density functional theory composite procedure.[12] Solvent effects have been included using the C-PCM method with the dielectric constant of DCM (ε = 9.08). Minima are converged within a threshold of 10^{-8} E_h, and have been confirmed minima using frequency calculations. Molecular symmetry was not used because of the necessity of the C-PCM method. The C_2 symmetry expected for the two main minima (complex 1 and complex 2) is respected in an approximate manner. The initial structures for the geometry optimizations have been obtained via a molecular mechanics based conformational search algorithm, developed in-house. Given the fact that the molecules in this project and the nature of their interaction are well within the

limits of recent validation studies of the method that we used, we expect geometries to be converged within 0.05 Å accuracy,[14] and energies to be converged within 2 kcal/mol accuracy.[15] The structures of the minima for the complex and the free Lewis bases are also provided in cartesian coordinates (xyz) as additional supplementary text files.

4. References:

[1] Still, W. C.; Kahn, M.; Mitra A. *J. Org. Chem.* **1978**, *43*, 2923-2925.

[2] Hu, Y.; Zhang, Z.; Zhang, J.; Liu, Y.; Gridnev, D.; Zhang, W. *Angew. Chem. Int. Ed.* **2019**, *58*, 15767-15771.

[3] Sun, S.; Reep, C.; Zhang, C.; Captain, B.; Peverati, R.; Takenaka, N. *Tetrahedron Letters* (*in press*)

[4] Reep, C.; Morgante, P.; Peverati, R.; Takenaka, N. *Org. Lett.* **2018**, *20*, 5757-5761.

[5] Yoshikawa, N.; Tan, L.; McWilliams, J. C.; Ramasamy, D.; Sheppard, R. *Org. Lett.* **2010**, *12*, 276-279.

[6] Yang, P.; Zhang, C.; Ma, Y.; Zhang, C.; Li, A.; Tang, B.; Zhou, J. *Angew. Chem. Int. Ed.* **2017**, *56*, 14702-14706.

[7] Burk, M. J.; Martinez, J. P.; Feaster, J. E.; Cosford, N. *Tetrahedron* **1994**, *50*, 4399-4428.

[8] Chang, M.; Liu, S.; Huang, K.; Zhang, X. *Org. Lett.* **2013**, *15*, 4354-4357.

[9] Yang, P.; Lim, Li H.; Chuanprasit, P.; Hirao, H.; Zhou, J. *Angew. Chem. Int. Ed.* **2016**, *55*, 12083-12087.

[10] Wang, Y.; Xu, J.; Gu, Y.; Tian, S. *Org. Chem. Front.* **2014**, *1*, 812-816.

[11] Shao, Y.; *et al.*, *Mol. Phys.* **2015**, *113*, 184-215.

[12] Grimme, S.; Brandenburg, G; Bannwarth, C.; Hansen, A., *J. Chem. Phys.* **2015**, *143*, 054107.

[13] Barone, V.; Cossi, M., *J. Phys. Chem. A* **1998**, 1*02*, 1995; Truong, T. N.; Stefanovich, E. V., **Chem. Phys. Lett.**, **1995**, *240*, 253.

[14] Morgante, P.; Peverati, R., *Chem. Phys. Lett.* **2021**, *765*, 138281.

[15] Morgante, P.; Peverati, R., *Phys. Chem. Chem. Phys. Lett.* **2019**, *21*, 19092-19103.

Ph

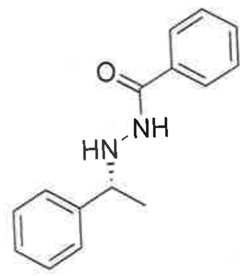

3a

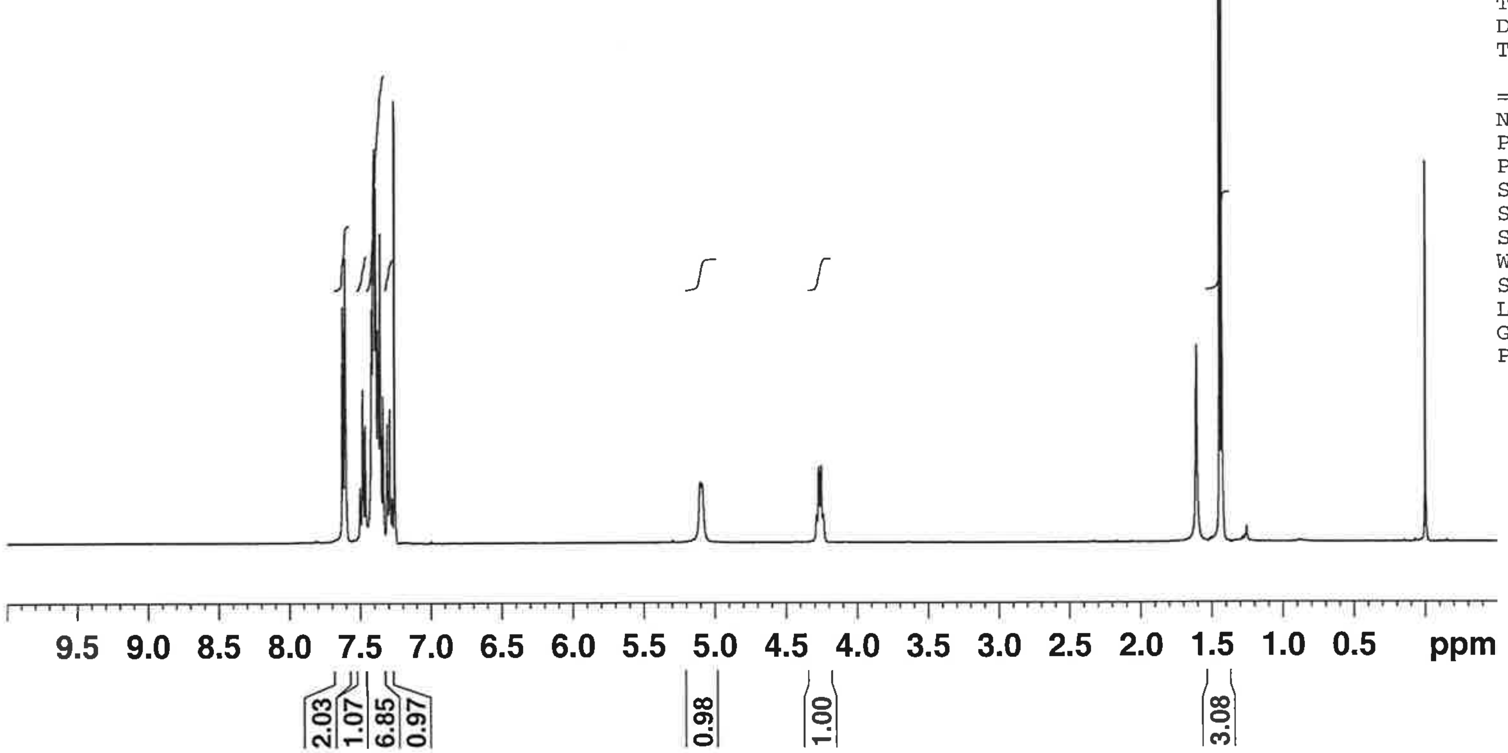

NAME	Aug19-2021-sys
EXPNO	10
PROCNO	1
Date_	20210819
Time	13.36
INSTRUM	spect
PROBHD	5 mm PABBO BB-
PULPROG	zg30
TD	65536
SOLVENT	CDCl3
NS	16
DS	2
SWH	8278.146 Hz
FIDRES	0.126314 Hz
AQ	3.9584243 sec
RG	322.5
DW	60.400 usec
DE	6.50 usec
TE	296.6 K
D1	1.00000000 sec
TD0	1

======== CHANNEL f1 ========

NUC1	1H
P1	11.60 usec
PL1	3.00 dB
SFO1	400.1324710 MHz
SI	32768
SF	400.1300098 MHz
WDW	EM
SSB	0
LB	0.30 Hz
GB	0
PC	1.00

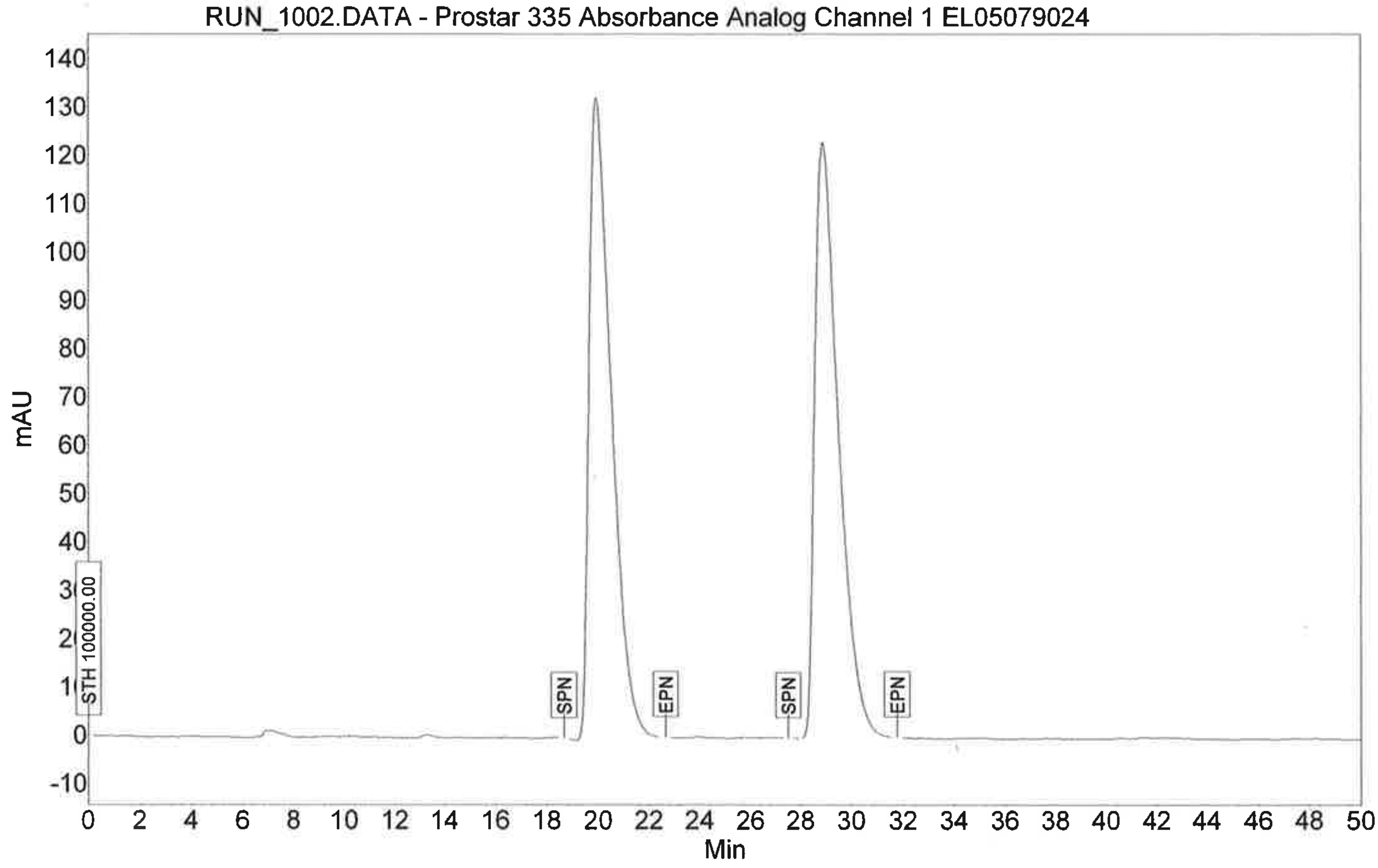

Index	Name	Time [Min]	Quantity [% Area]	Height [mAU]	Area [mAU.Min]	Area % [%]
1	UNKNOWN	19.91	49.78	132.5	132.7	49.783
2	UNKNOWN	28.87	50.22	123.3	133.8	50.217
Total			100.00	255.7	266.5	100.000

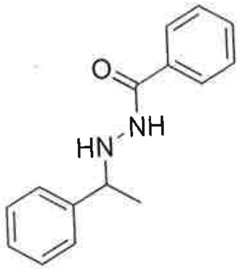

(±)

RUN_1003.DATA - Prostar 335 Absorbance Analog Channel 1 EL05079024

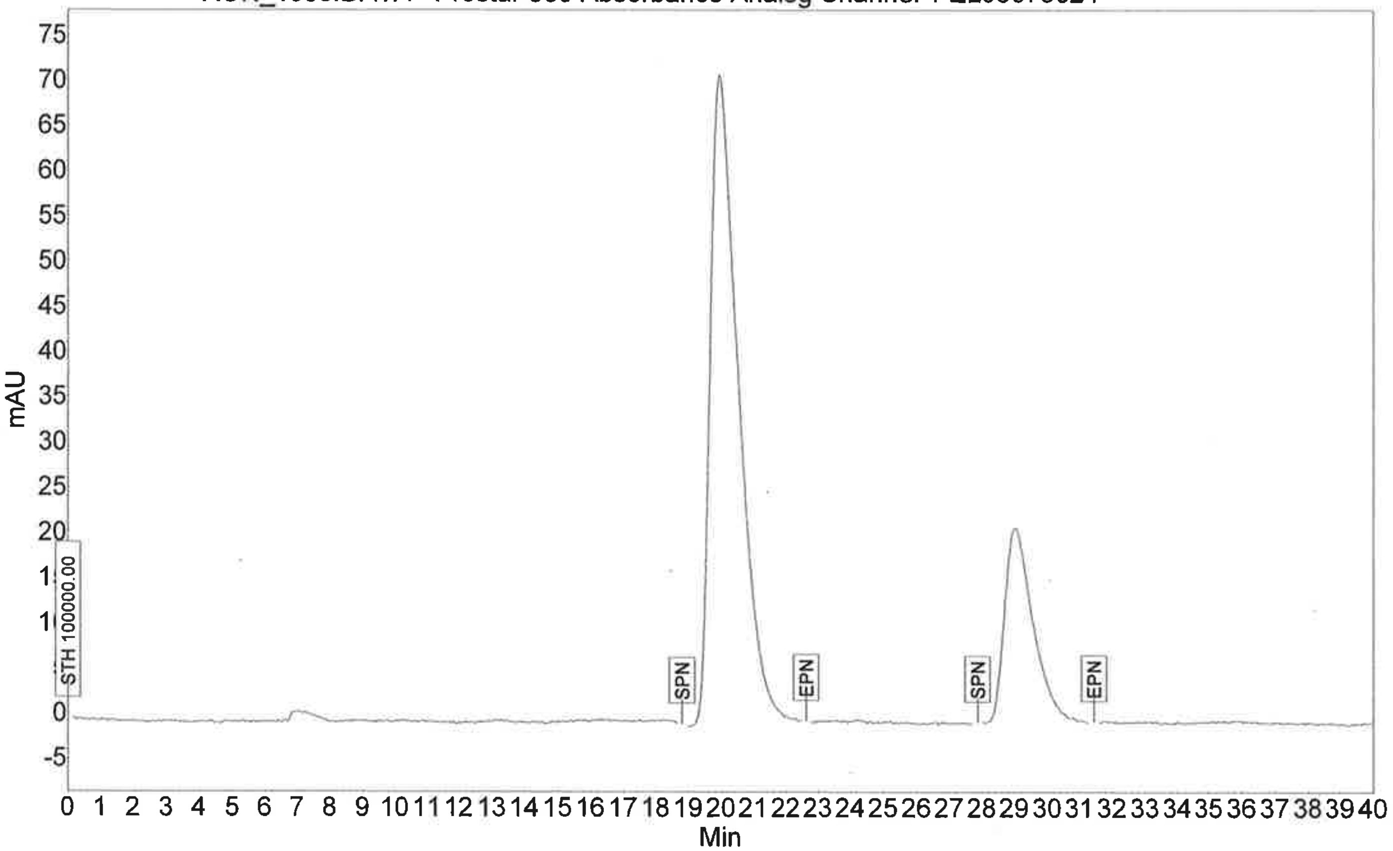

Index	Name	Time [Min]	Quantity [% Area]	Height [mAU]	Area [mAU.Min]	Area % [%]
1	UNKNOWN	19.95	76.72	71.6	71.5	76.715
2	UNKNOWN	29.01	23.28	21.5	21.7	23.285
Total			100.00	93.1	93.2	100.000

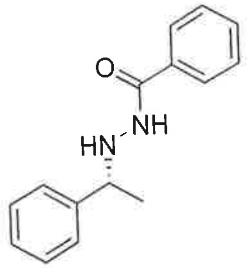

3a

boc

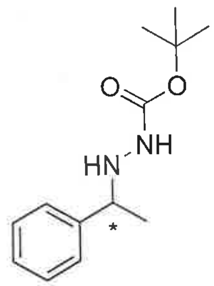

3b

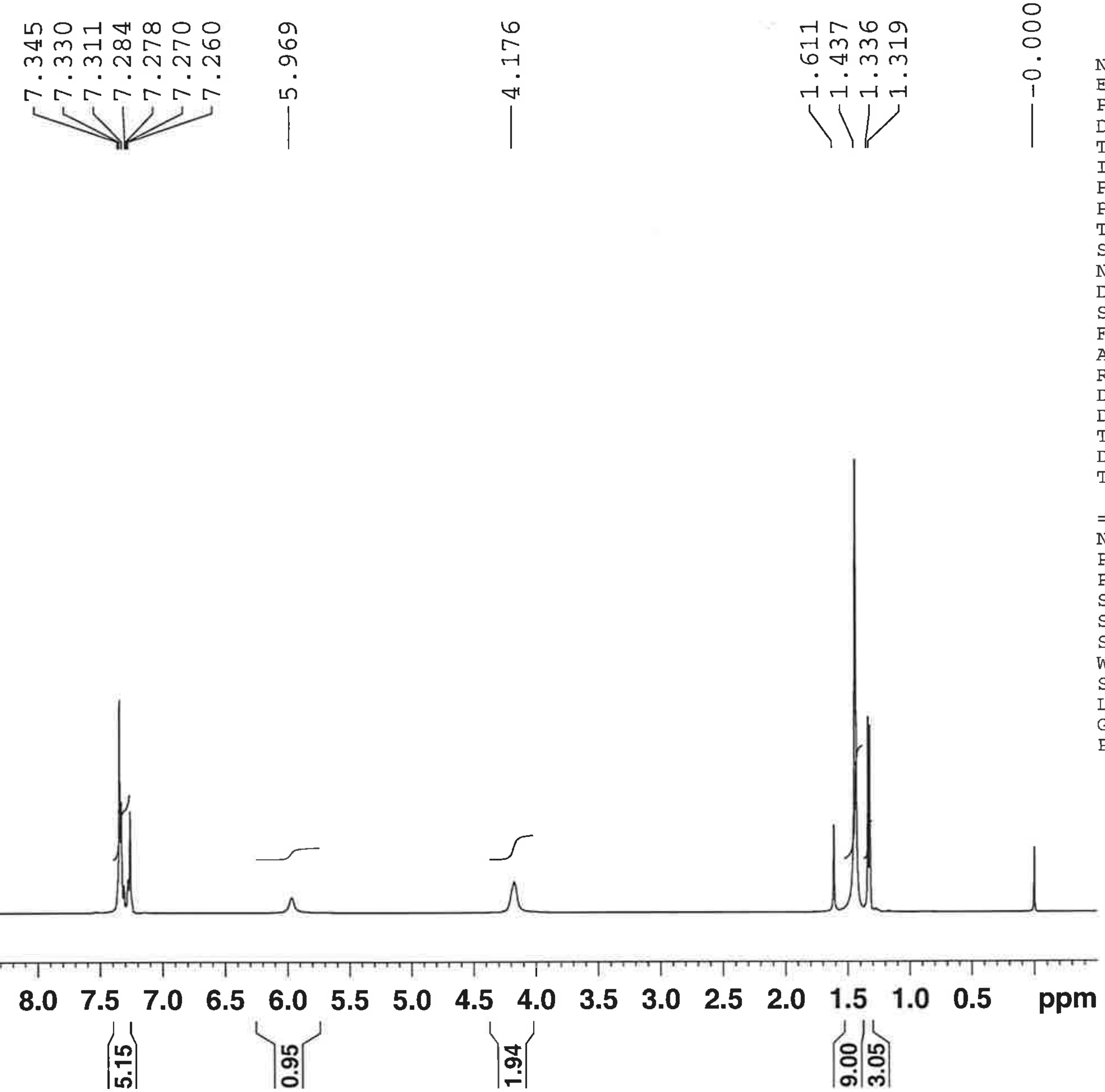

NAME	Aug19-2021-sys	
EXPNO	30	
PROCNO	1	
Date_	20210819	
Time	16.19	
INSTRUM	spect	
PROBHD	5 mm PABBO BB-	
PULPROG	zg30	
TD	65536	
SOLVENT	CDCl3	
NS	16	
DS	2	
SWH	8278.146	Hz
FIDRES	0.126314	Hz
AQ	3.9584243	sec
RG	228.1	
DW	60.400	usec
DE	6.50	usec
TE	296.6	K
D1	1.00000000	sec
TD0	1	

======== CHANNEL f1 ========

NUC1	1H	
P1	11.60	usec
PL1	3.00	dB
SFO1	400.1324710	MHz
SI	32768	
SF	400.1300092	MHz
WDW	EM	
SSB	0	
LB	0.30	Hz
GB	0	
PC	1.00	

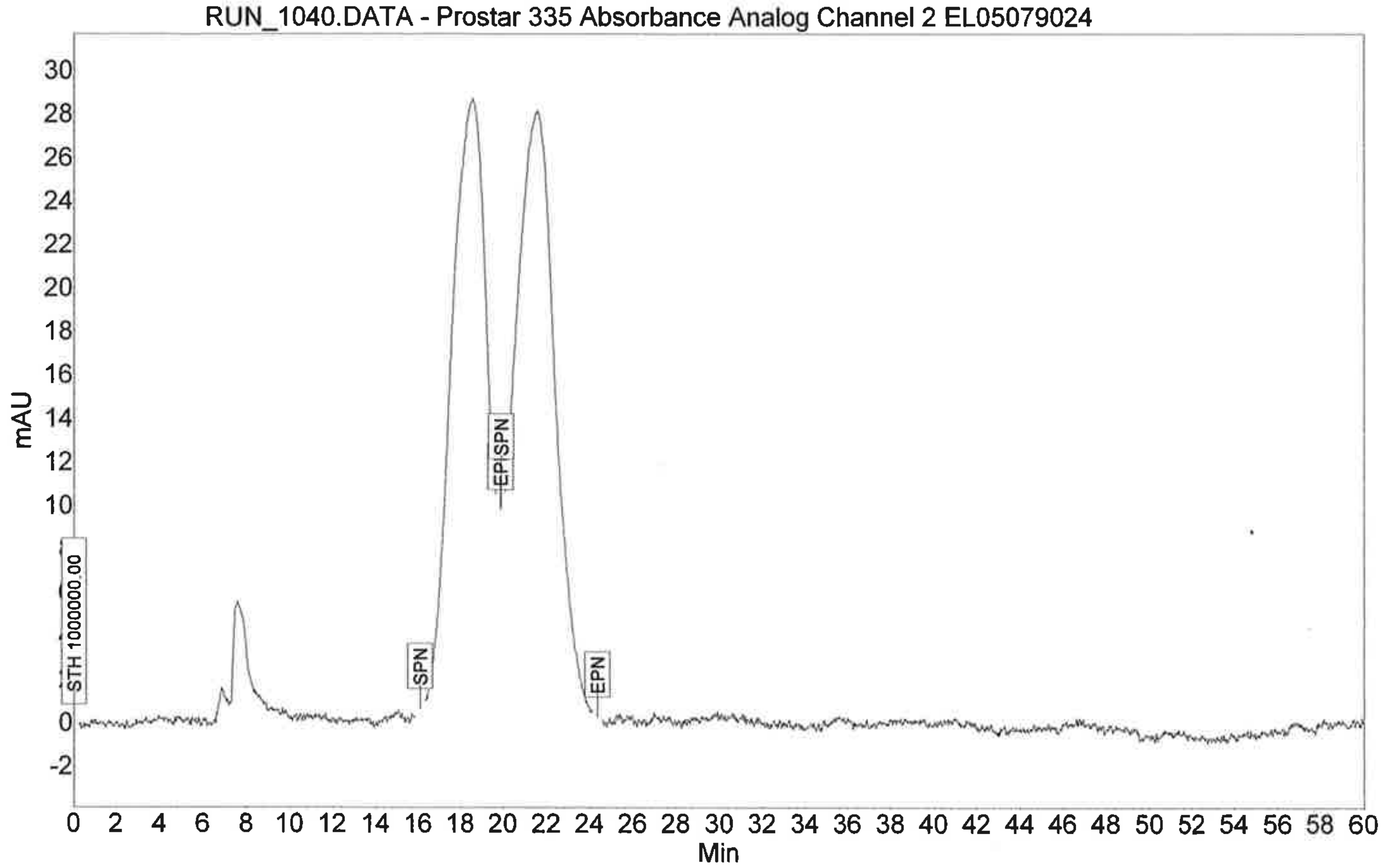

Index	Name	Time [Min]	Quantity [% Area]	Height [mAU]	Area [mAU.Min]	Area % [%]
1	UNKNOWN	18.44	49.82	22.1	38.4	49.821
2	UNKNOWN	21.64	50.18	22.0	38.7	50.179
Total			100.00	44.1	77.1	100.000

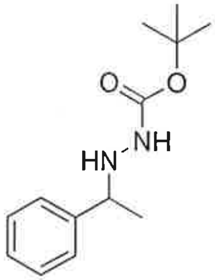

(±)

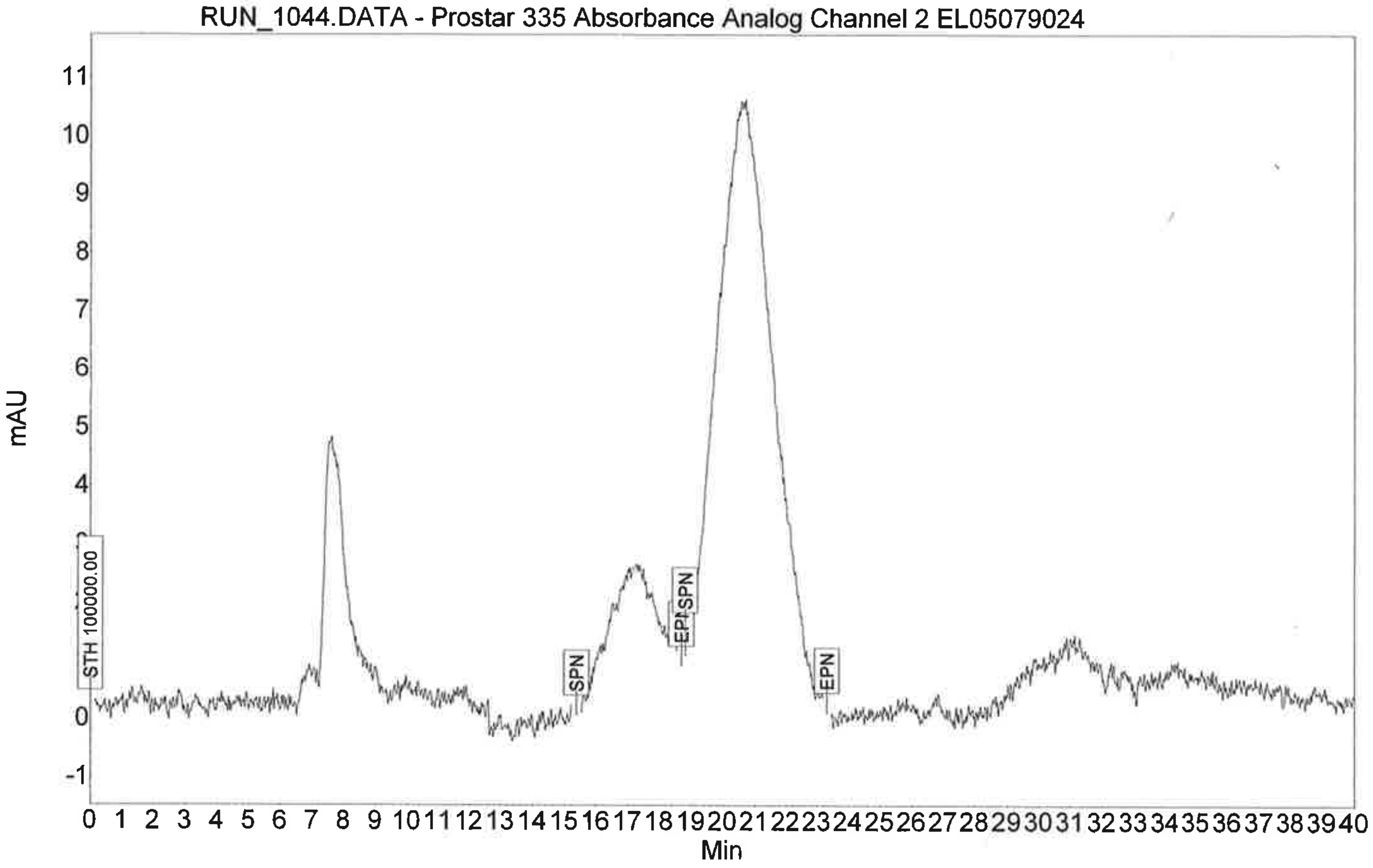

Index	Name	Time [Min]	Quantity [% Area]	Height [mAU]	Area [mAU.Min]	Area % [%]
1	UNKNOWN	17.25	14.43	2.1	3.4	14.426
2	UNKNOWN	20.63	85.57	9.9	20.3	85.574
Total			100.00	12.0	23.7	100.000

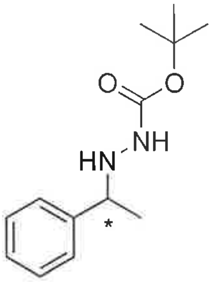

3b

cbz

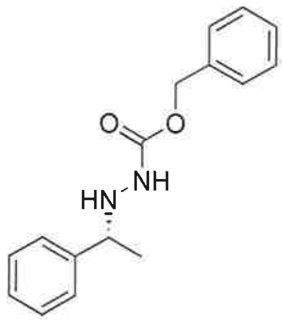

3c

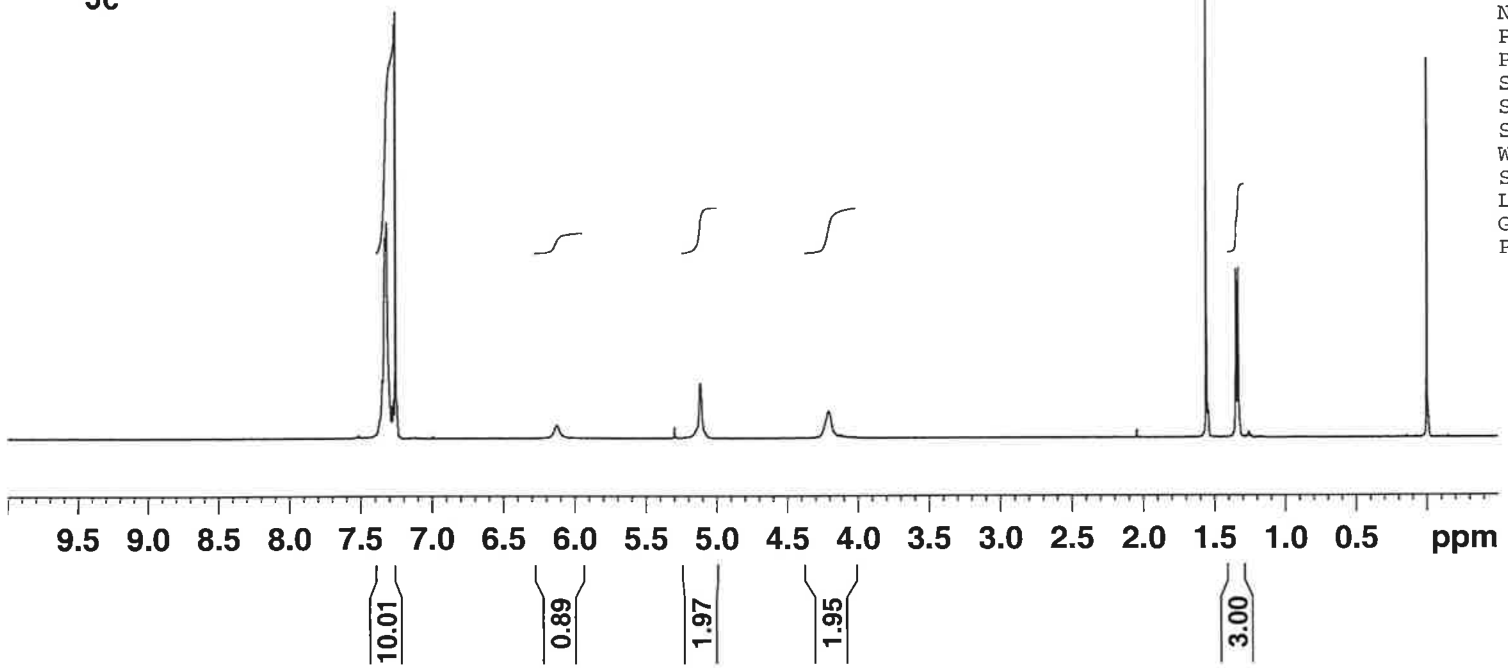

NAME	Aug20-2021-sys	
EXPNO	40	
PROCNO	1	
Date_	20210820	
Time	15.59	
INSTRUM	spect	
PROBHD	5 mm PABBO BB-	
PULPROG	zg30	
TD	65536	
SOLVENT	CDCl3	
NS	16	
DS	2	
SWH	8278.146	Hz
FIDRES	0.126314	Hz
AQ	3.9584243	sec
RG	362	
DW	60.400	usec
DE	6.50	usec
TE	297.6	K
D1	1.00000000	sec
TD0	1	
======== CHANNEL f1 ========		
NUC1	1H	
P1	11.60	usec
PL1	3.00	dB
SFO1	400.1324710	MHz
SI	32768	
SF	400.1300099	MHz
WDW	EM	
SSB	0	
LB	0.30	Hz
GB	0	
PC	1.00	

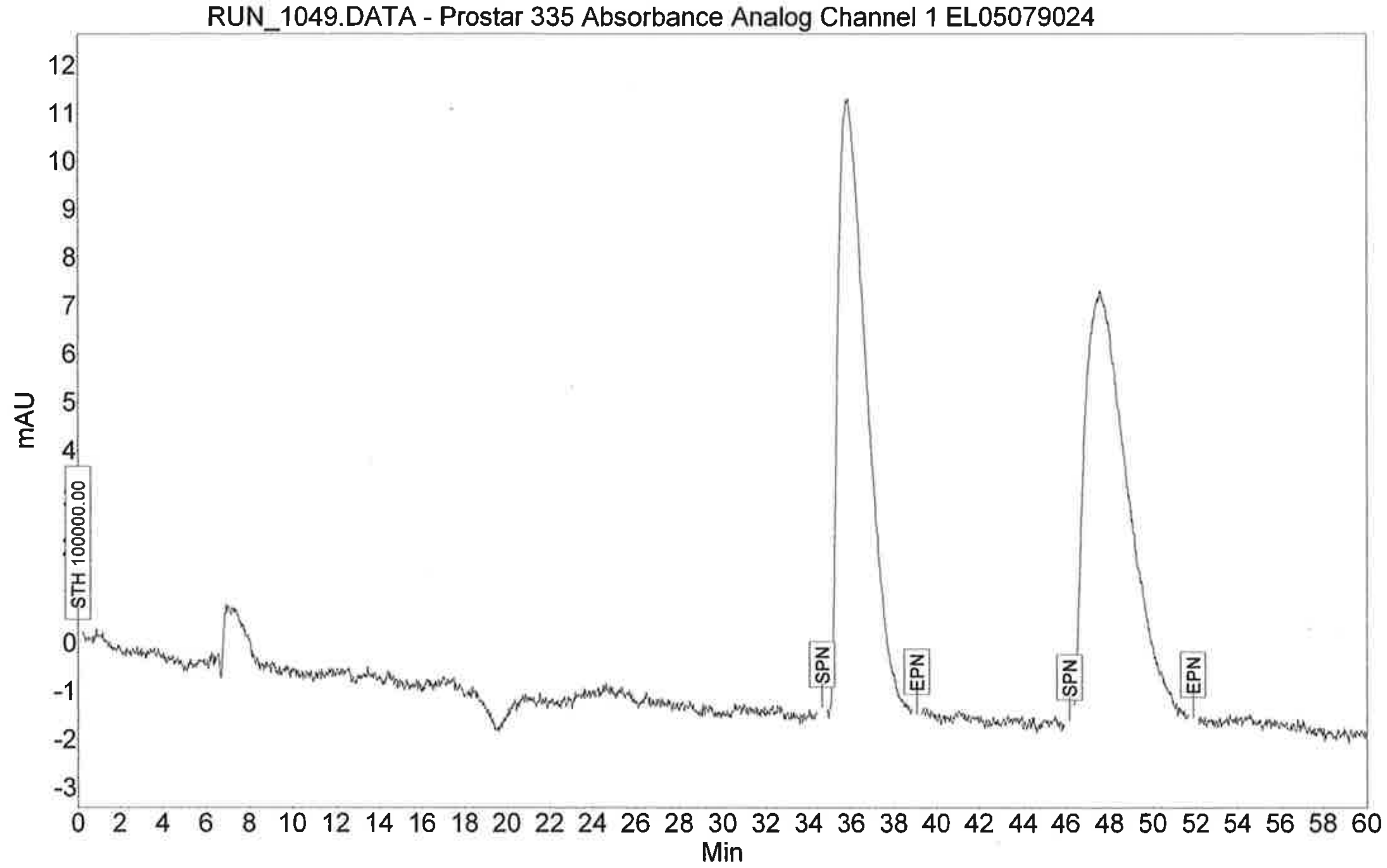

Index	Name	Time [Min]	Quantity [% Area]	Height [mAU]	Area [mAU.Min]	Area % [%]
1	UNKNOWN	35.77	49.24	12.7	19.9	49.243
2	UNKNOWN	47.57	50.76	9.0	20.5	50.757
Total			100.00	21.6	40.5	100.000

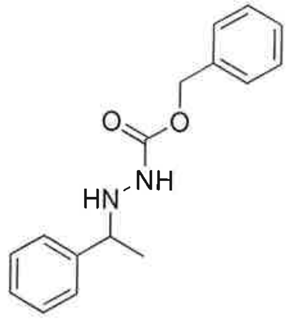

(±)

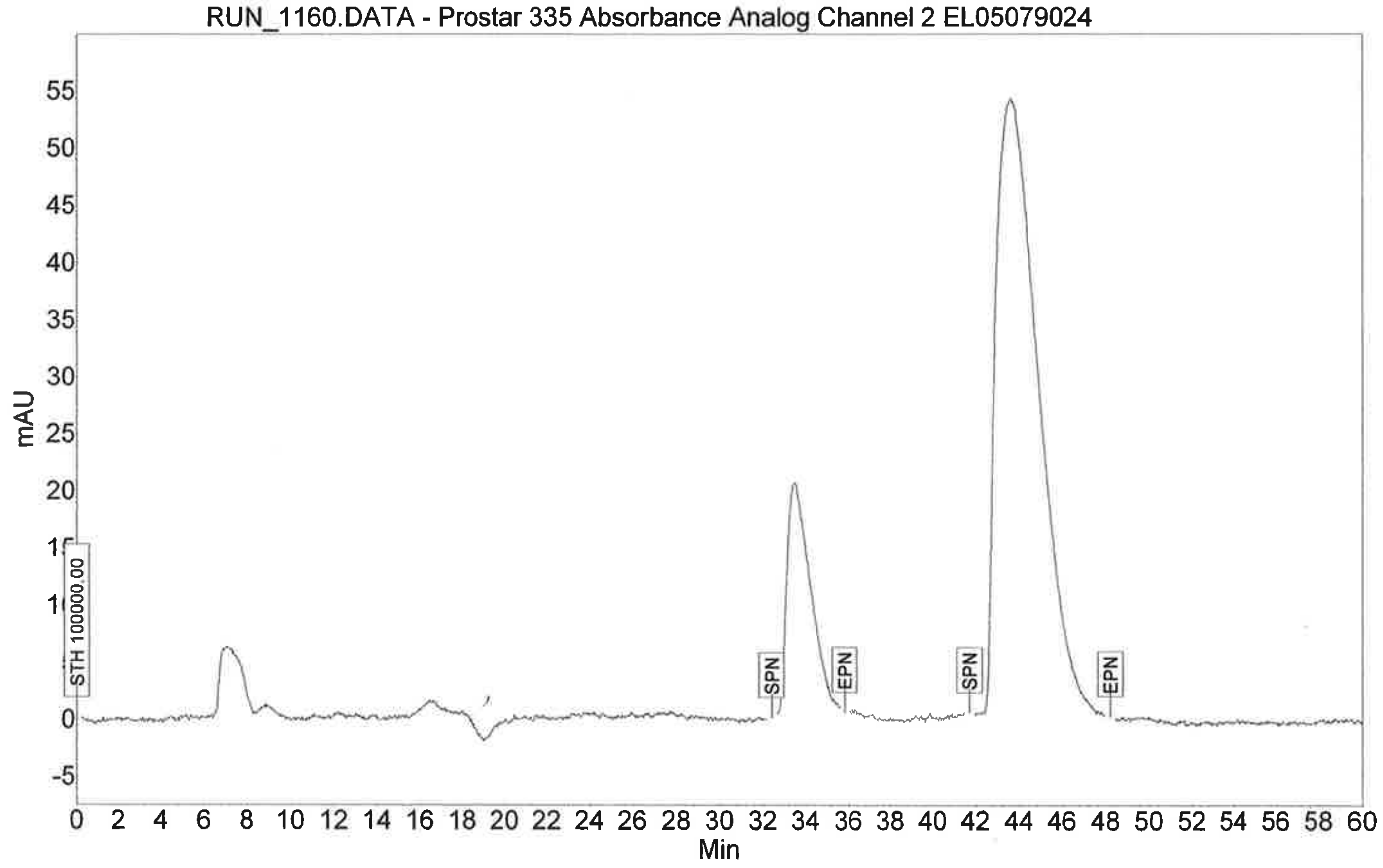

Index	Name	Time [Min]	Quantity [% Area]	Height [mAU]	Area [mAU.Min]	Area % [%]
1	UNKNOWN	33.45	17.87	20.4	26.6	17.867
2	UNKNOWN	43.57	82.13	53.9	122.2	82.133
Total			100.00	74.3	148.8	100.000

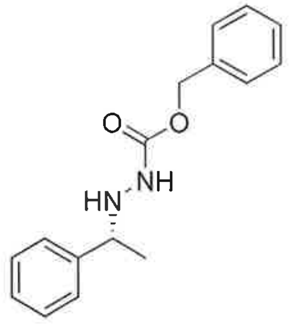

3c

NO2

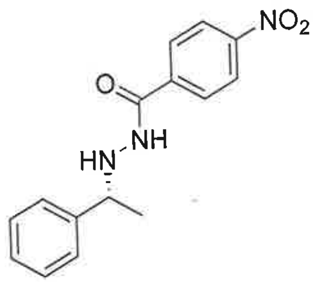

3d

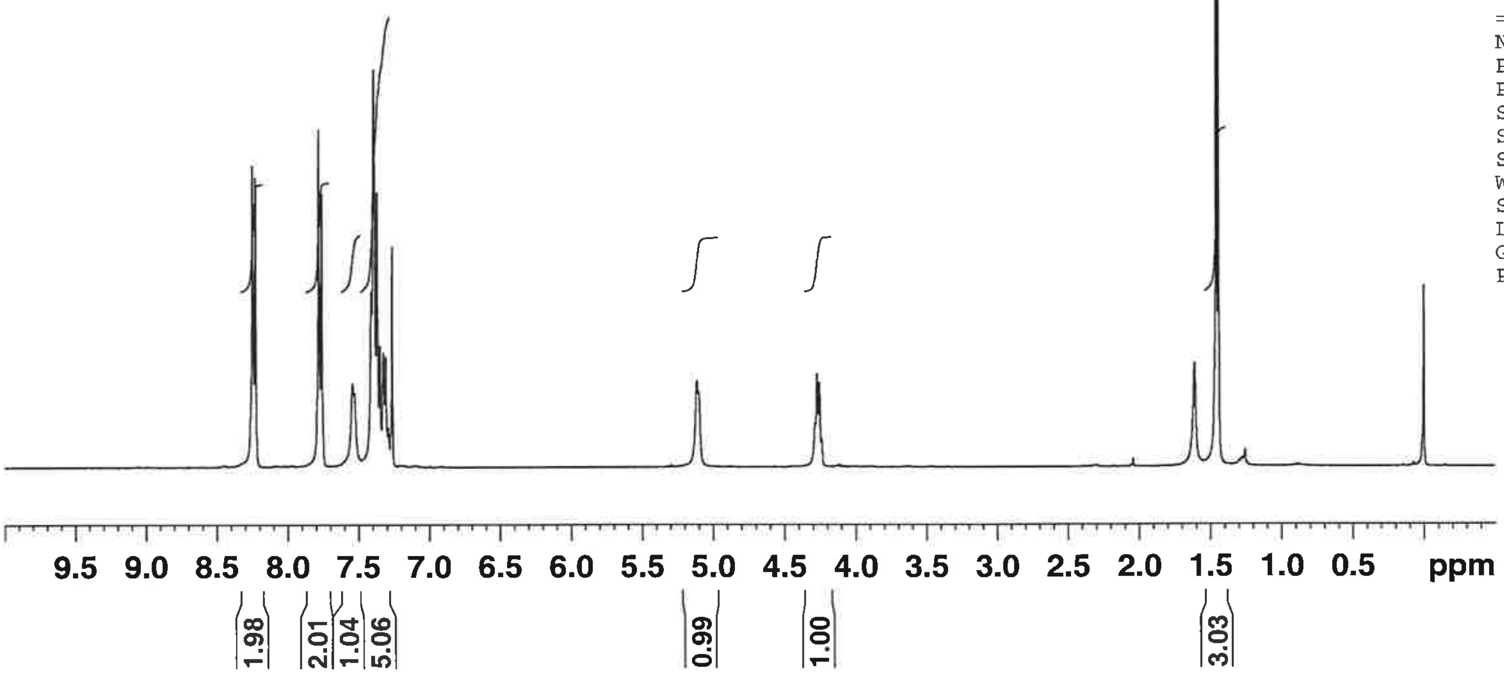

NAME	Aug15-2021-sys
EXPNO	30
PROCNO	1
Date_	20210815
Time	16.19
INSTRUM	spect
PROBHD	5 mm PABBO BB-
PULPROG	zg30
TD	65536
SOLVENT	CDCl3
NS	16
DS	2
SWH	8278.146 Hz
FIDRES	0.126314 Hz
AQ	3.9584243 sec
RG	256
DW	60.400 usec
DE	6.50 usec
TE	296.3 K
D1	1.00000000 sec
TD0	1

======== CHANNEL f1 ========

NUC1	1H
P1	11.60 usec
PL1	3.00 dB
SFO1	400.1324710 MHz
SI	32768
SF	400.1300083 MHz
WDW	EM
SSB	0
LB	0.30 Hz
GB	0
PC	1.00

NO2

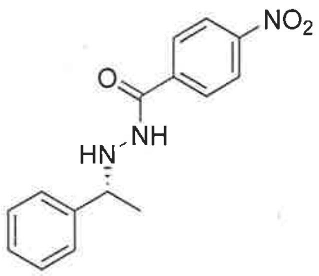

3d

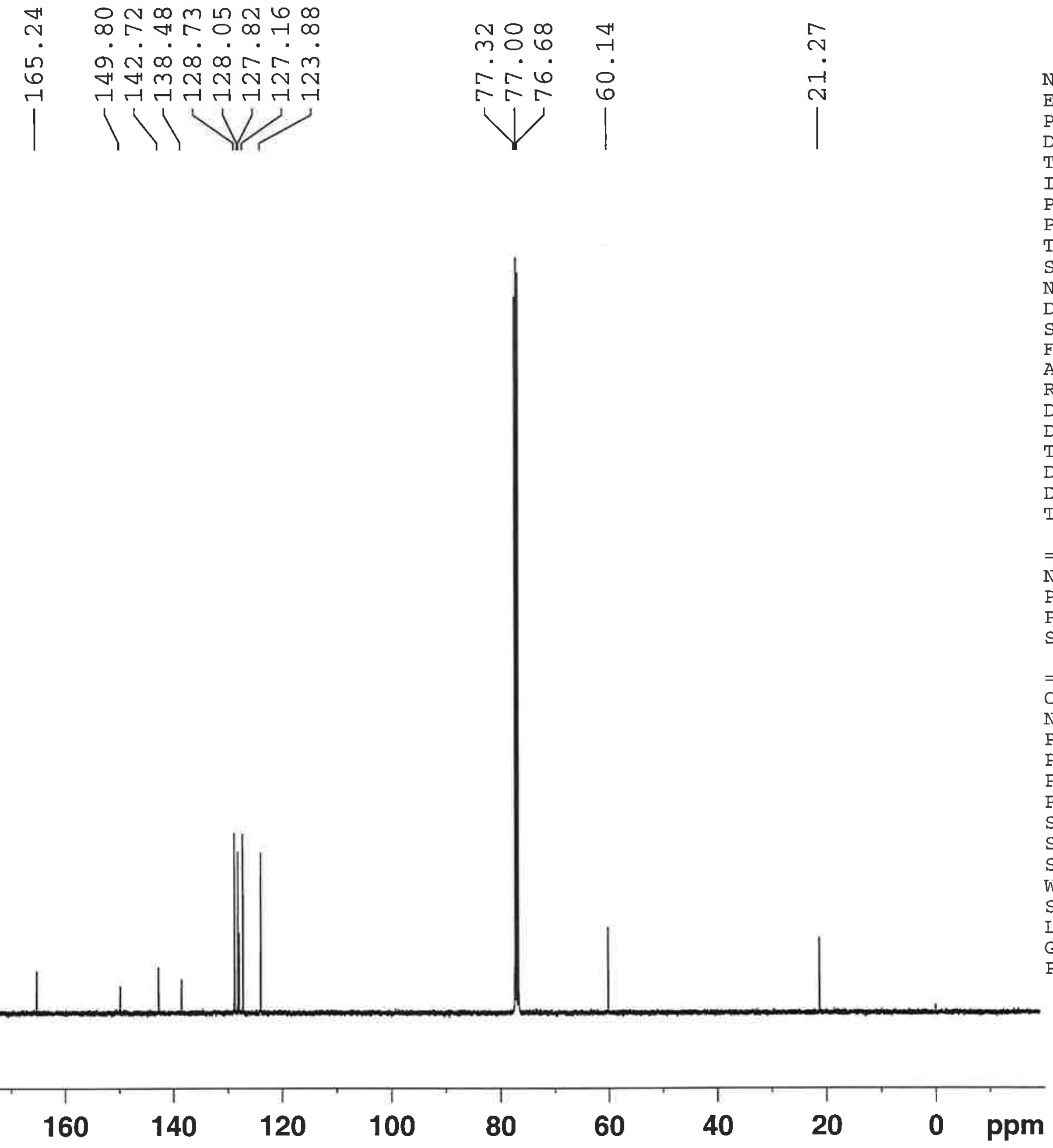

NAME	Aug16-2021-sys	
EXPNO	71	
PROCNO	1	
Date_	20210817	
Time	7.59	
INSTRUM	spect	
PROBHD	5 mm PABBO BB-	
PULPROG	zgpg30	
TD	65536	
SOLVENT	CDCl3	
NS	10024	
DS	4	
SWH	23980.814	Hz
FIDRES	0.365918	Hz
AQ	1.3664756	sec
RG	32768	
DW	20.850	usec
DE	6.50	usec
TE	301.6	K
D1	2.00000000	sec
D11	0.03000000	sec
TD0	1	
========	CHANNEL f1	========
NUC1	13C	
P1	14.75	usec
PL1	0.00	dB
SFO1	100.6228298	MHz
========	CHANNEL f2	========
CPDPRG2	waltz16	
NUC2	1H	
PCPD2	80.00	usec
PL2	-2.00	dB
PL12	12.54	dB
PL13	15.00	dB
SFO2	400.1316005	MHz
SI	32768	
SF	100.6127688	MHz
WDW	EM	
SSB	0	
LB	1.00	Hz
GB	0	
PC	1.40	

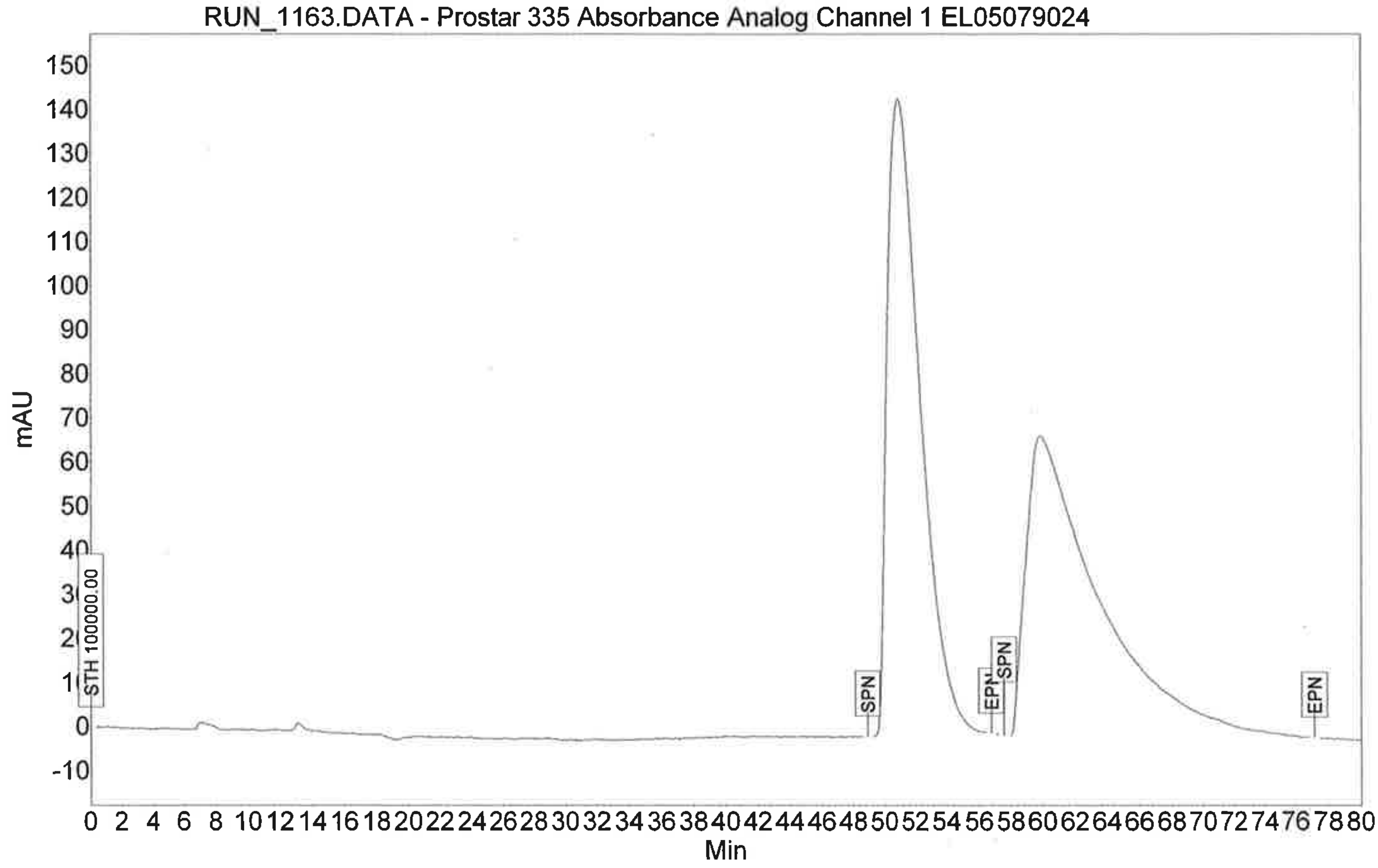

Index	Name	Time [Min]	Quantity [% Area]	Height [mAU]	Area [mAU.Min]	Area % [%]
1	UNKNOWN	50.85	50.34	144.5	361.9	50.341
2	UNKNOWN	59.80	49.66	68.0	357.0	49.659
Total			100.00	212.6	718.9	100.000

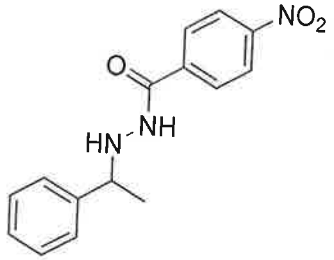

(±)

RUN_1165.DATA - Prostar 335 Absorbance Analog Channel 1 EL05079024

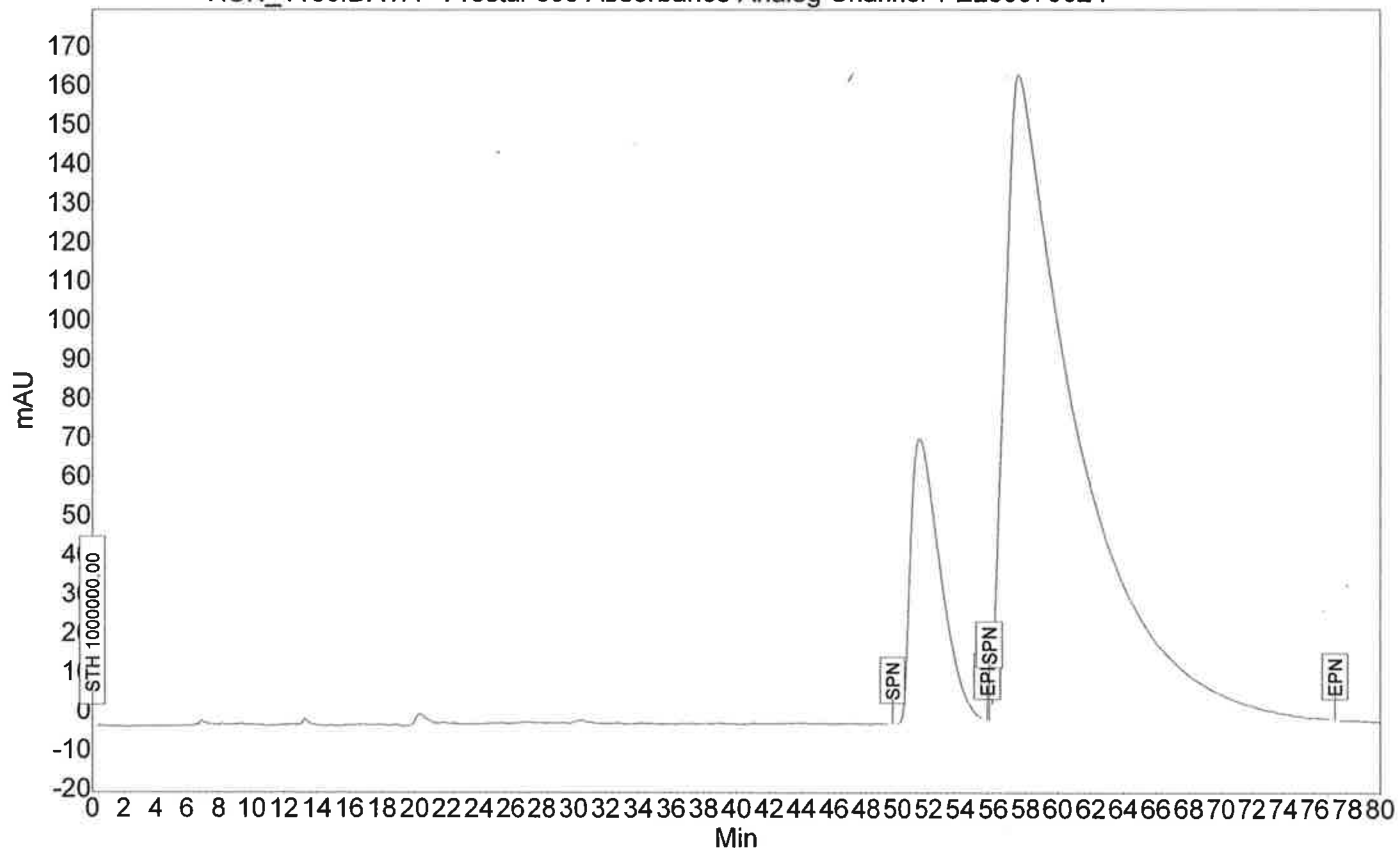

Index	Name	Time [Min]	Quantity [% Area]	Height [mAU]	Area [mAU.Min]	Area % [%]
1	UNKNOWN	51.44	15.72	72.6	159.7	15.720
2	UNKNOWN	57.56	84.28	165.1	856.2	84.280
Total			100.00	237.6	1015.8	100.000

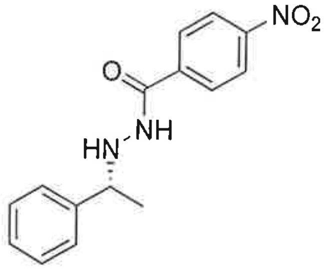

3d

Me

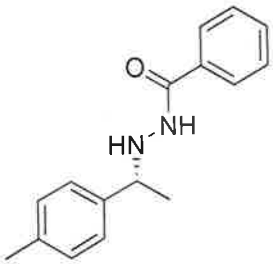

3e

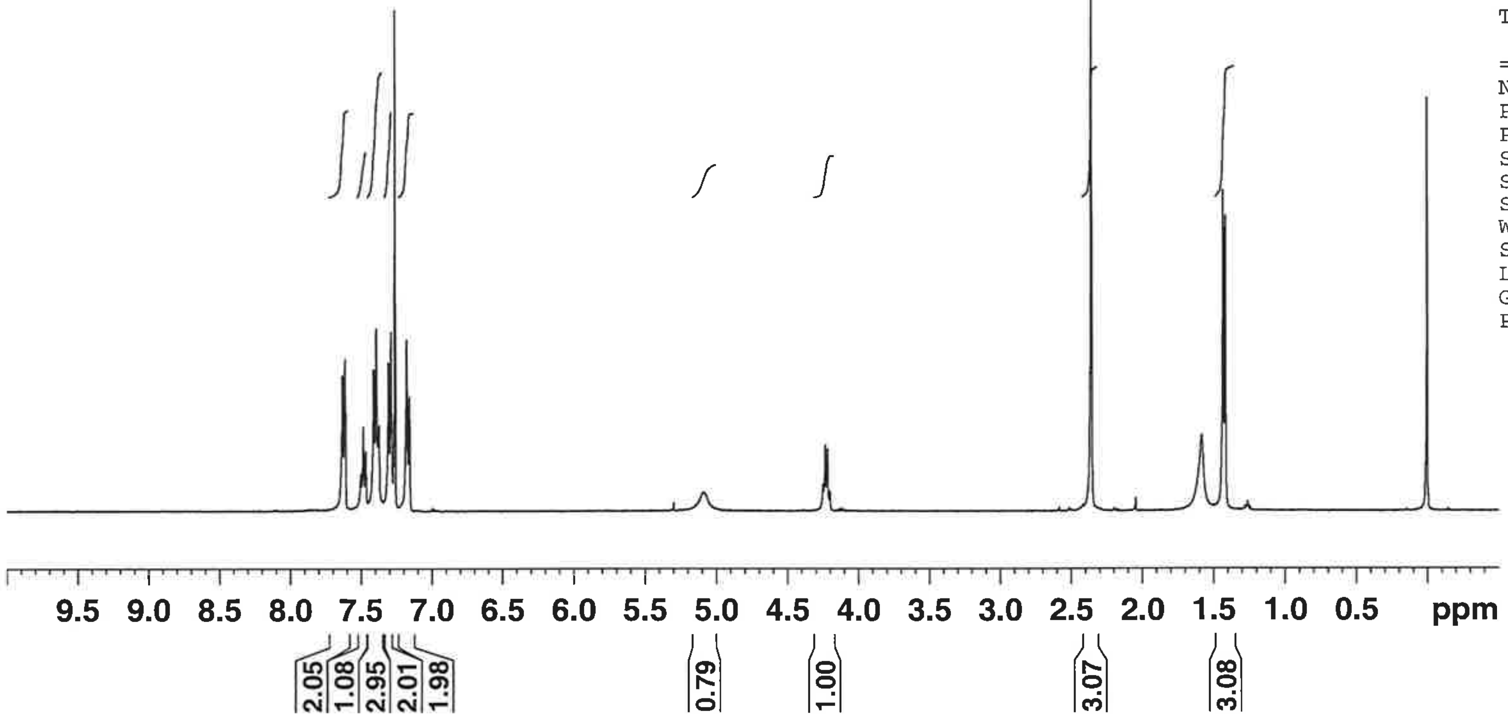

NAME	Aug14-2021-sys	
EXPNO	60	
PROCNO	1	
Date_	20210814	
Time	16.31	
INSTRUM	spect	
PROBHD	5 mm PABBO BB-	
PULPROG	zg30	
TD	65536	
SOLVENT	CDCl3	
NS	16	
DS	2	
SWH	8278.146	Hz
FIDRES	0.126314	Hz
AQ	3.9584243	sec
RG	362	
DW	60.400	usec
DE	6.50	usec
TE	296.2	K
D1	1.00000000	sec
TD0	1	

======== CHANNEL f1 ========

NUC1	1H	
P1	11.60	usec
PL1	3.00	dB
SFO1	400.1324710	MHz
SI	32768	
SF	400.1300089	MHz
WDW	EM	
SSB	0	
LB	0.30	Hz
GB	0	
PC	1.00	

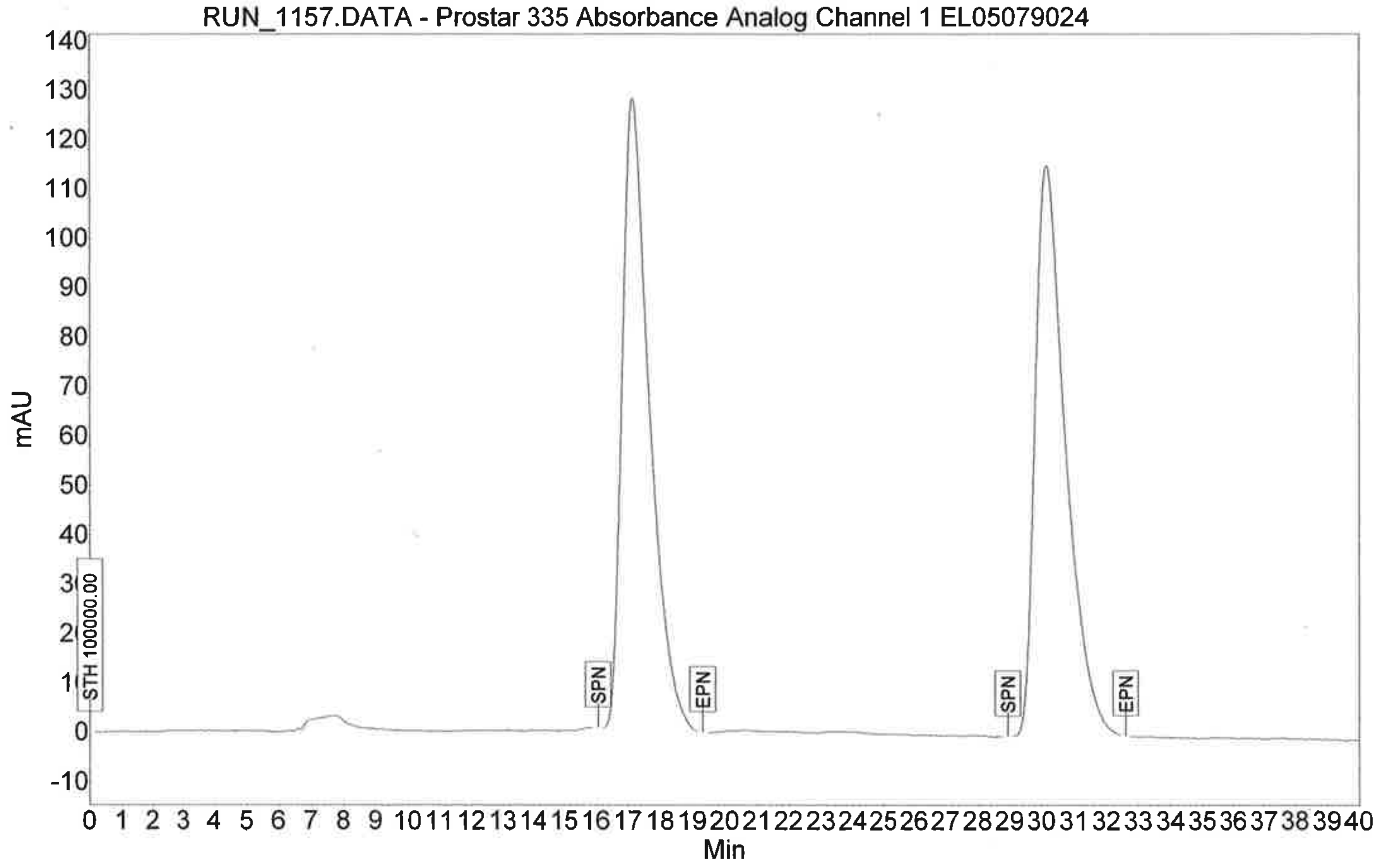

Index	Name	Time [Min]	Quantity [% Area]	Height [mAU]	Area [mAU.Min]	Area % [%]
1	UNKNOWN	17.15	49.95	127.7	126.4	49.947
2	UNKNOWN	30.15	50.05	115.5	126.7	50.053
Total			100.00	243.2	253.1	100.000

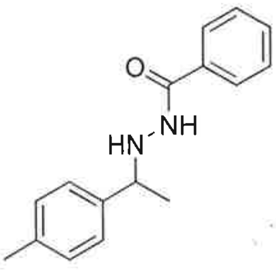

(±)

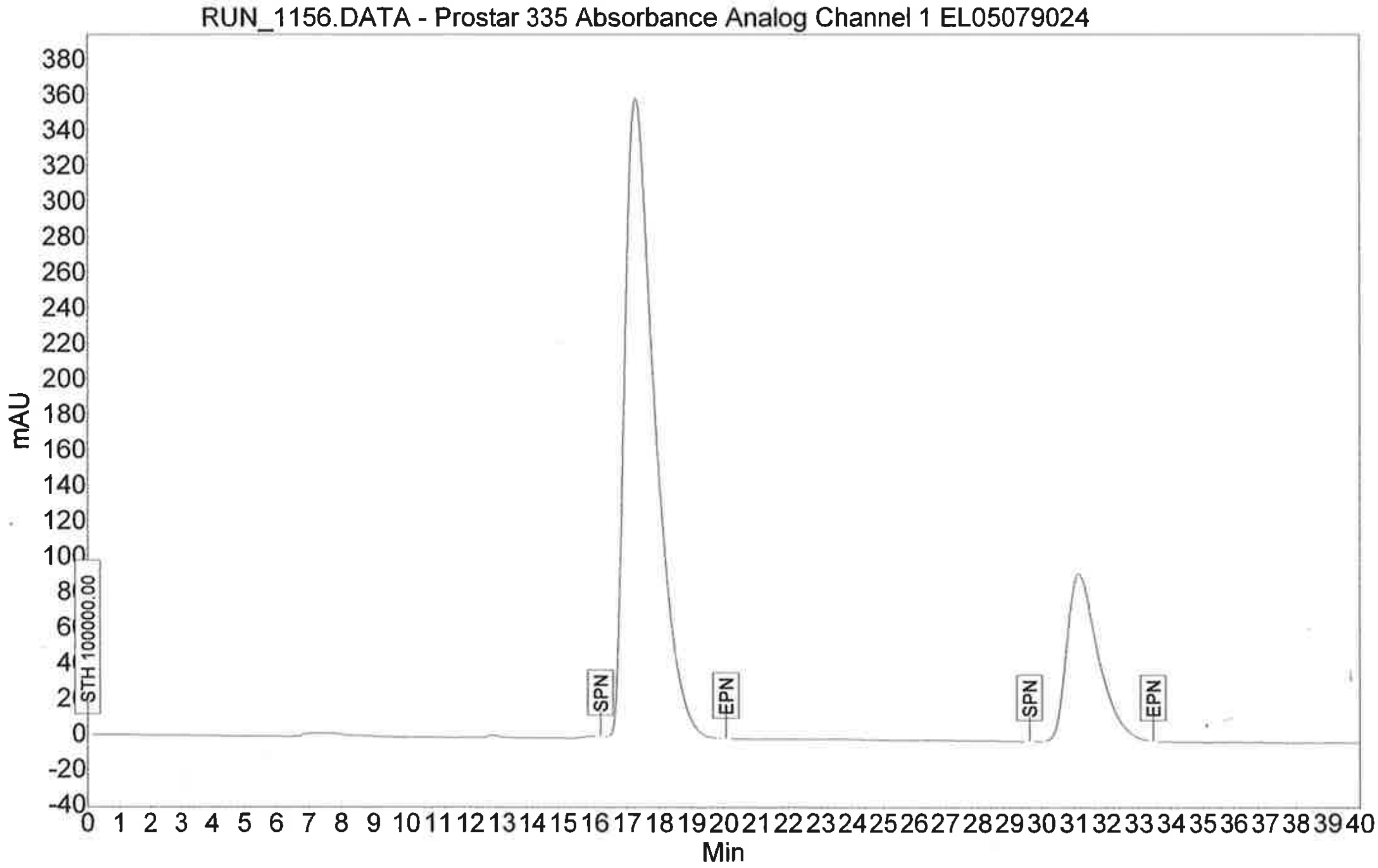

Index	Name	Time [Min]	Quantity [% Area]	Height [mAU]	Area [mAU.Min]	Area % [%]
1	UNKNOWN	17.27	78.64	359.4	388.6	78.642
2	UNKNOWN	31.13	21.36	94.1	105.5	21.358
Total			100.00	453.5	494.2	100.000

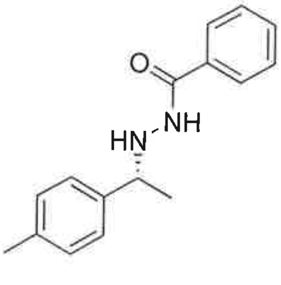

3e

3Me

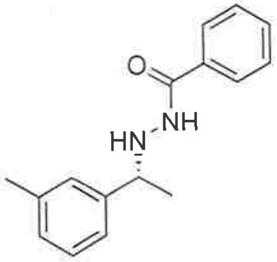

3f

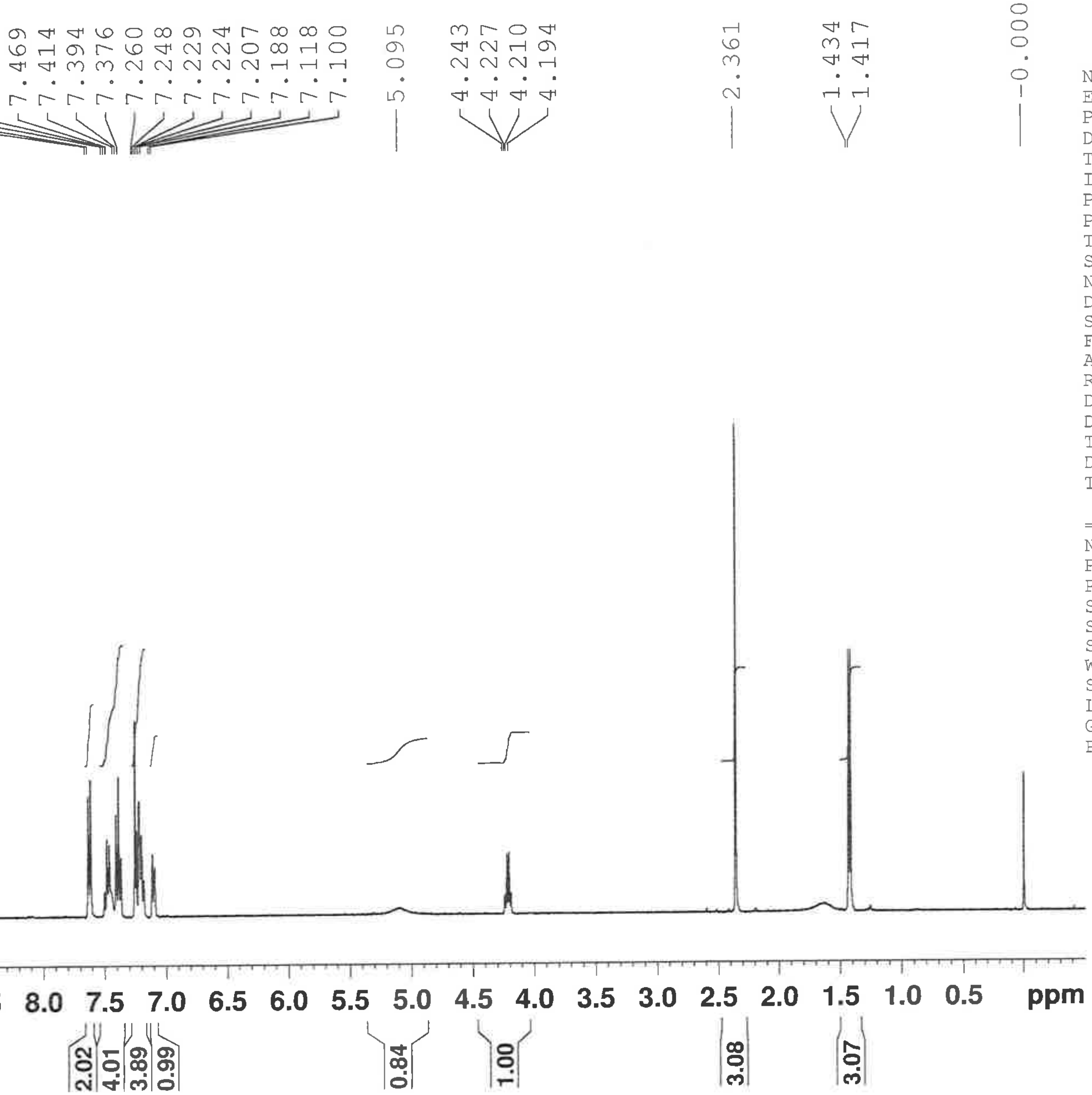

NAME	Aug13-2021-cxu
EXPNO	1
PROCNO	1
Date_	20210813
Time	12.26
INSTRUM	spect
PROBHD	5 mm TBI 1H/31
PULPROG	zg30
TD	65536
SOLVENT	CDCl3
NS	16
DS	2
SWH	8278.146 Hz
FIDRES	0.126314 Hz
AQ	3.9584243 sec
RG	13004
DW	60.400 usec
DE	6.50 usec
TE	300.0 K
D1	1.00000000 sec
TD0	1
======== CHANNEL f1 ========	
NUC1	1H
P1	18.75 usec
PL1	0.50 dB
SFO1	400.1324710 MHz
SI	32768
SF	400.1300095 MHz
WDW	EM
SSB	0
LB	0.30 Hz
GB	0
PC	1.00

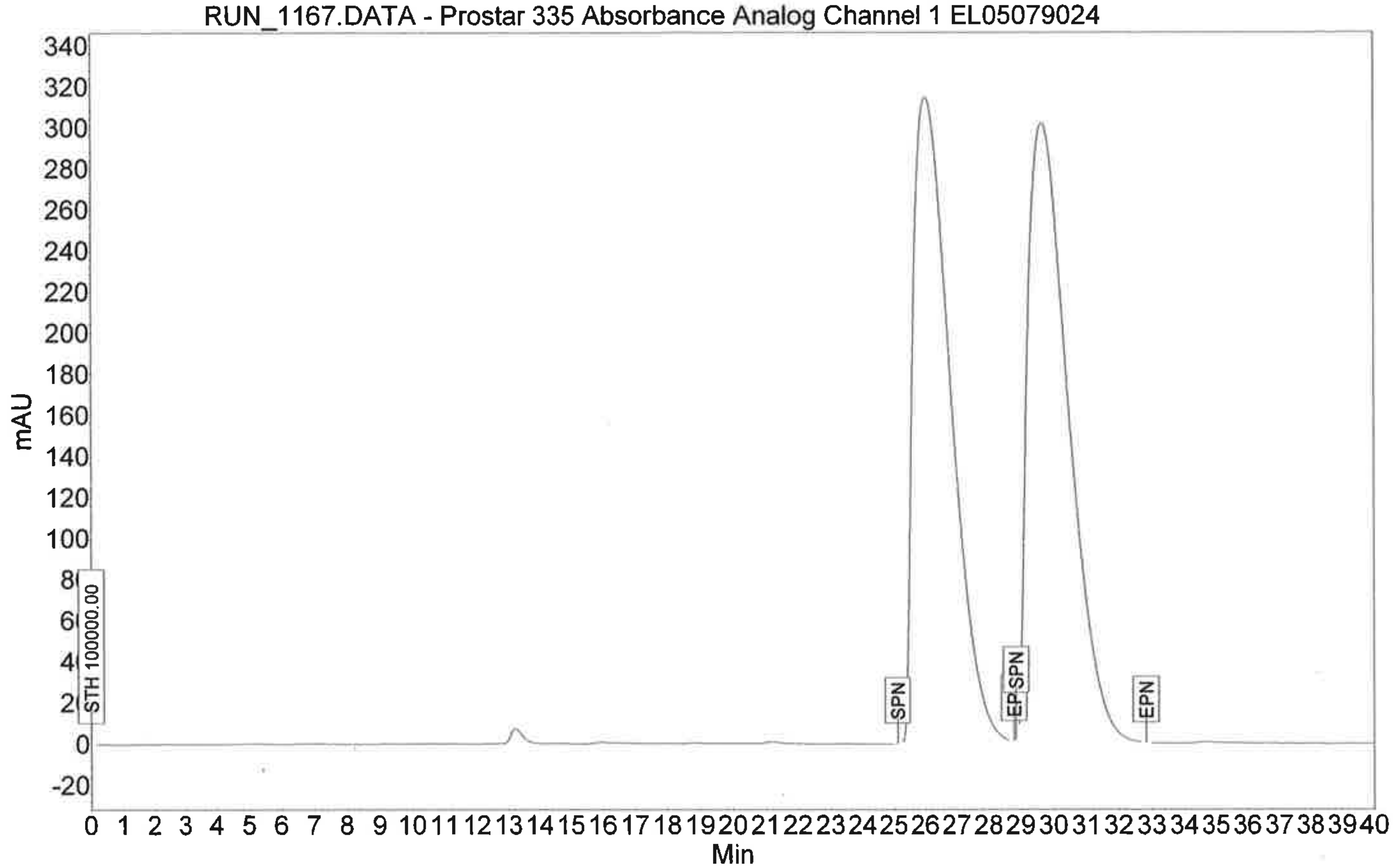

Index	Name	Time [Min]	Quantity [% Area]	Height [mAU]	Area [mAU.Min]	Area % [%]
1	UNKNOWN	26.03	49.93	314.4	432.1	49.932
2	UNKNOWN	29.65	50.07	300.9	433.3	50.068
Total			100.00	615.3	865.5	100.000

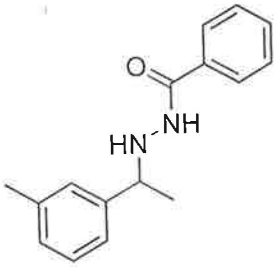

(±)

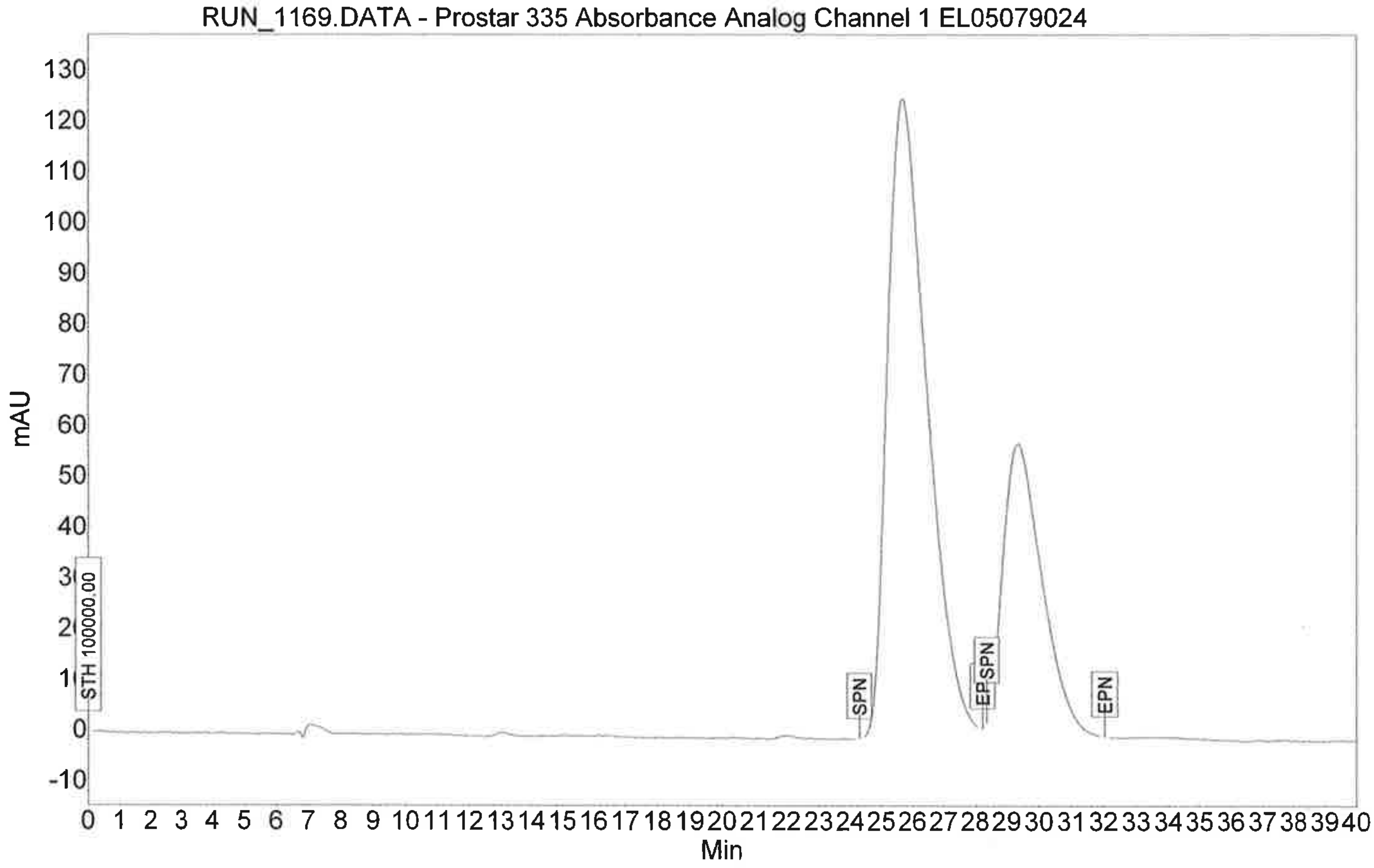

Index	Name	Time [Min]	Quantity [% Area]	Height [mAU]	Area [mAU.Min]	Area % [%]
1	UNKNOWN	25.65	70.37	125.4	184.4	70.373
2	UNKNOWN	29.36	29.63	56.2	77.6	29.627
Total			100.00	181.6	262.0	100.000

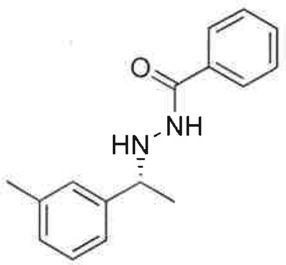

3f

MeO

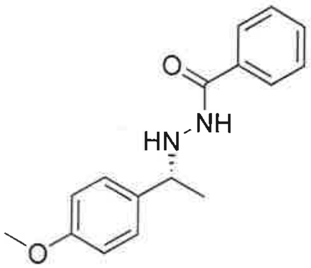

3h

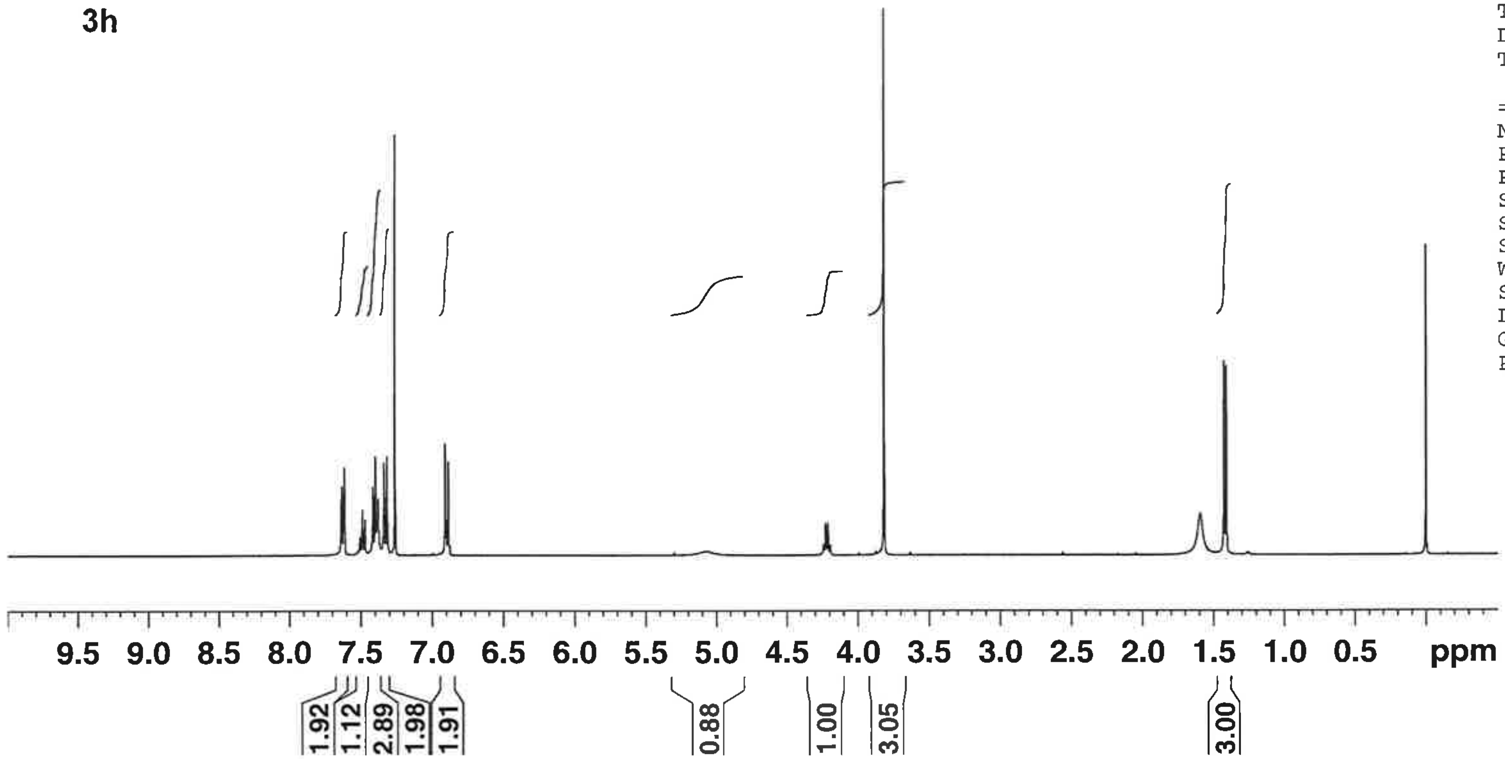

NAME	Aug14-2021-sys
EXPNO	50
PROCNO	1
Date_	20210814
Time	16.21
INSTRUM	spect
PROBHD	5 mm PABBO BB-
PULPROG	zg30
TD	65536
SOLVENT	CDCl3
NS	16
DS	2
SWH	8278.146 Hz
FIDRES	0.126314 Hz
AQ	3.9584243 sec
RG	362
DW	60.400 usec
DE	6.50 usec
TE	296.2 K
D1	1.00000000 sec
TD0	1

======== CHANNEL f1 ========

NUC1	1H
P1	11.60 usec
PL1	3.00 dB
SFO1	400.1324710 MHz
SI	32768
SF	400.1300089 MHz
WDW	EM
SSB	0
LB	0.30 Hz
GB	0
PC	1.00

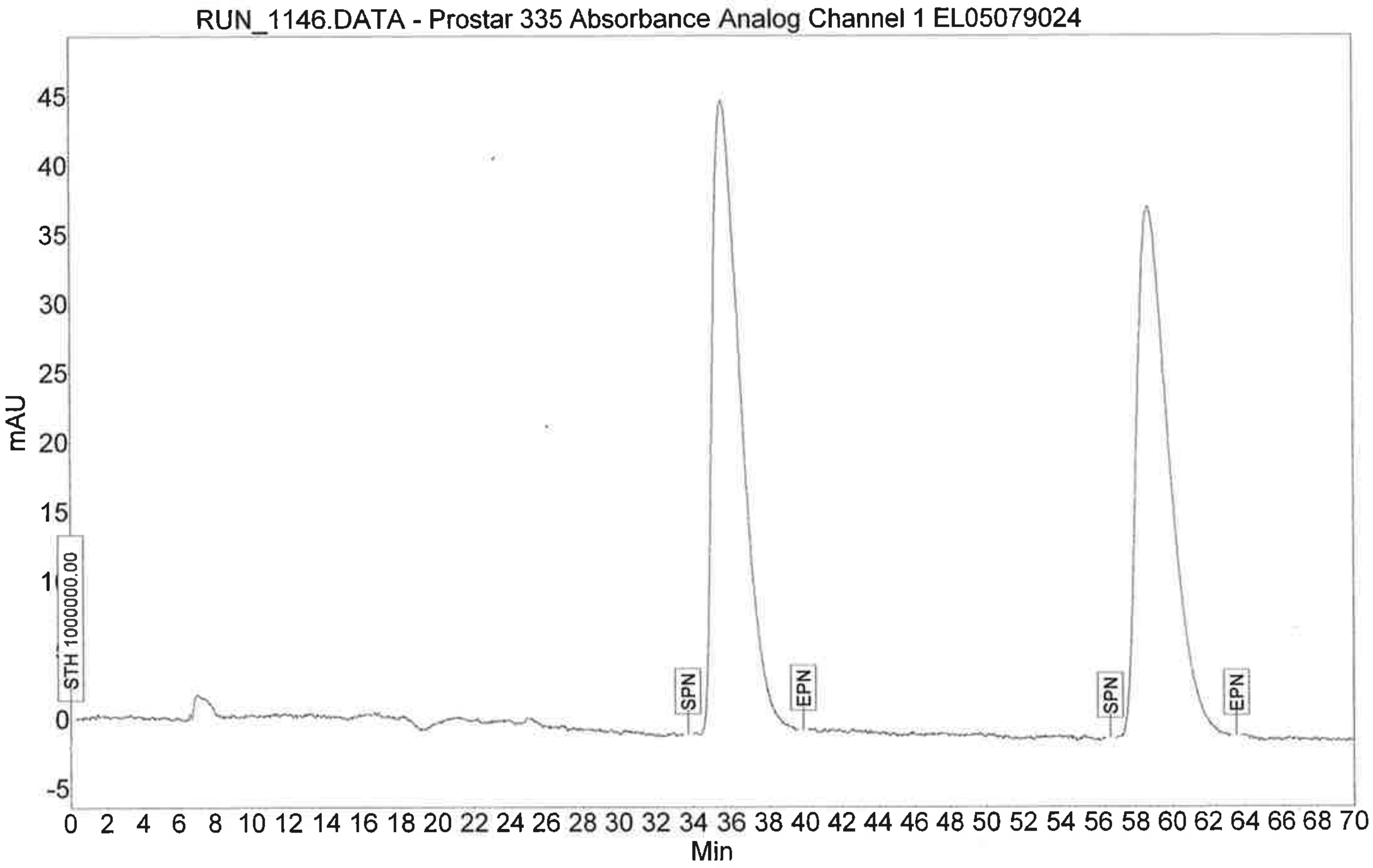

Index	Name	Time [Min]	Quantity [% Area]	Height [mAU]	Area [mAU.Min]	Area % [%]
1	UNKNOWN	35.53	50.13	45.7	79.2	50.135
2	UNKNOWN	58.68	49.87	38.4	78.7	49.865
Total			100.00	84.2	157.9	100.000

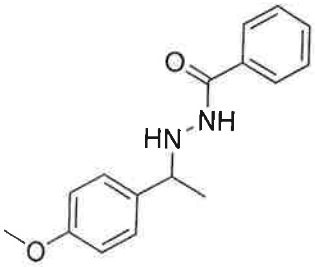

(±)

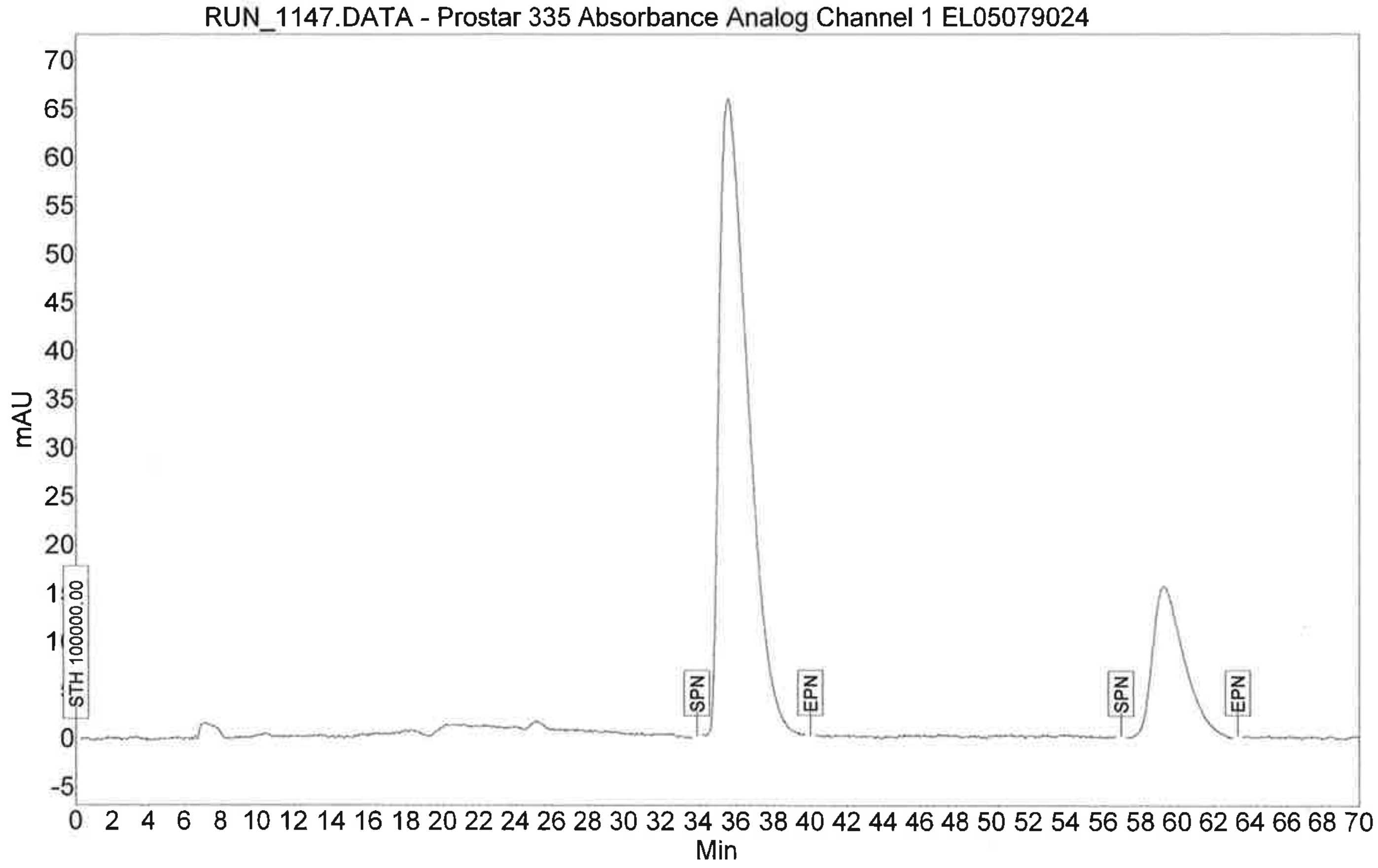

Index	Name	Time [Min]	Quantity [% Area]	Height [mAU]	Area [mAU.Min]	Area % [%]
1	UNKNOWN	35.59	79.13	65.8	115.7	79.133
2	UNKNOWN	59.24	20.87	15.4	30.5	20.867
Total			100.00	81.2	146.2	100.000

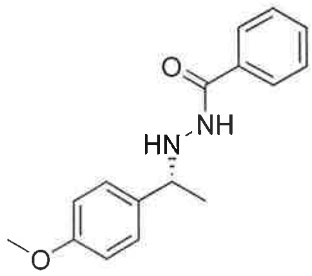

3h

F

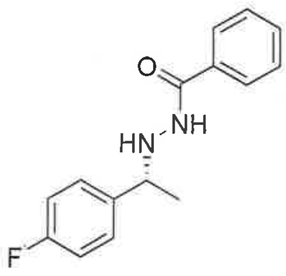

3i

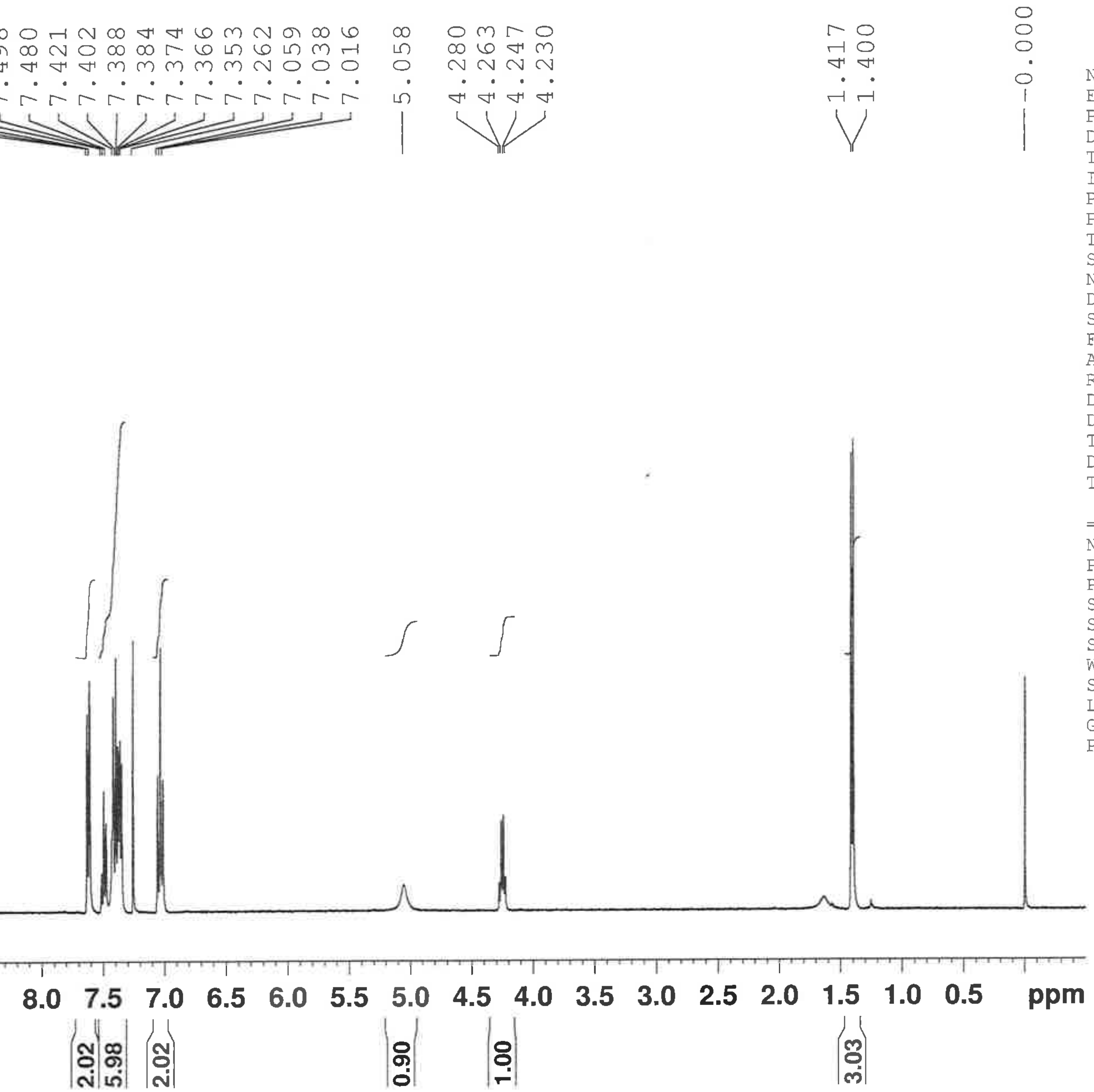

NAME	Aug11-2021
EXPNO	10
PROCNO	1
Date_	20210811
Time	17.23
INSTRUM	spect
PROBHD	5 mm TBI 1H/31
PULPROG	zg30
TD	65536
SOLVENT	CDCl3
NS	16
DS	2
SWH	8278.146 Hz
FIDRES	0.126314 Hz
AQ	3.9584243 sec
RG	13004
DW	60.400 usec
DE	6.50 usec
TE	300.0 K
D1	1.00000000 sec
TD0	1

======== CHANNEL f1 ========

NUC1	1H
P1	18.75 usec
PL1	0.50 dB
SFO1	400.1324710 MHz
SI	32768
SF	400.1300088 MHz
WDW	EM
SSB	0
LB	0.30 Hz
GB	0
PC	1.00

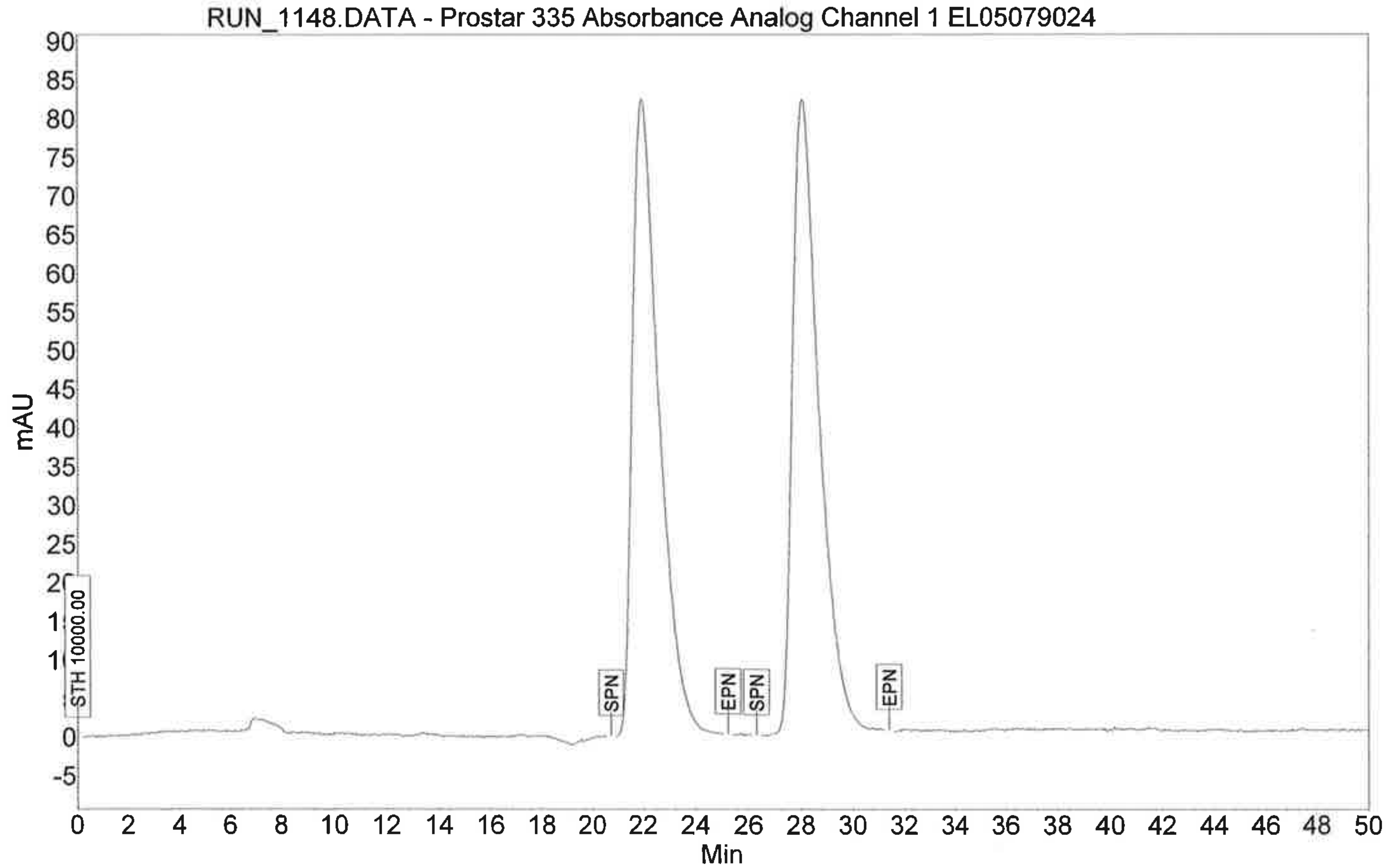

Index	Name	Time [Min]	Quantity [% Area]	Height [mAU]	Area [mAU.Min]	Area % [%]
1	UNKNOWN	21.93	49.98	82.2	97.8	49.982
2	UNKNOWN	28.03	50.02	81.9	97.9	50.018
Total			100.00	164.1	195.7	100.000

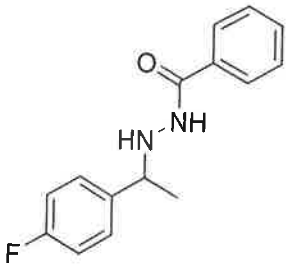

(±)

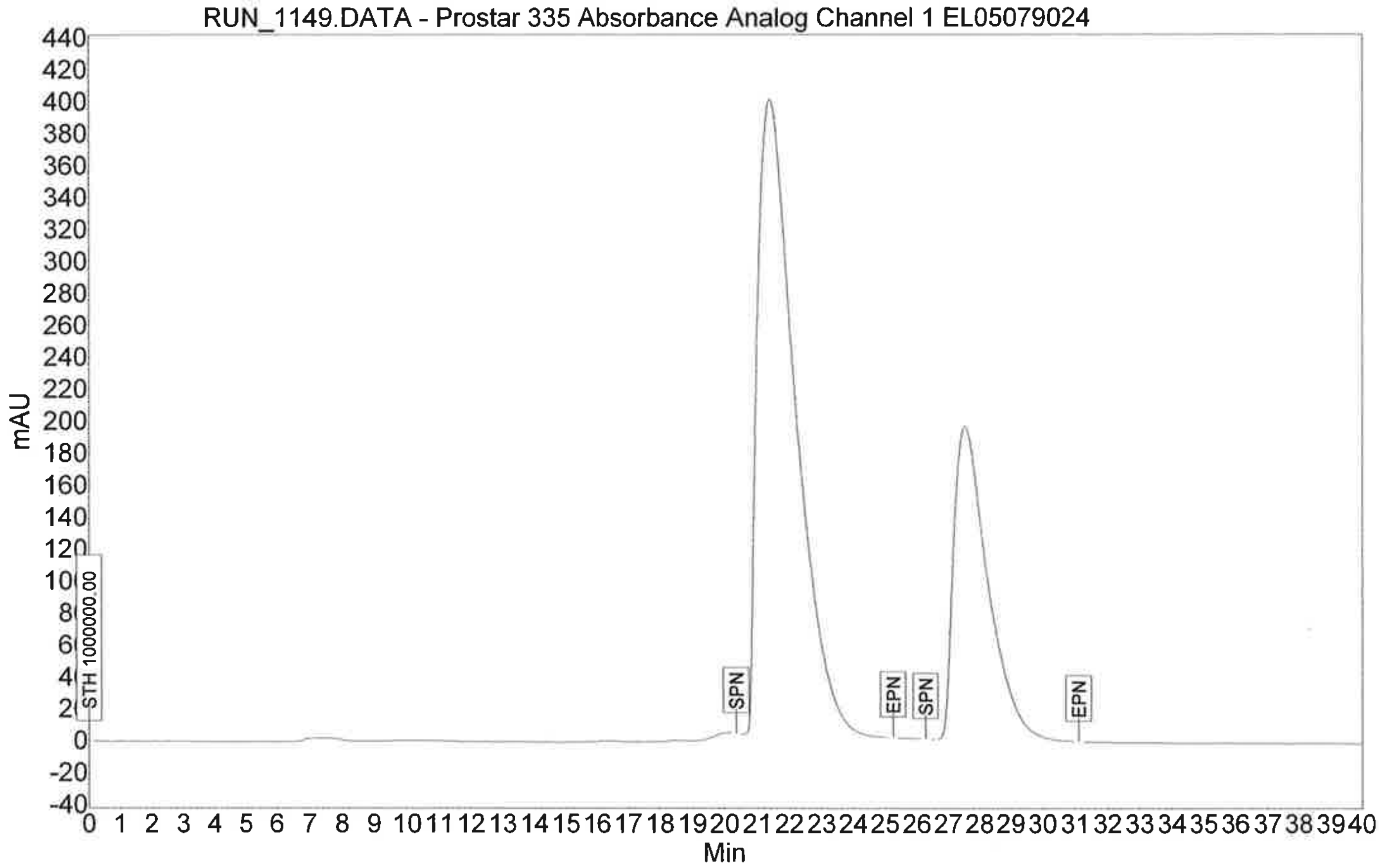

Index	Name	Time [Min]	Quantity [% Area]	Height [mAU]	Area [mAU.Min]	Area % [%]
1	UNKNOWN	21.37	68.67	397.3	545.0	68.674
2	UNKNOWN	27.53	31.33	196.0	248.6	31.326
Total			100.00	593.4	793.6	100.000

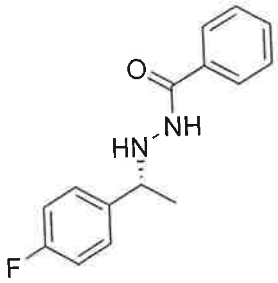

3i

C1

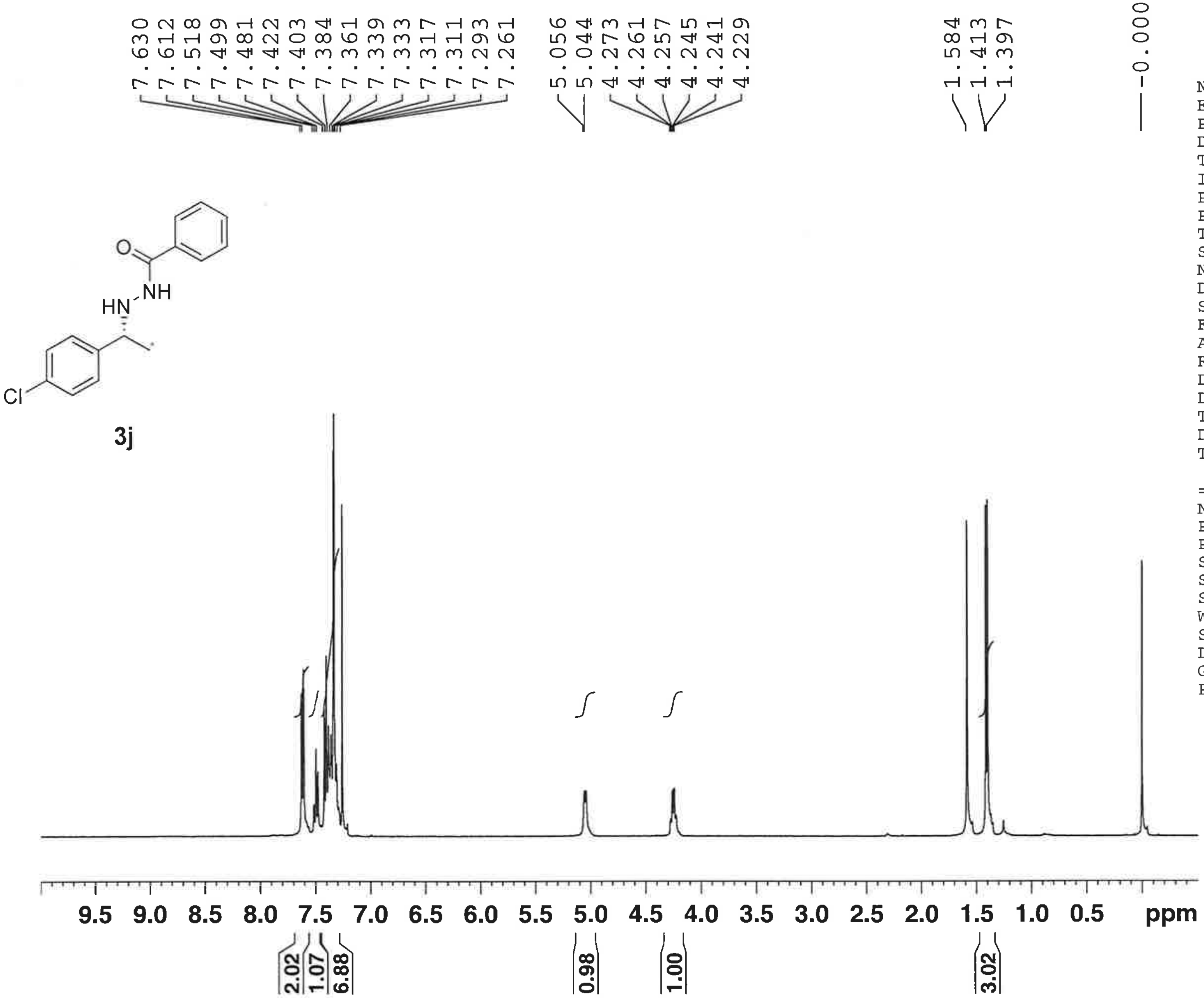

3j

NAME	Aug14-2021-sys
EXPNO	40
PROCNO	1
Date_	20210814
Time	16.09
INSTRUM	spect
PROBHD	5 mm PABBO BB-
PULPROG	zg30
TD	65536
SOLVENT	CDCl3
NS	16
DS	2
SWH	8278.146 Hz
FIDRES	0.126314 Hz
AQ	3.9584243 sec
RG	322.5
DW	60.400 usec
DE	6.50 usec
TE	296.1 K
D1	1.00000000 sec
TD0	1

======== CHANNEL f1 ========

NUC1	1H
P1	11.60 usec
PL1	3.00 dB
SFO1	400.1324710 MHz
SI	32768
SF	400.1300095 MHz
WDW	EM
SSB	0
LB	0.30 Hz
GB	0
PC	1.00

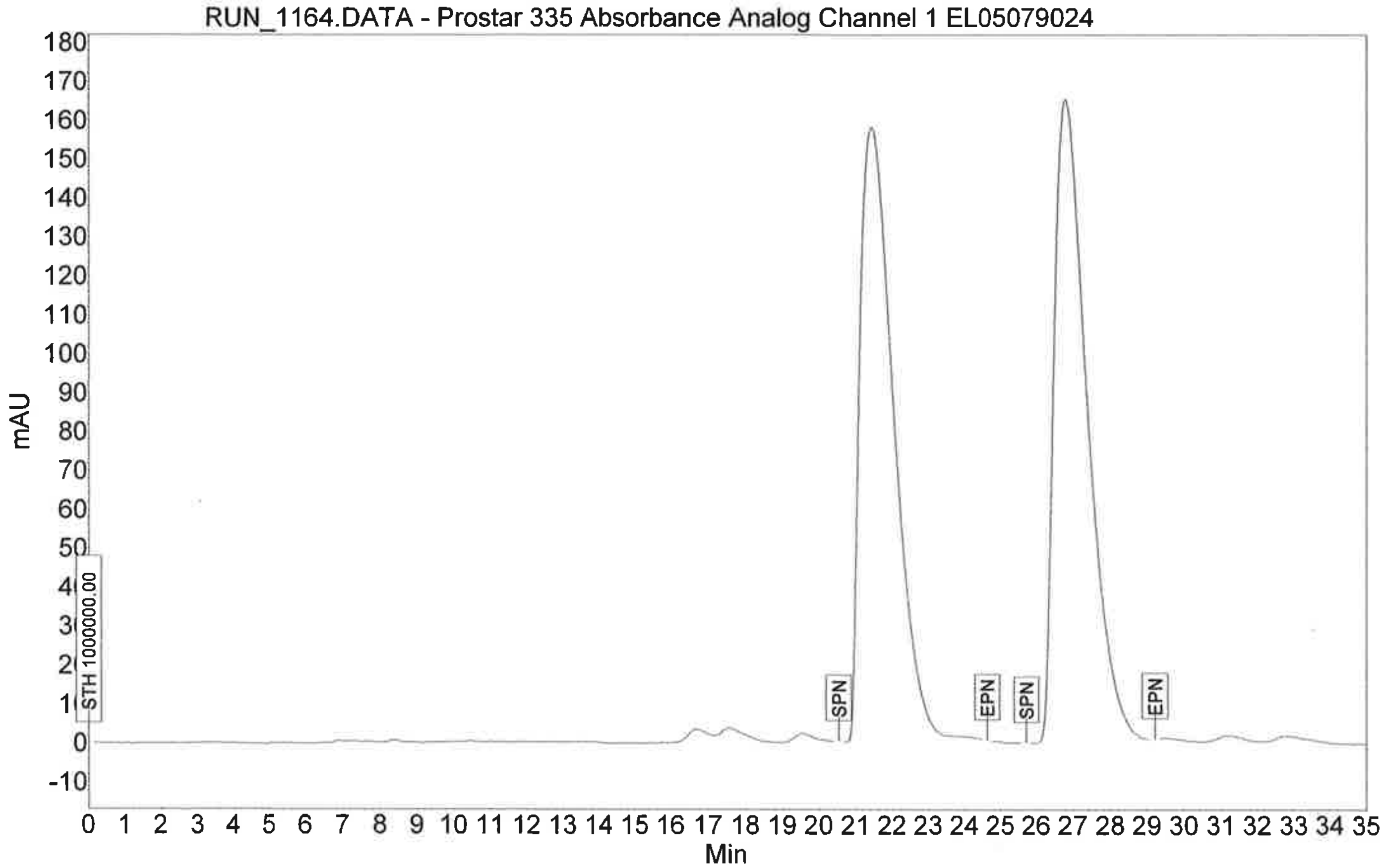

Index	Name	Time [Min]	Quantity [% Area]	Height [mAU]	Area [mAU.Min]	Area % [%]
1	UNKNOWN	21.43	49.98	157.6	169.2	49.977
2	UNKNOWN	26.77	50.02	165.0	169.3	50.023
Total			100.00	322.6	338.5	100.000

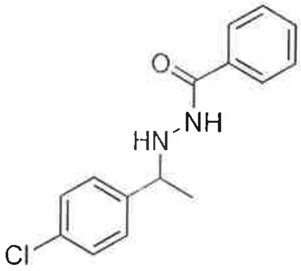

(±)

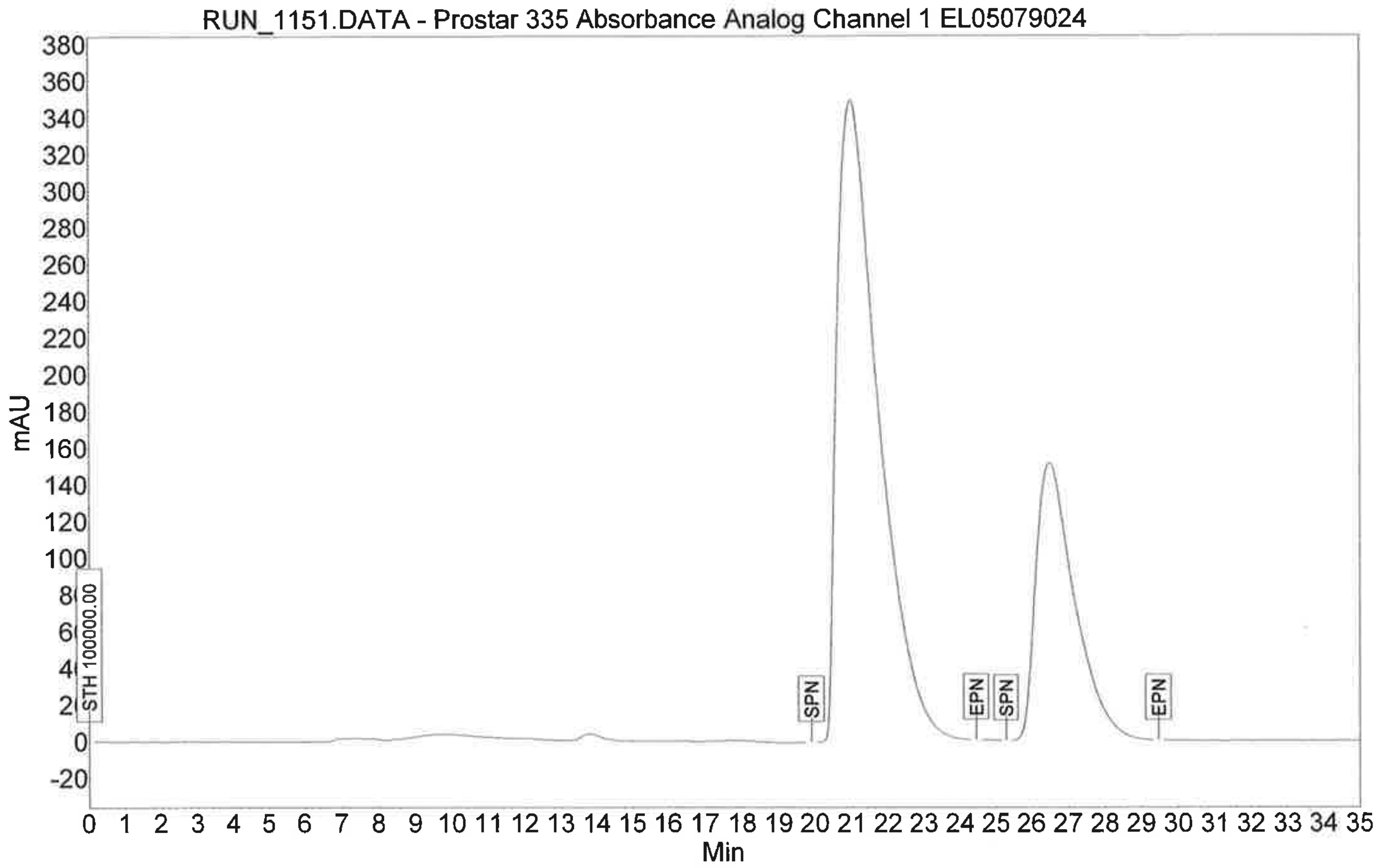

Index	Name	Time [Min]	Quantity [% Area]	Height [mAU]	Area [mAU.Min]	Area % [%]
1	UNKNOWN	20.97	71.50	349.3	459.2	71.503
2	UNKNOWN	26.49	28.50	151.1	183.0	28.497
Total			100.00	500.4	642.2	100.000

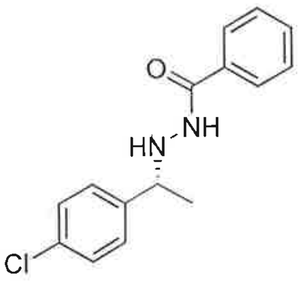

3j

Br

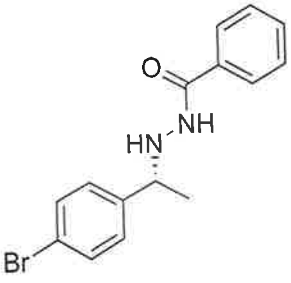

3k

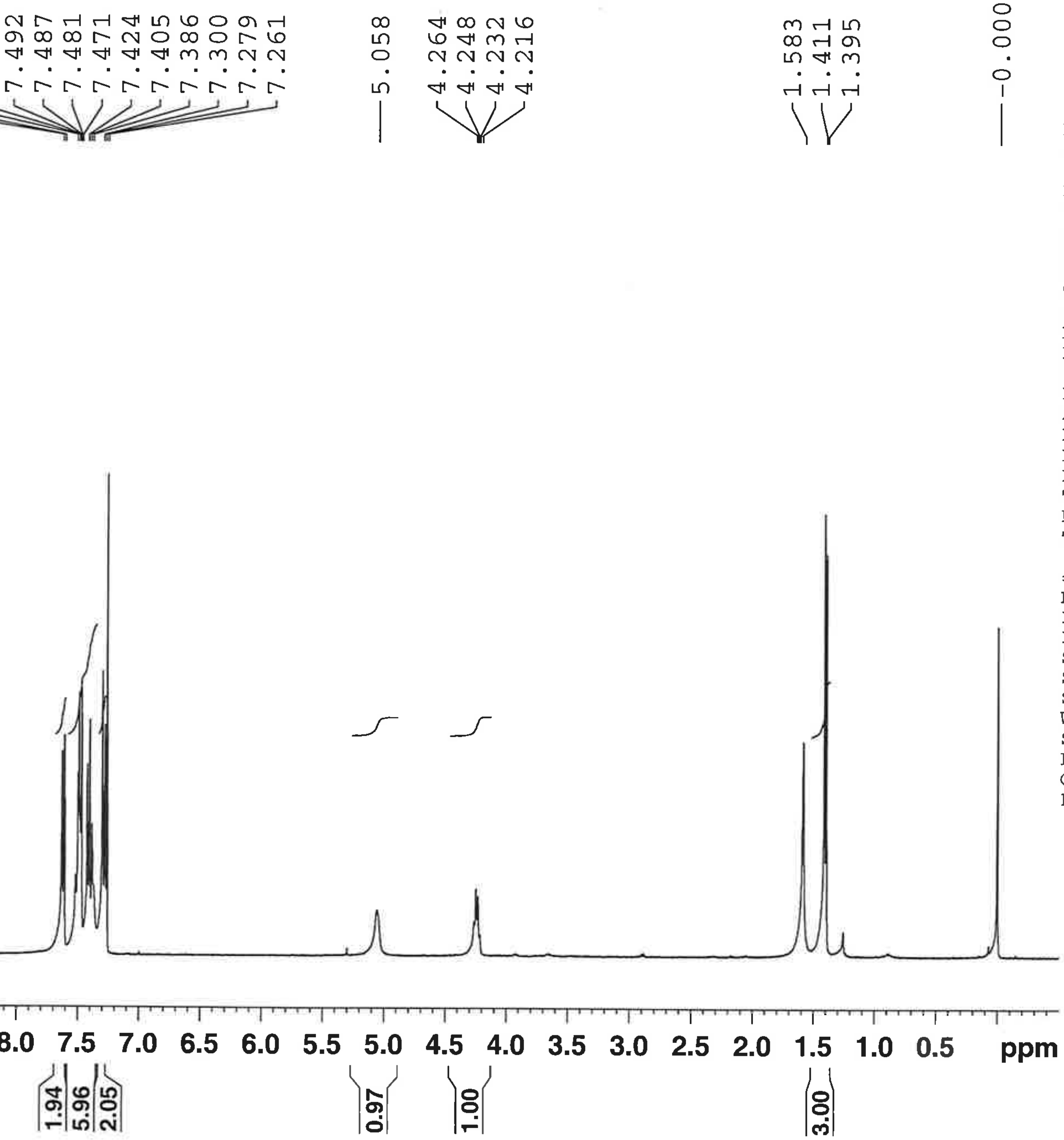

NAME	Aug16-2021-sys	
EXPNO	30	
PROCNO	1	
Date_	20210816	
Time	16.55	
INSTRUM	spect	
PROBHD	5 mm PABBO BB-	
PULPROG	zg30	
TD	65536	
SOLVENT	CDCl3	
NS	16	
DS	2	
SWH	8278.146	Hz
FIDRES	0.126314	Hz
AQ	3.9584243	sec
RG	362	
DW	60.400	usec
DE	6.50	usec
TE	296.3	K
D1	1.00000000	sec
TD0	1	
======== CHANNEL f1 ========		
NUC1	1H	
P1	11.60	usec
PL1	3.00	dB
SFO1	400.1324710	MHz
SI	32768	
SF	400.1300086	MHz
WDW	EM	
SSB	0	
LB	0.30	Hz
GB	0	
PC	1.00	

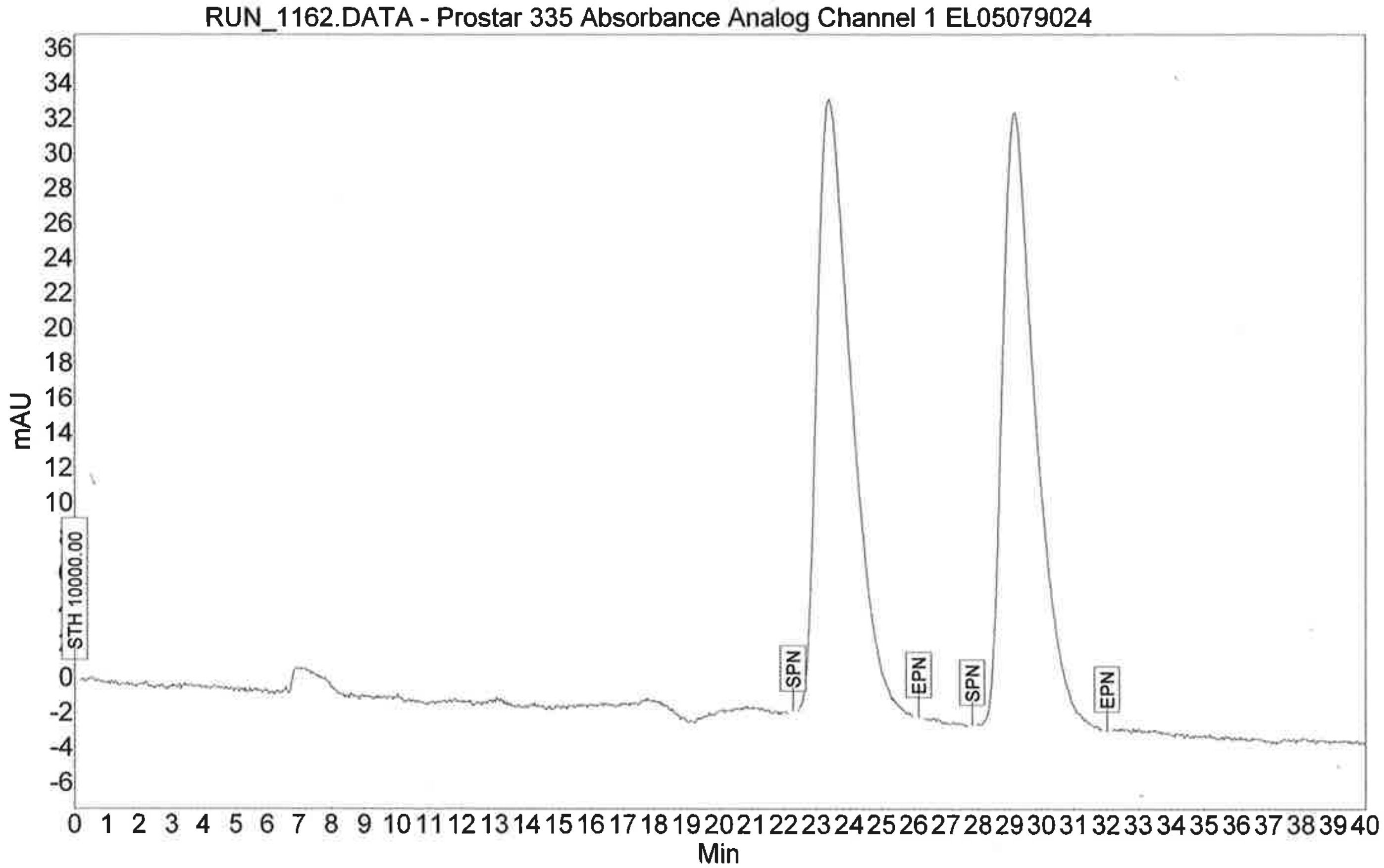

Index	Name	Time [Min]	Quantity [% Area]	Height [mAU]	Area [mAU.Min]	Area % [%]
1	UNKNOWN	23.39	49.80	35.1	42.9	49.801
2	UNKNOWN	29.15	50.20	35.1	43.2	50.199
Total			100.00	70.2	86.1	100.000

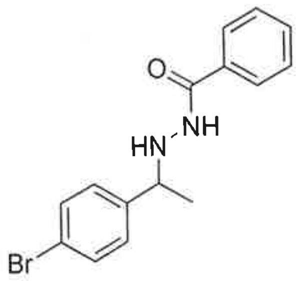

(±)

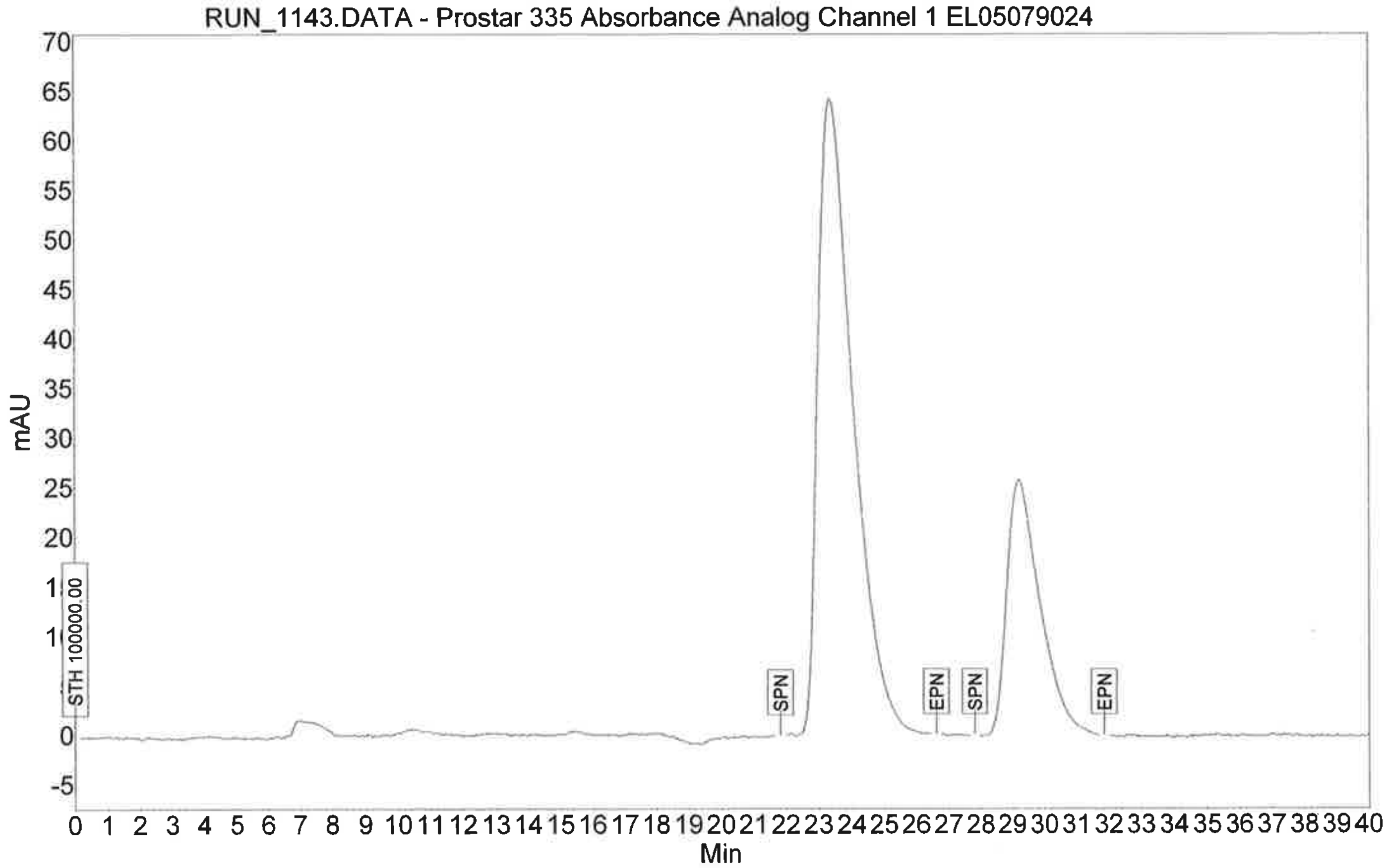

Index	Name	Time [Min]	Quantity [% Area]	Height [mAU]	Area [mAU.Min]	Area % [%]
1	UNKNOWN	23.36	71.91	64.2	79.6	71.906
2	UNKNOWN	29.21	28.09	25.8	31.1	28.094
Total			100.00	90.0	110.7	100.000

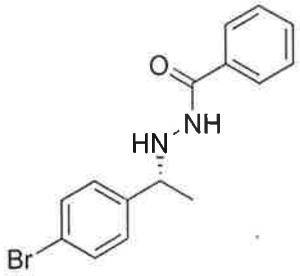

3k

CF3

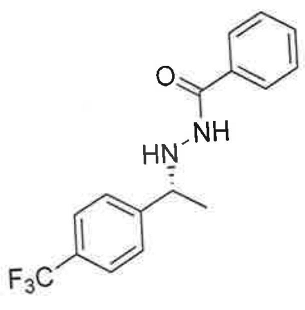

3l

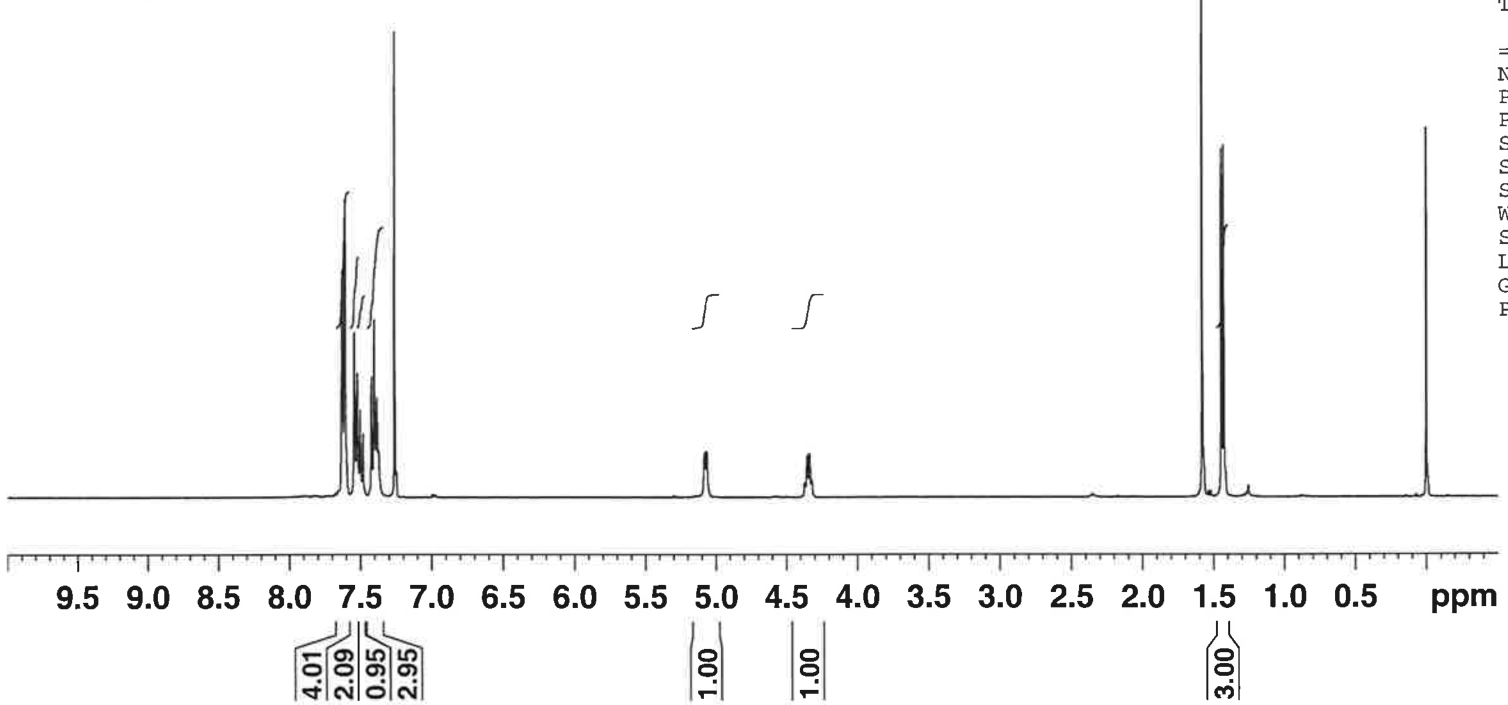

NAME	Aug16-2021-cxu	
EXPNO	30	
PROCNO	1	
Date_	20210816	
Time	17.18	
INSTRUM	spect	
PROBHD	5 mm PABBO BB-	
PULPROG	zg30	
TD	65536	
SOLVENT	CDCl3	
NS	16	
DS	2	
SWH	8278.146	Hz
FIDRES	0.126314	Hz
AQ	3.9584243	sec
RG	362	
DW	60.400	usec
DE	6.50	usec
TE	296.2	K
D1	1.00000000	sec
TD0	1	
========	CHANNEL f1	========
NUC1	1H	
P1	11.60	usec
PL1	3.00	dB
SFO1	400.1324710	MHz
SI	32768	
SF	400.1300089	MHz
WDW	EM	
SSB	0	
LB	0.30	Hz
GB	0	
PC	1.00	

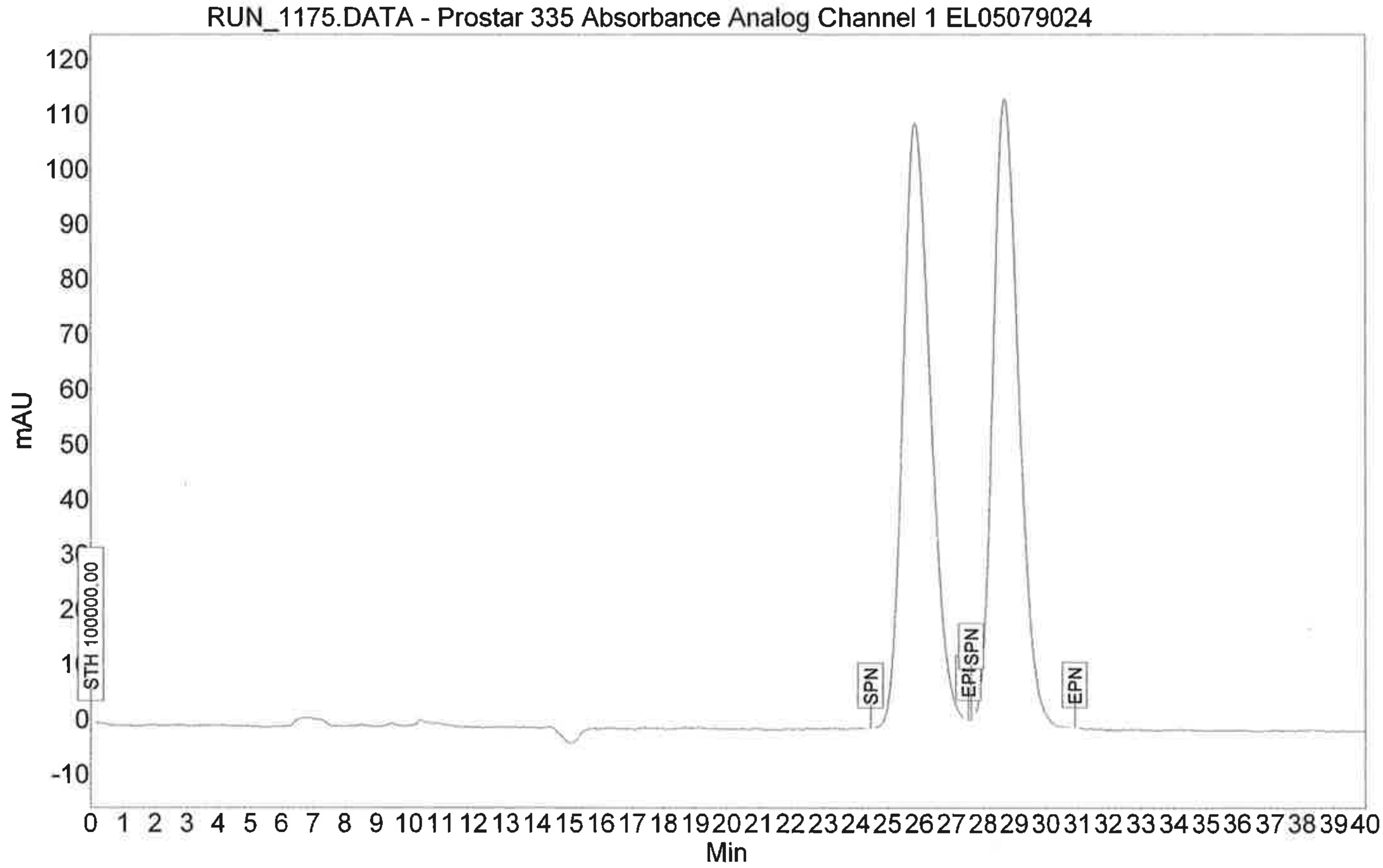

Index	Name	Time [Min]	Quantity [% Area]	Height [mAU]	Area [mAU.Min]	Area % [%]
1	UNKNOWN	25.87	50.00	109.4	109.0	49.996
2	UNKNOWN	28.68	50.00	113.5	109.0	50.004
Total			100.00	222.9	218.0	100.000

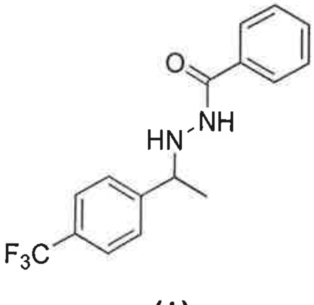

(±)

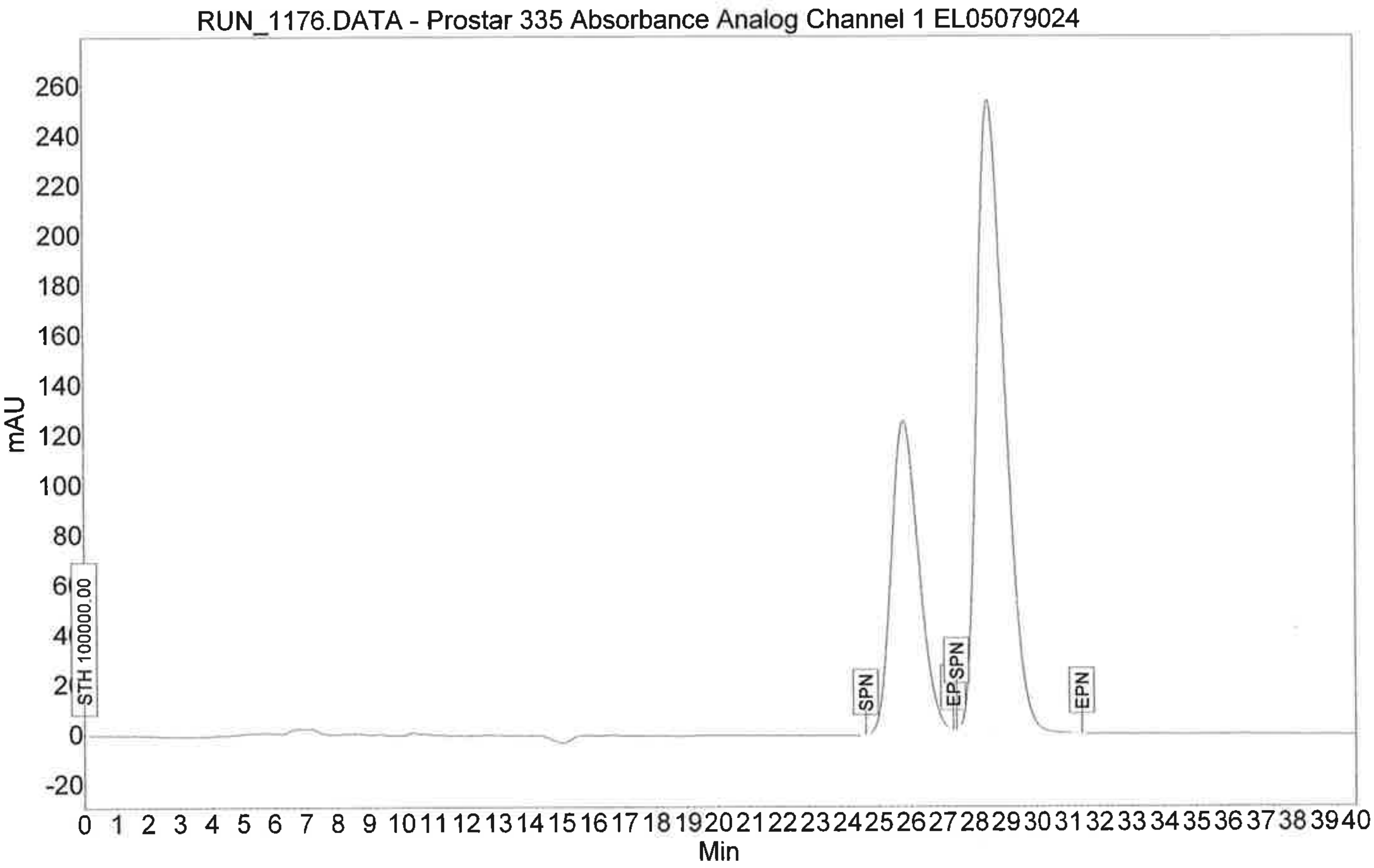

Index	Name	Time [Min]	Quantity [% Area]	Height [mAU]	Area [mAU.Min]	Area % [%]
1	UNKNOWN	25.77	32.27	125.3	124.2	32.271
2	UNKNOWN	28.48	67.73	253.1	260.6	67.729
Total			100.00	378.4	384.8	100.000

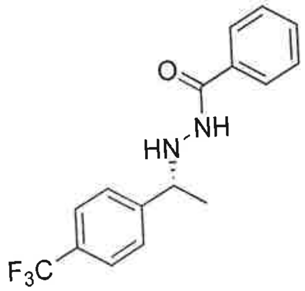

3l

nap

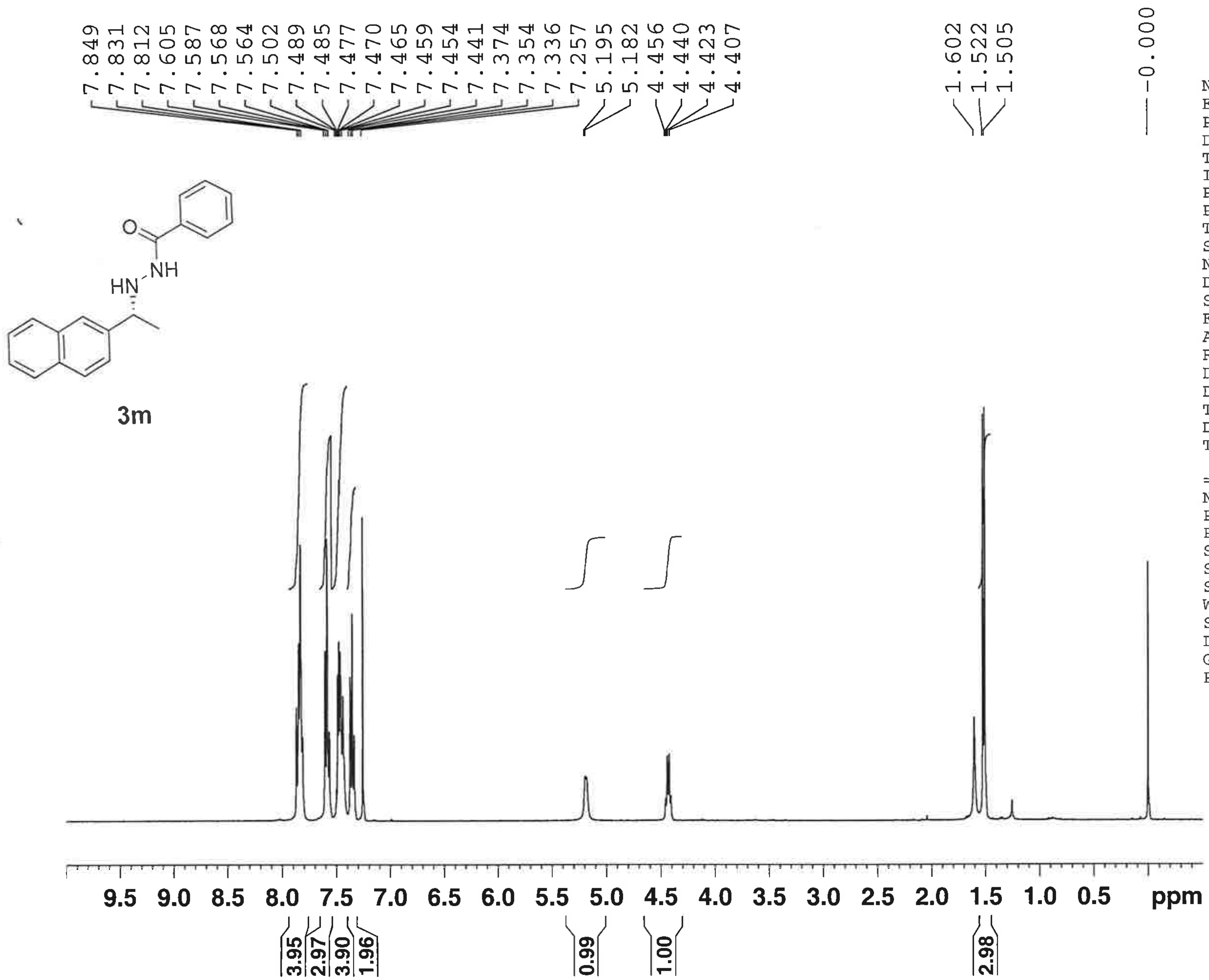

NAME	Aug15-2021-sys	
EXPNO	40	
PROCNO	1	
Date_	20210815	
Time	16.38	
INSTRUM	spect	
PROBHD	5 mm PABBO BB-	
PULPROG	zg30	
TD	65536	
SOLVENT	CDCl3	
NS	16	
DS	2	
SWH	8278.146	Hz
FIDRES	0.126314	Hz
AQ	3.9584243	sec
RG	256	
DW	60.400	usec
DE	6.50	usec
TE	296.3	K
D1	1.00000000	sec
TD0	1	

======== CHANNEL f1 ========		
NUC1	1H	
P1	11.60	usec
PL1	3.00	dB
SFO1	400.1324710	MHz
SI	32768	
SF	400.1300108	MHz
WDW	EM	
SSB	0	
LB	0.30	Hz
GB	0	
PC	1.00	

RUN_1171.DATA - Prostar 335 Absorbance Analog Channel 1 EL05079024

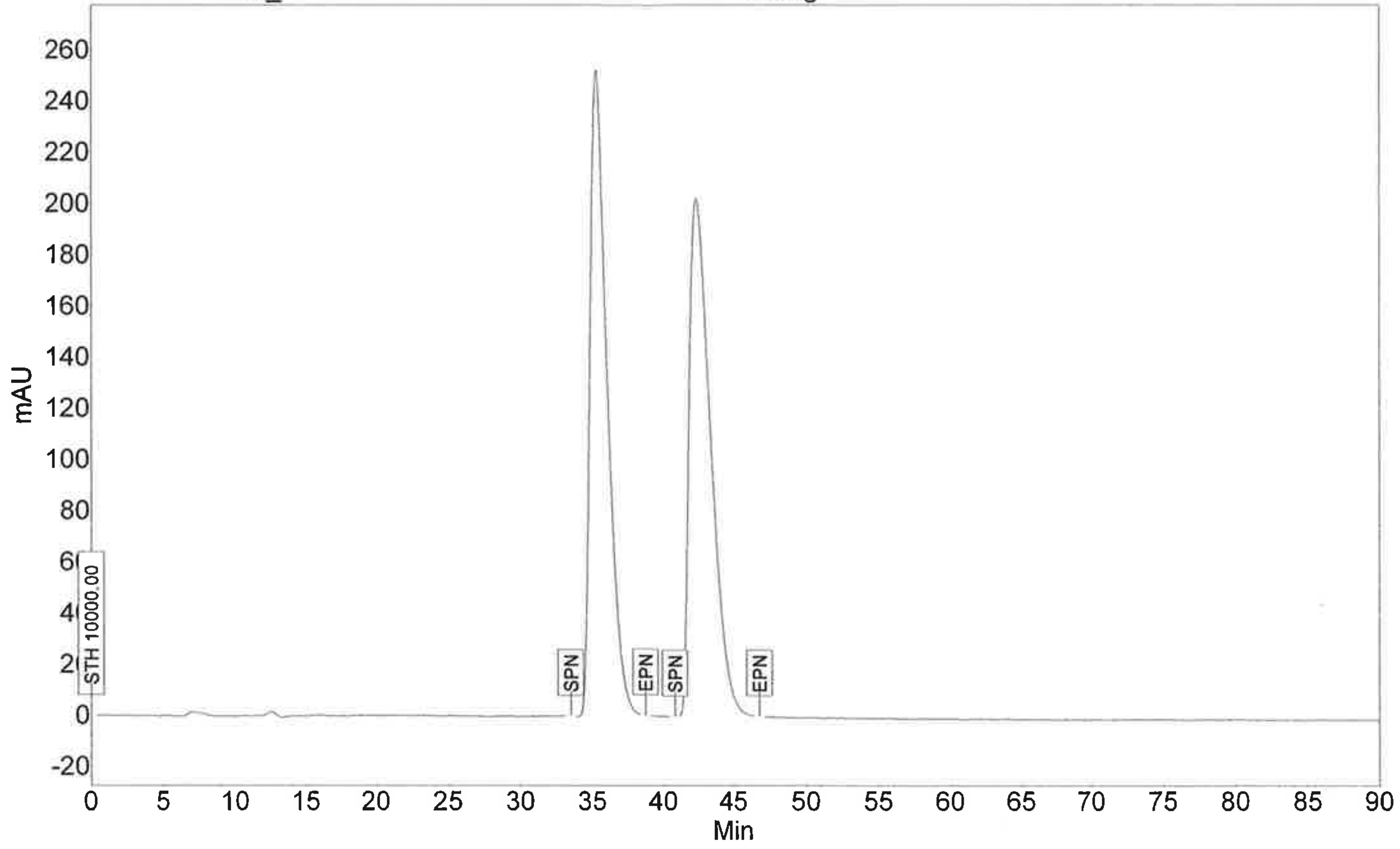

Index	Name	Time [Min]	Quantity [% Area]	Height [mAU]	Area [mAU.Min]	Area % [%]
1	UNKNOWN	35.23	49.53	252.4	352.2	49.530
2	UNKNOWN	42.29	50.47	202.4	358.9	50.470
Total			100.00	454.8	711.1	100.000

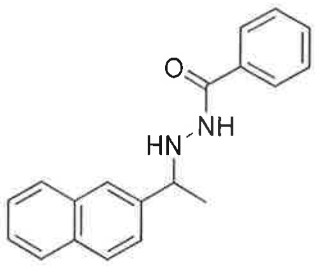

(±)

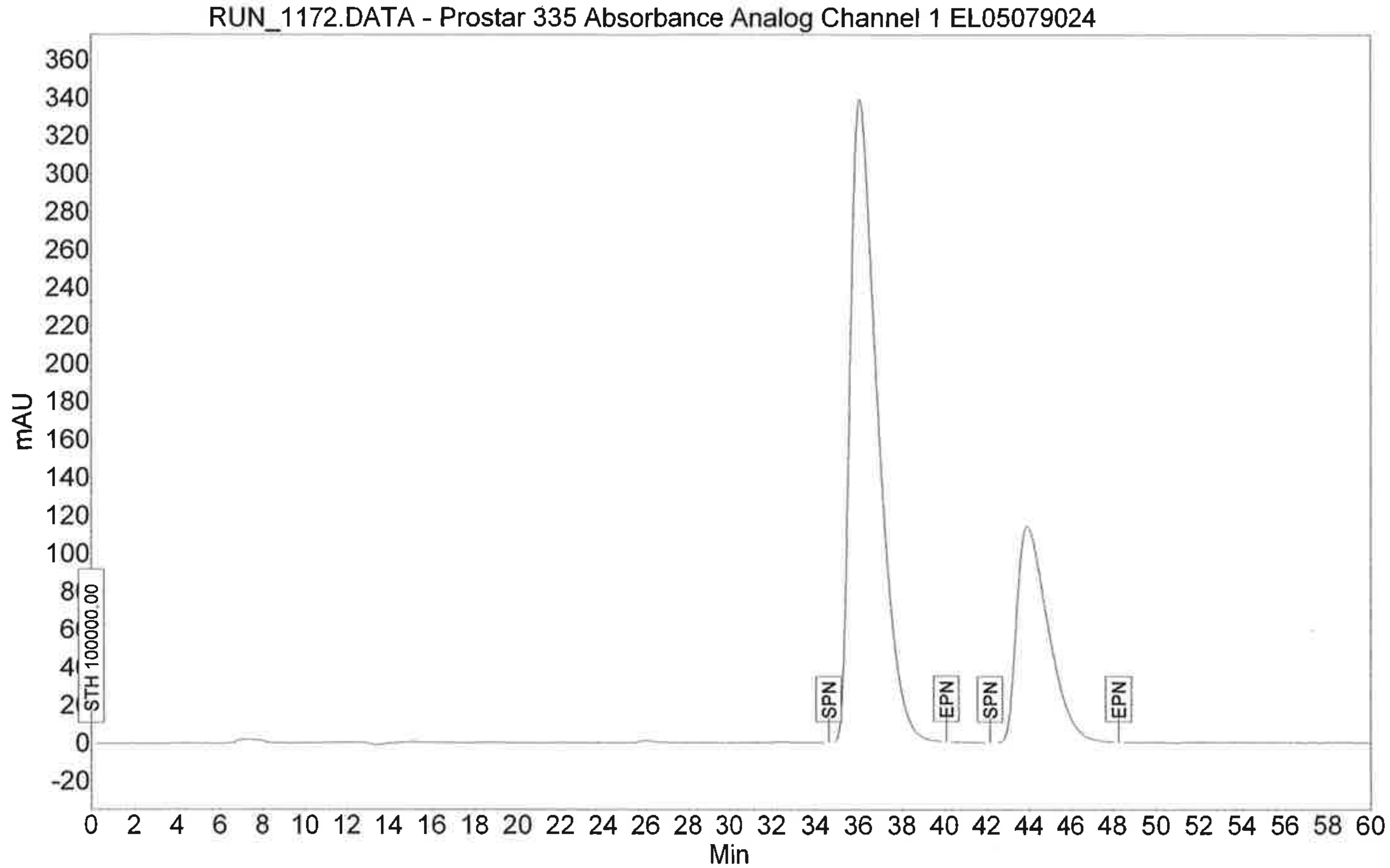

Index	Name	Time [Min]	Quantity [% Area]	Height [mAU]	Area [mAU.Min]	Area % [%]
1	UNKNOWN	36.05	71.55	338.6	498.8	71.547
2	UNKNOWN	43.91	28.45	114.0	198.4	28.453
Total			100.00	452.5	697.2	100.000

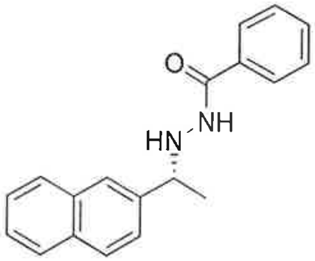

3m

Thio

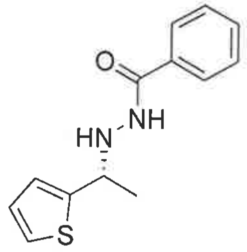

3n

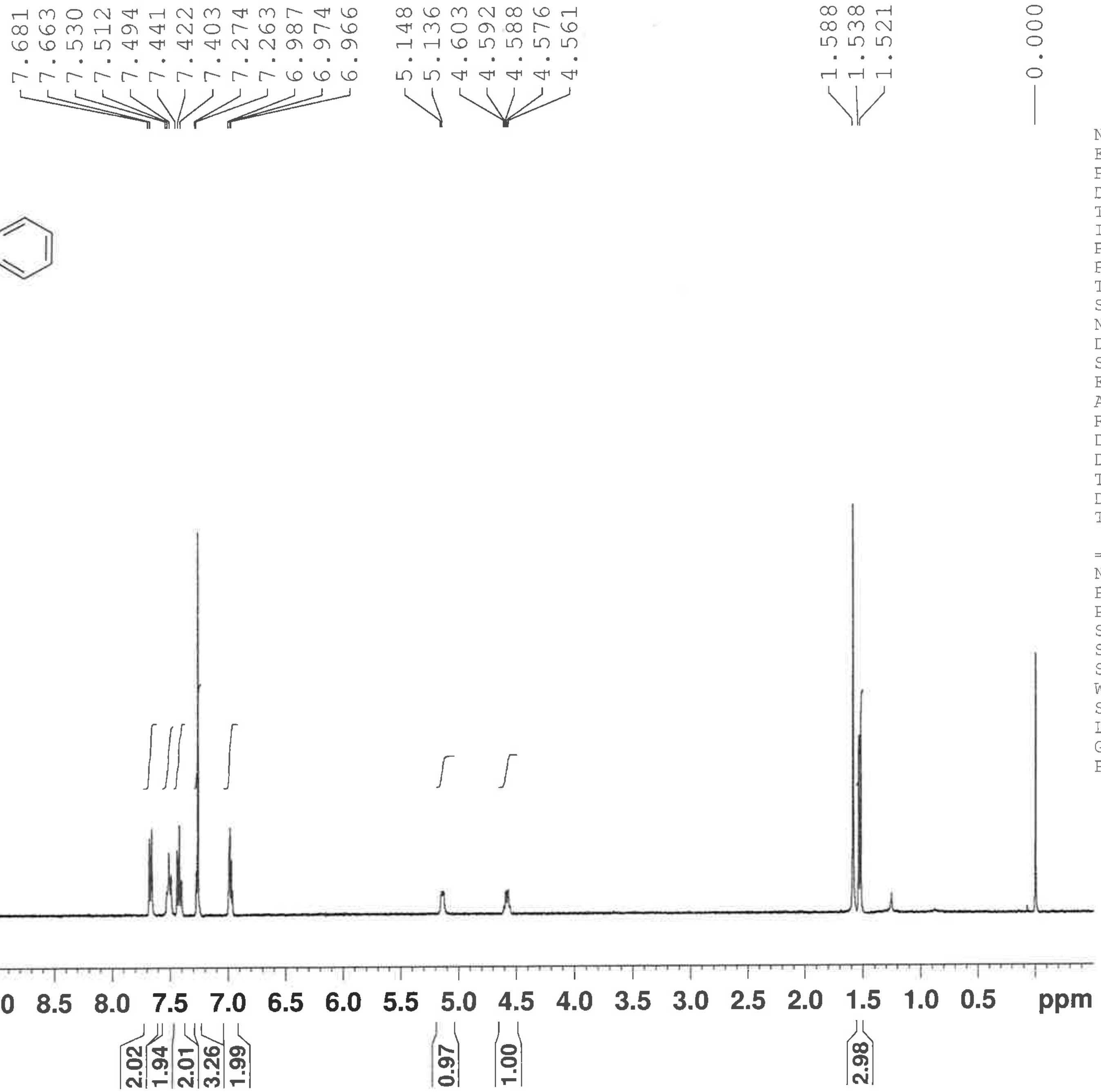

NAME	Aug14-2021-cxu
EXPNO	6
PROCNO	1
Date_	20210814
Time	16.13
INSTRUM	spect
PROBHD	5 mm TBI 1H/31
PULPROG	zg30
TD	65536
SOLVENT	CDCl3
NS	16
DS	2
SWH	8278.146 Hz
FIDRES	0.126314 Hz
AQ	3.9584243 sec
RG	29193
DW	60.400 usec
DE	6.50 usec
TE	300.0 K
D1	1.00000000 sec
TD0	1

======== CHANNEL f1 ========

NUC1	1H
P1	18.75 usec
PL1	0.50 dB
SFO1	400.1324710 MHz
SI	32768
SF	400.1300085 MHz
WDW	EM
SSB	0
LB	0.30 Hz
GB	0
PC	1.00

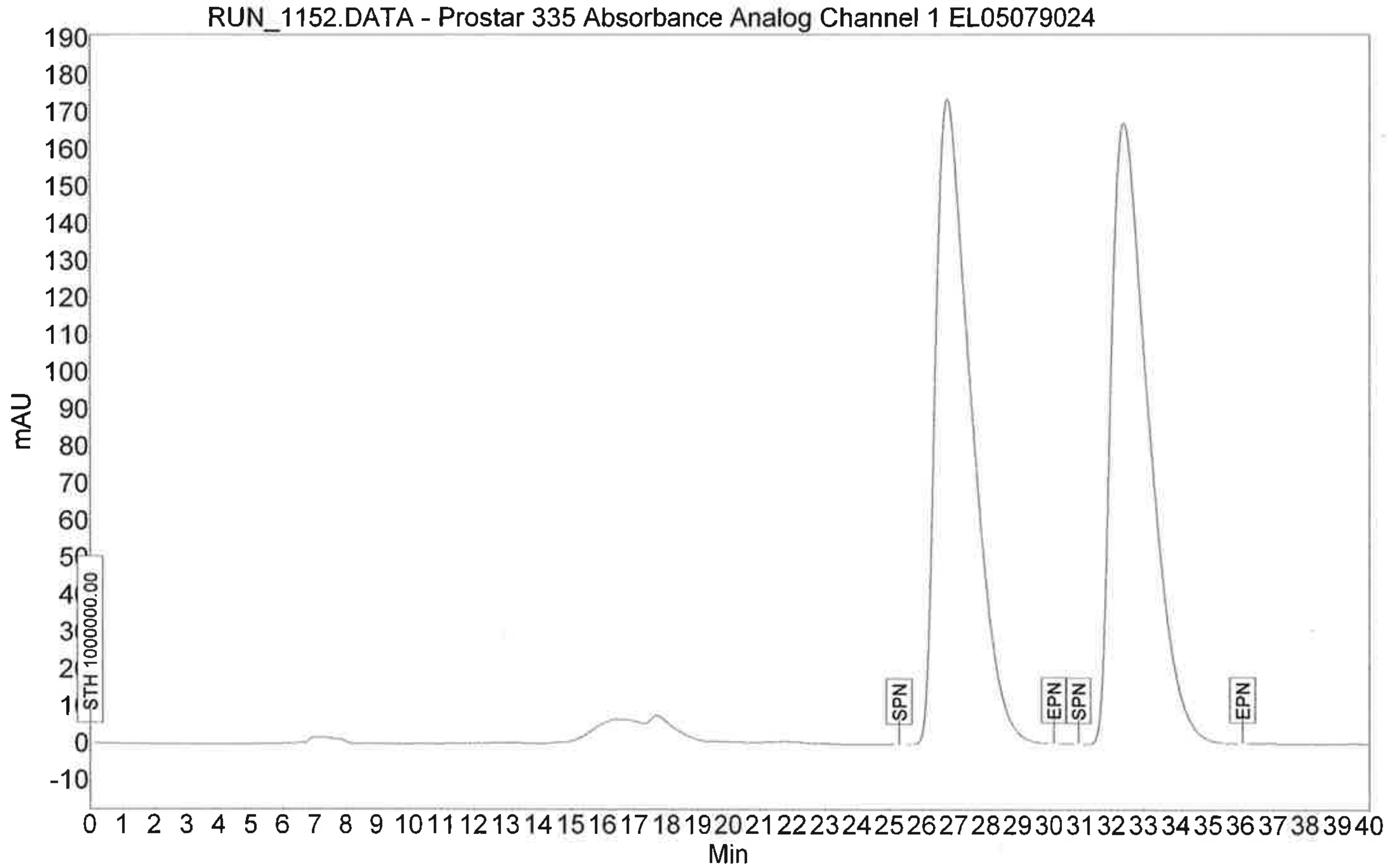

Index	Name	Time [Min]	Quantity [% Area]	Height [mAU]	Area [mAU.Min]	Area % [%]
1	UNKNOWN	26.77	49.94	173.7	224.1	49.941
2	UNKNOWN	32.36	50.06	167.0	224.6	50.059
Total			100.00	340.7	448.6	100.000

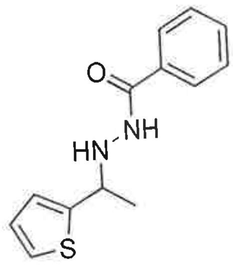

(±)

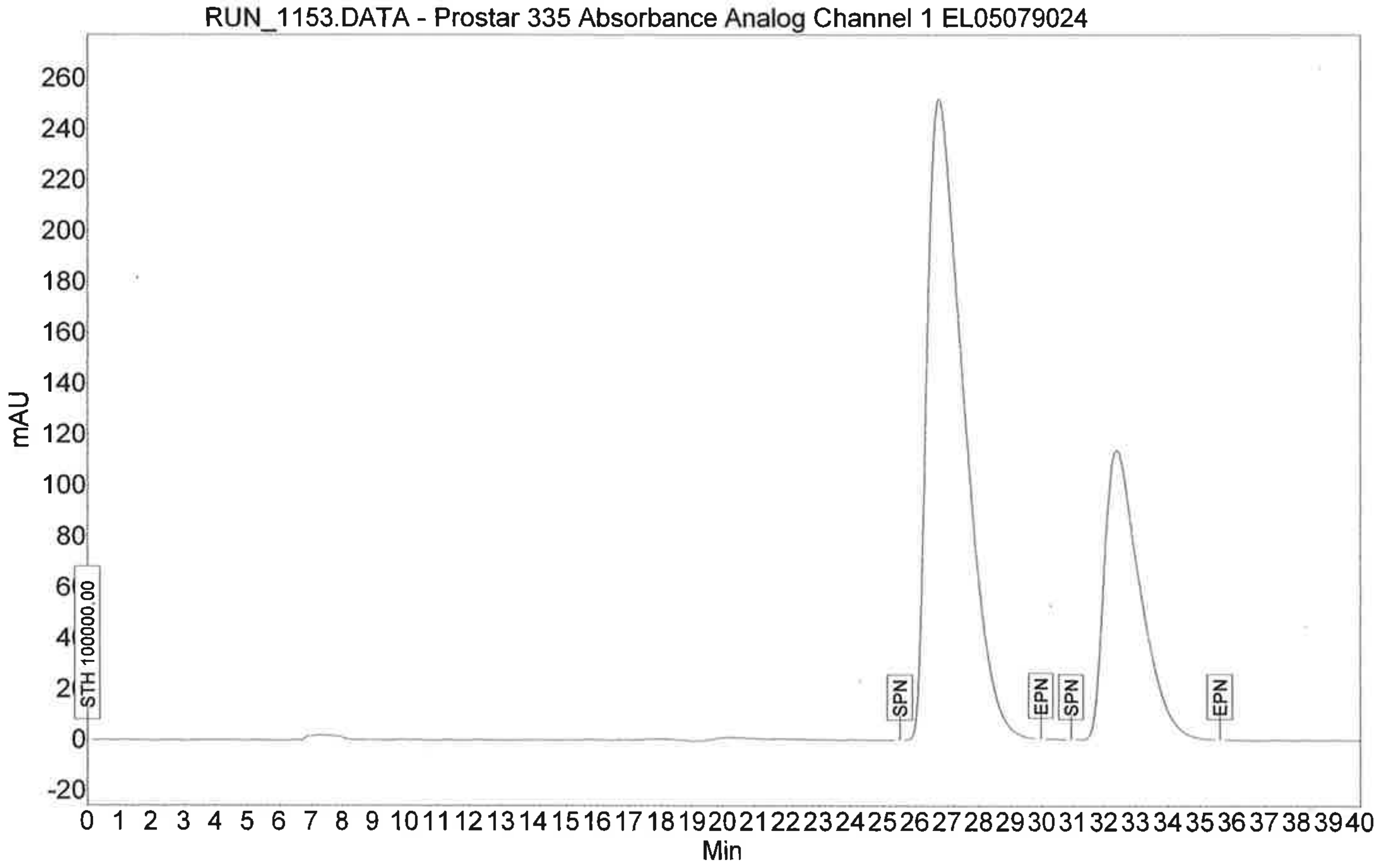

Index	Name	Time [Min]	Quantity [% Area]	Height [mAU]	Area [mAU.Min]	Area % [%]
1	UNKNOWN	26.72	69.16	251.6	333.2	69.159
2	UNKNOWN	32.39	30.84	113.8	148.6	30.841
Total			100.00	365.4	481.7	100.000

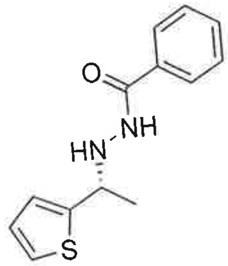

3n

Et

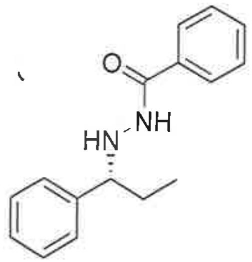

3o

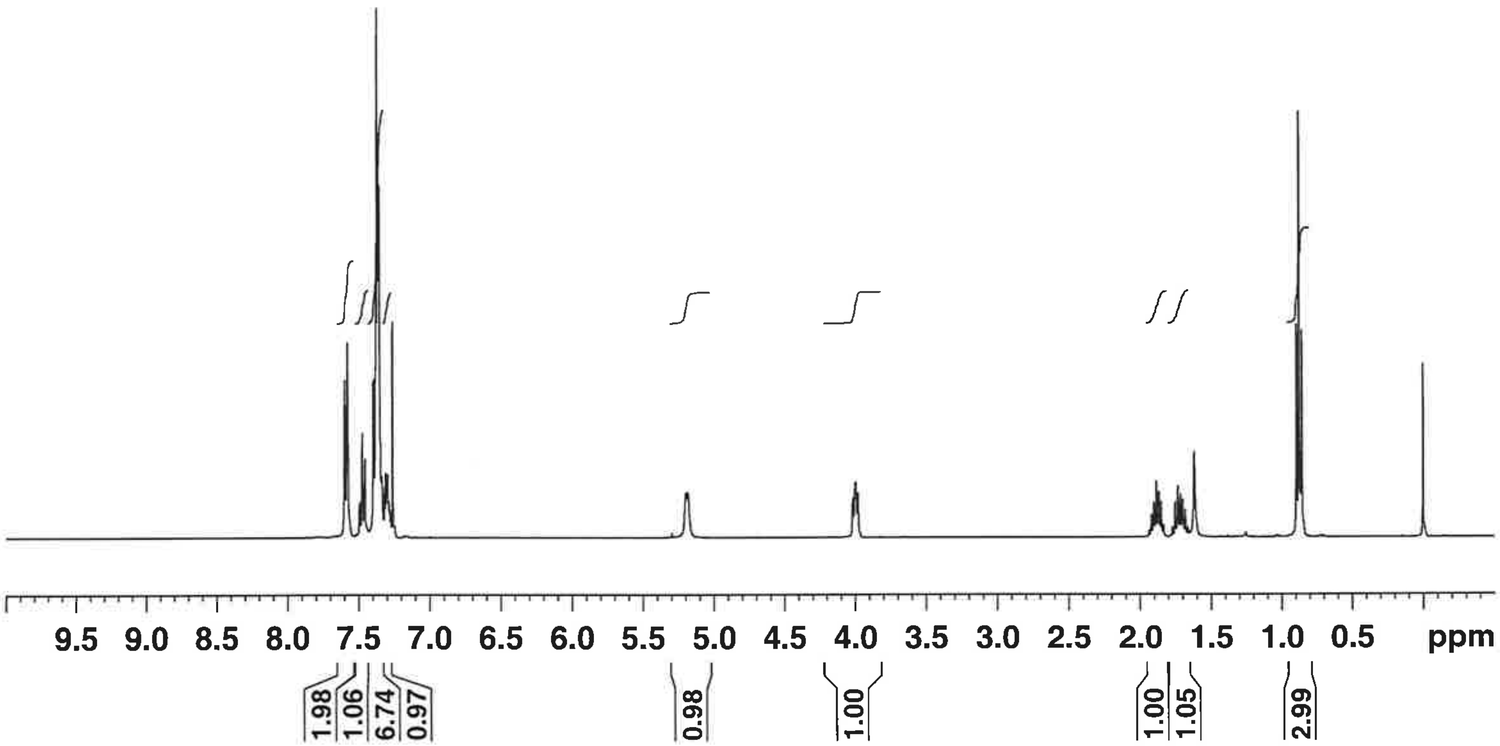

NAME	Aug19-2021-sys
EXPNO	20
PROCNO	1
Date_	20210819
Time	15.56
INSTRUM	spect
PROBHD	5 mm PABBO BB-
PULPROG	zg30
TD	65536
SOLVENT	CDCl3
NS	16
DS	2
SWH	8278.146 Hz
FIDRES	0.126314 Hz
AQ	3.9584243 sec
RG	256
DW	60.400 usec
DE	6.50 usec
TE	296.6 K
D1	1.00000000 sec
TD0	1
======== CHANNEL f1 ========	
NUC1	1H
P1	11.60 usec
PL1	3.00 dB
SFO1	400.1324710 MHz
SI	32768
SF	400.1300097 MHz
WDW	EM
SSB	0
LB	0.30 Hz
GB	0
PC	1.00

RUN_1158.DATA - Prostar 335 Absorbance Analog Channel 1 EL05079024

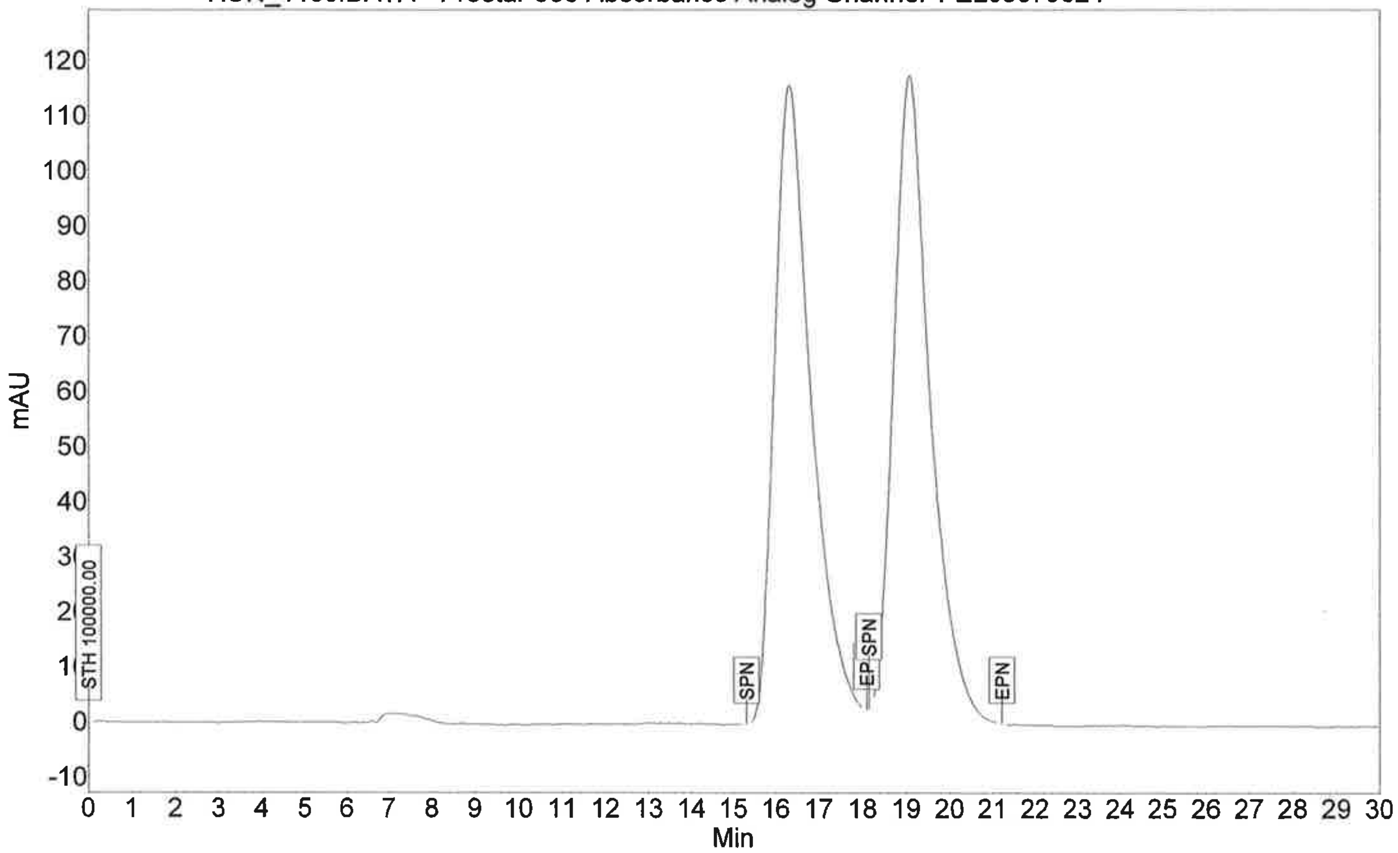

Index	Name	Time [Min]	Quantity [% Area]	Height [mAU]	Area [mAU.Min]	Area % [%]
1	UNKNOWN	16.31	50.19	115.0	112.6	50.194
2	UNKNOWN	19.08	49.81	115.9	111.8	49.806
Total			100.00	230.9	224.4	100.000

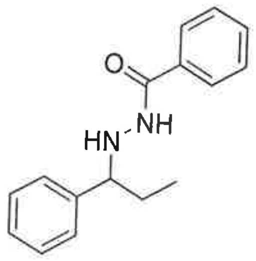

(±)

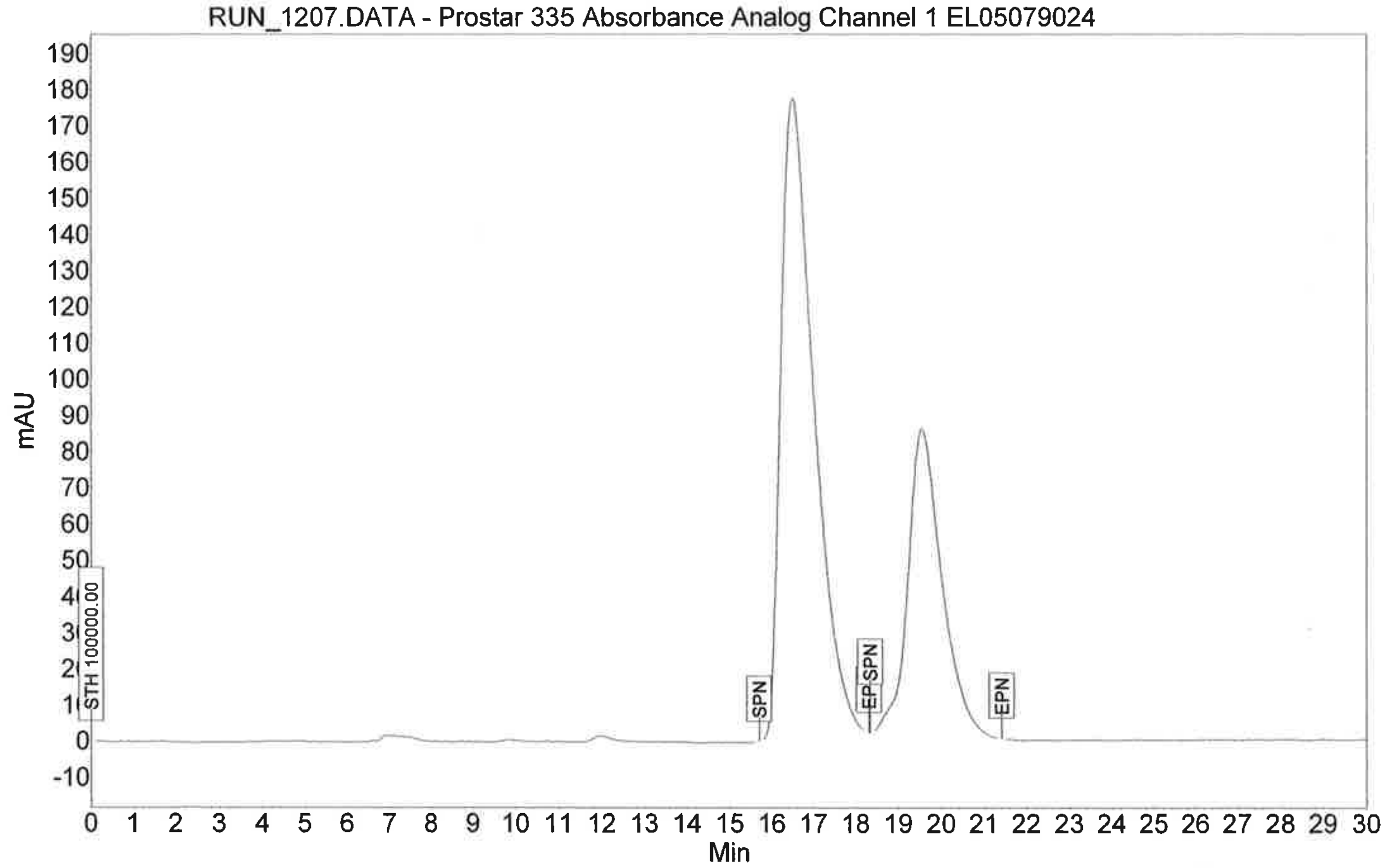

Index	Name	Time [Min]	Quantity [% Area]	Height [mAU]	Area [mAU.Min]	Area % [%]
1	UNKNOWN	16.49	67.80	176.9	163.0	67.802
2	UNKNOWN	19.55	32.20	84.6	77.4	32.198
Total			100.00	261.5	240.4	100.000

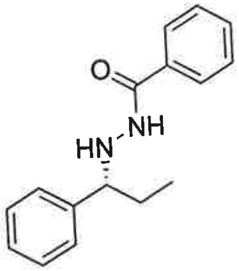

3o

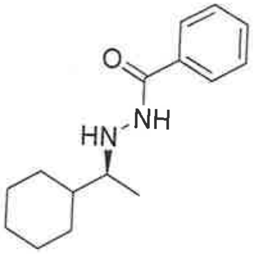

3p

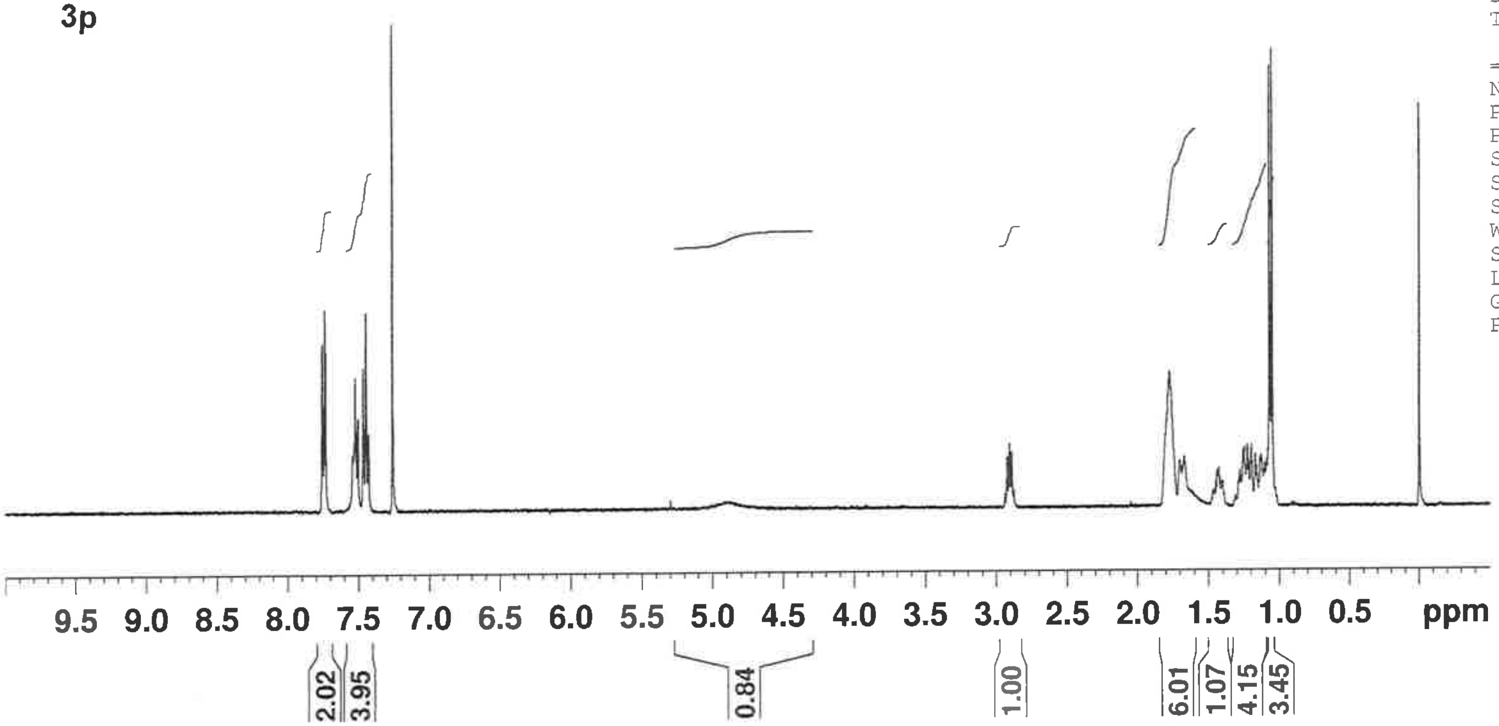

NAME	Aug05-2021-cxu
EXPNO	2
PROCNO	1
Date_	20210805
Time	15.46
INSTRUM	spect
PROBHD	5 mm TBI 1H/31
PULPROG	zg30
TD	65536
SOLVENT	CDCl3
NS	16
DS	2
SWH	8278.146 Hz
FIDRES	0.126314 Hz
AQ	3.9584243 sec
RG	16384
DW	60.400 usec
DE	6.50 usec
TE	300.0 K
D1	1.00000000 sec
TD0	1
======== CHANNEL f1 ========	
NUC1	1H
P1	18.75 usec
PL1	0.50 dB
SFO1	400.1324710 MHz
SI	32768
SF	400.1300083 MHz
WDW	EM
SSB	0
LB	0.30 Hz
GB	0
PC	1.00

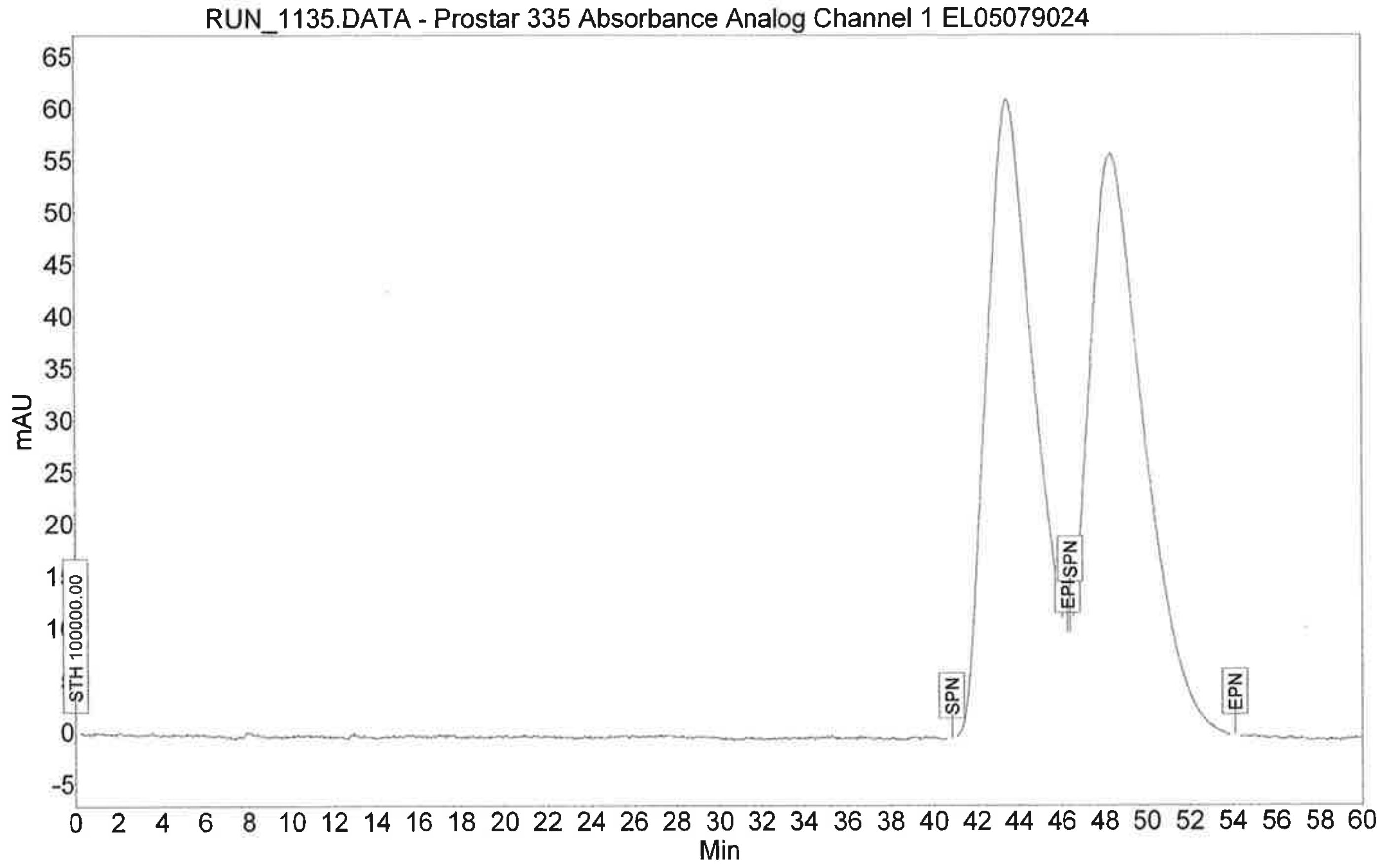

Index	Name	Time [Min]	Quantity [% Area]	Height [mAU]	Area [mAU.Min]	Area % [%]
1	UNKNOWN	43.41	50.49	56.8	135.1	50.486
2	UNKNOWN	48.35	49.51	48.6	132.5	49.514
Total			100.00	105.4	267.7	100.000

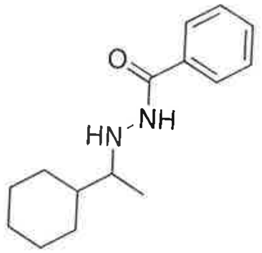

(±)

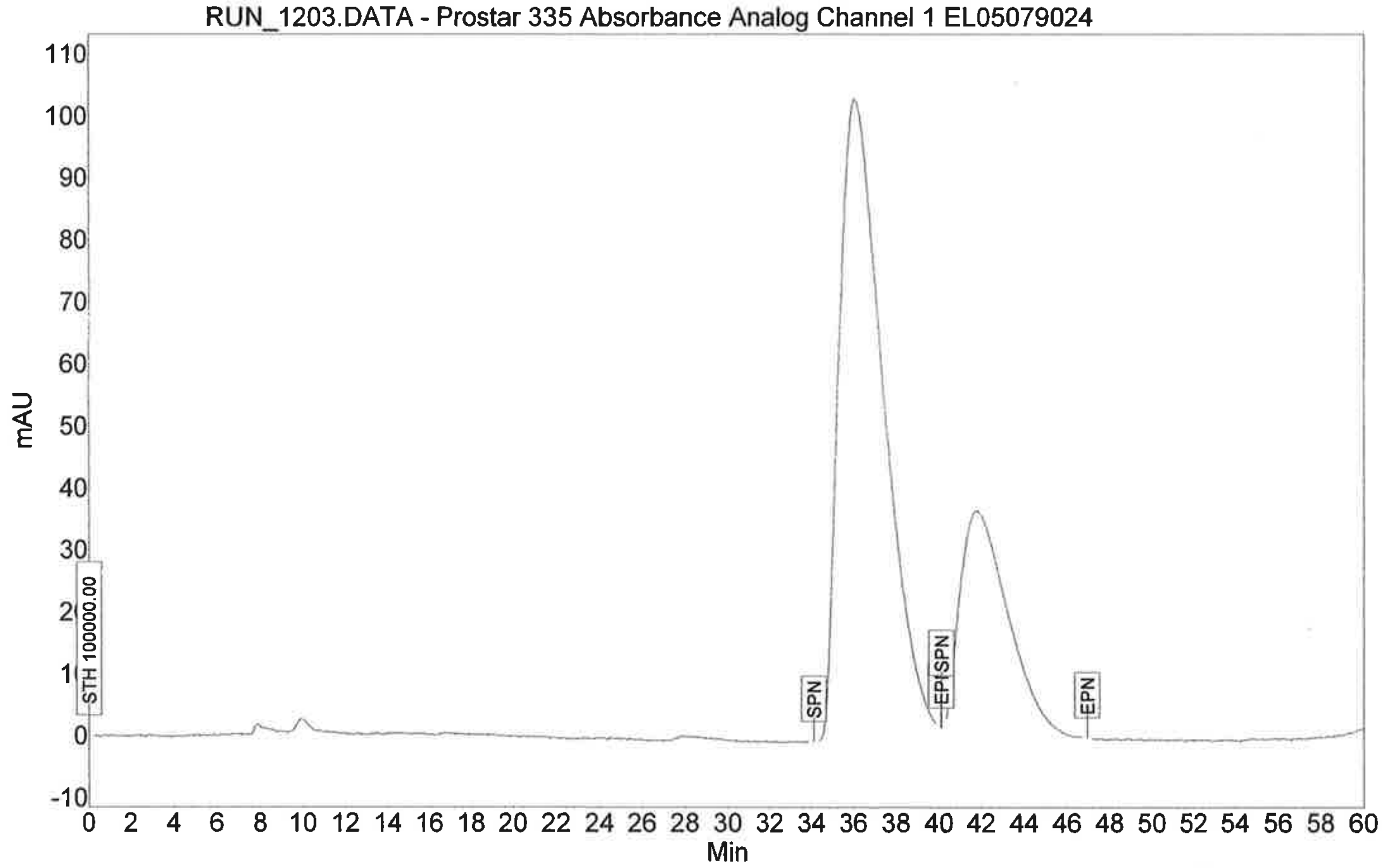

Index	Name	Time [Min]	Quantity [% Area]	Height [mAU]	Area [mAU.Min]	Area % [%]
1	UNKNOWN	36.08	72.91	103.0	248.6	72.907
2	UNKNOWN	41.83	27.09	35.5	92.4	27.093
Total			100.00	138.5	341.0	100.000

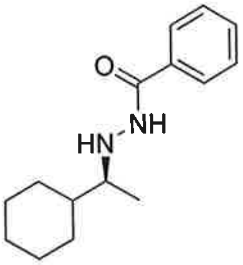

3p

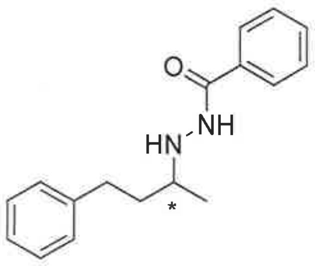

3q

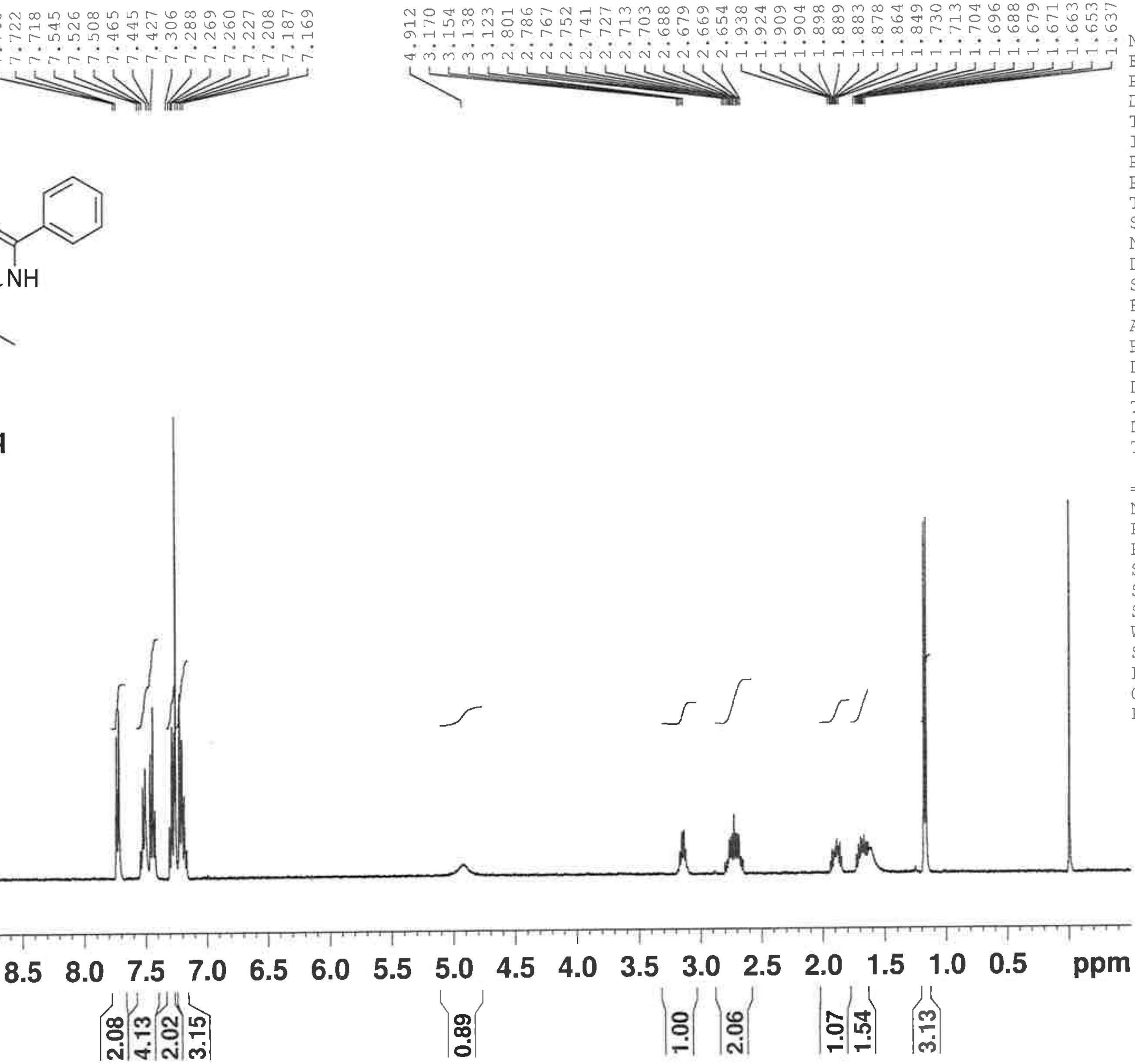

NAME	Aug05-2021-cxu
EXPNO	1
PROCNO	1
Date_	20210805
Time	15.38
INSTRUM	spect
PROBHD	5 mm TBI 1H/31
PULPROG	zg30
TD	65536
SOLVENT	CDCl3
NS	16
DS	2
SWH	8278.146 Hz
FIDRES	0.126314 Hz
AQ	3.9584243 sec
RG	16384
DW	60.400 usec
DE	6.50 usec
TE	300.0 K
D1	1.00000000 sec
TD0	1

======== CHANNEL f1 ========

NUC1	1H
P1	18.75 usec
PL1	0.50 dB
SFO1	400.1324710 MHz
SI	32768
SF	400.1300093 MHz
WDW	EM
SSB	0
LB	0.30 Hz
GB	0
PC	1.00

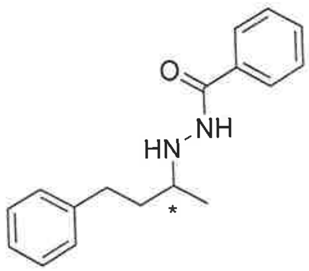

3q

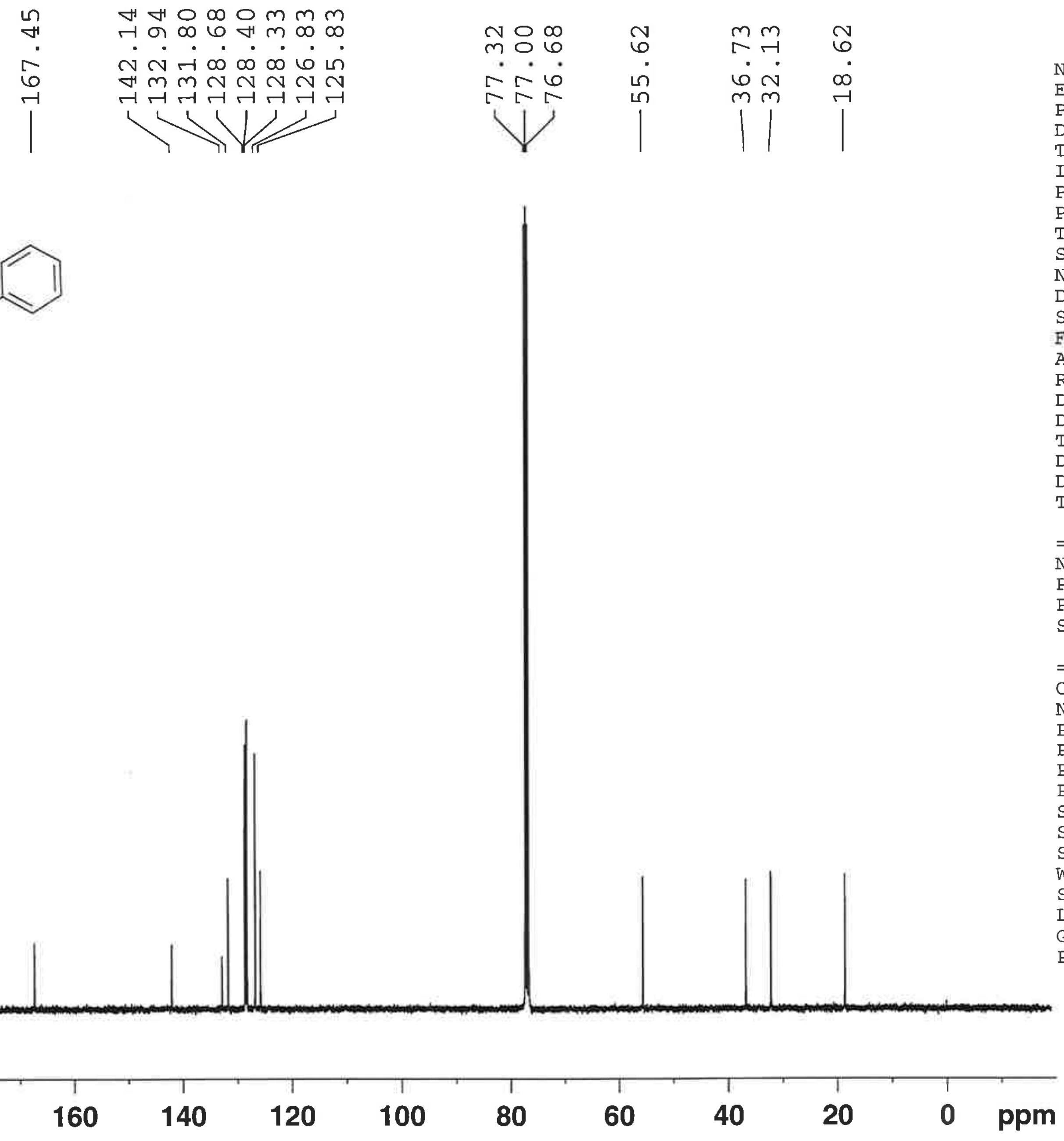

NAME	Aug09-2021-cxu	
EXPNO	10	
PROCNO	1	
Date_	20210810	
Time	6.29	
INSTRUM	spect	
PROBHD	5 mm PABBO BB-	
PULPROG	zgpg30	
TD	65536	
SOLVENT	CDCl3	
NS	10240	
DS	4	
SWH	23980.814	Hz
FIDRES	0.365918	Hz
AQ	1.3664756	sec
RG	32768	
DW	20.850	usec
DE	6.50	usec
TE	301.9	K
D1	2.00000000	sec
D11	0.03000000	sec
TD0	1	
======== CHANNEL f1 ========		
NUC1	13C	
P1	14.75	usec
PL1	0.00	dB
SFO1	100.6228298	MHz
======== CHANNEL f2 ========		
CPDPRG2	waltz16	
NUC2	1H	
PCPD2	80.00	usec
PL2	-2.00	dB
PL12	12.54	dB
PL13	15.00	dB
SFO2	400.1316005	MHz
SI	32768	
SF	100.6127704	MHz
WDW	EM	
SSB	0	
LB	1.00	Hz
GB	0	
PC	1.40	

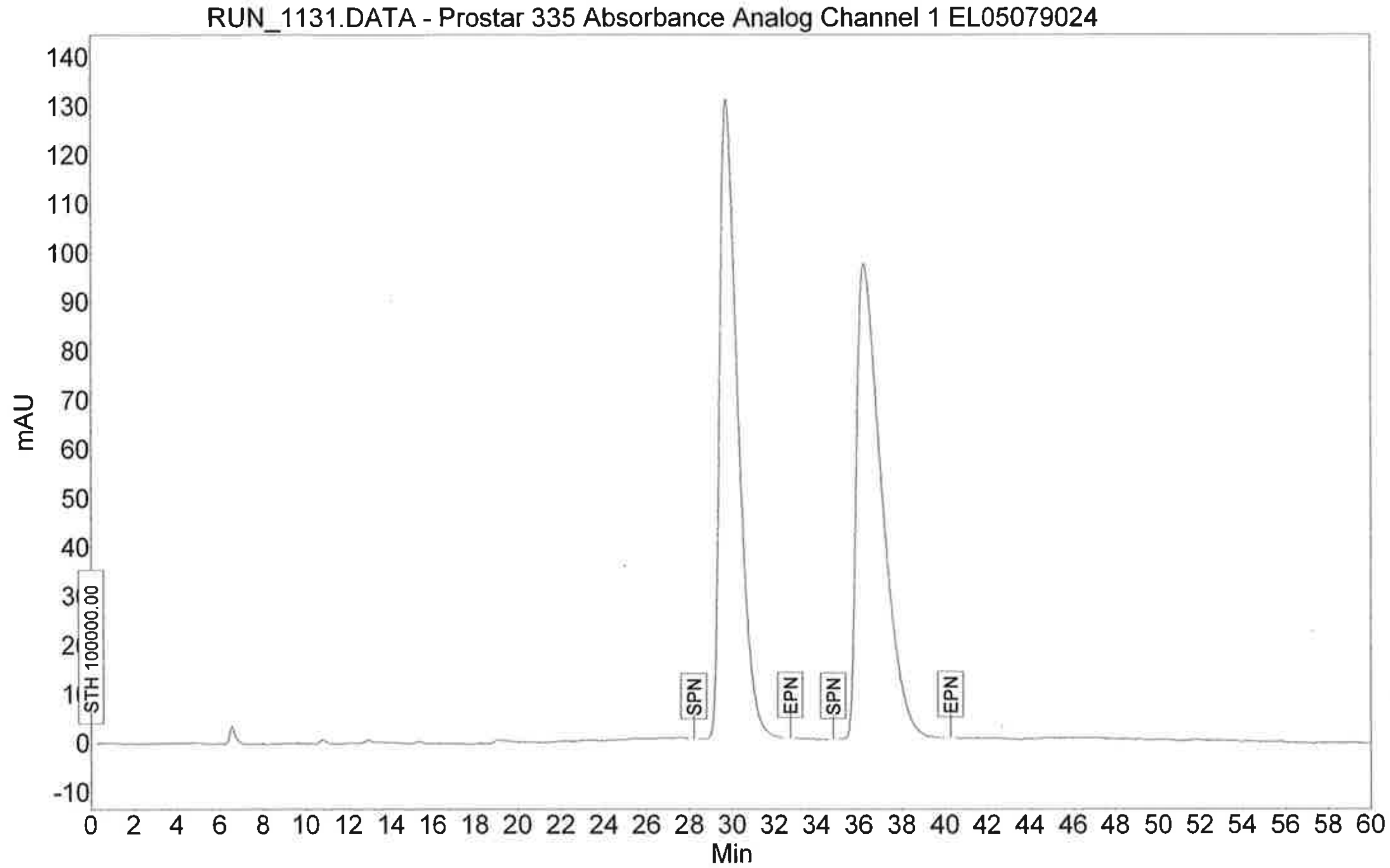

Index	Name	Time [Min]	Quantity [% Area]	Height [mAU]	Area [mAU.Min]	Area % [%]
1	UNKNOWN	29.72	49.68	130.5	128.8	49.681
2	UNKNOWN	36.25	50.32	97.0	130.5	50.319
Total			100.00	227.6	259.3	100.000

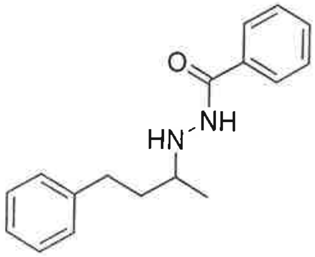

(±)

RUN_1133.DATA - Prostar 335 Absorbance Analog Channel 1 EL05079024

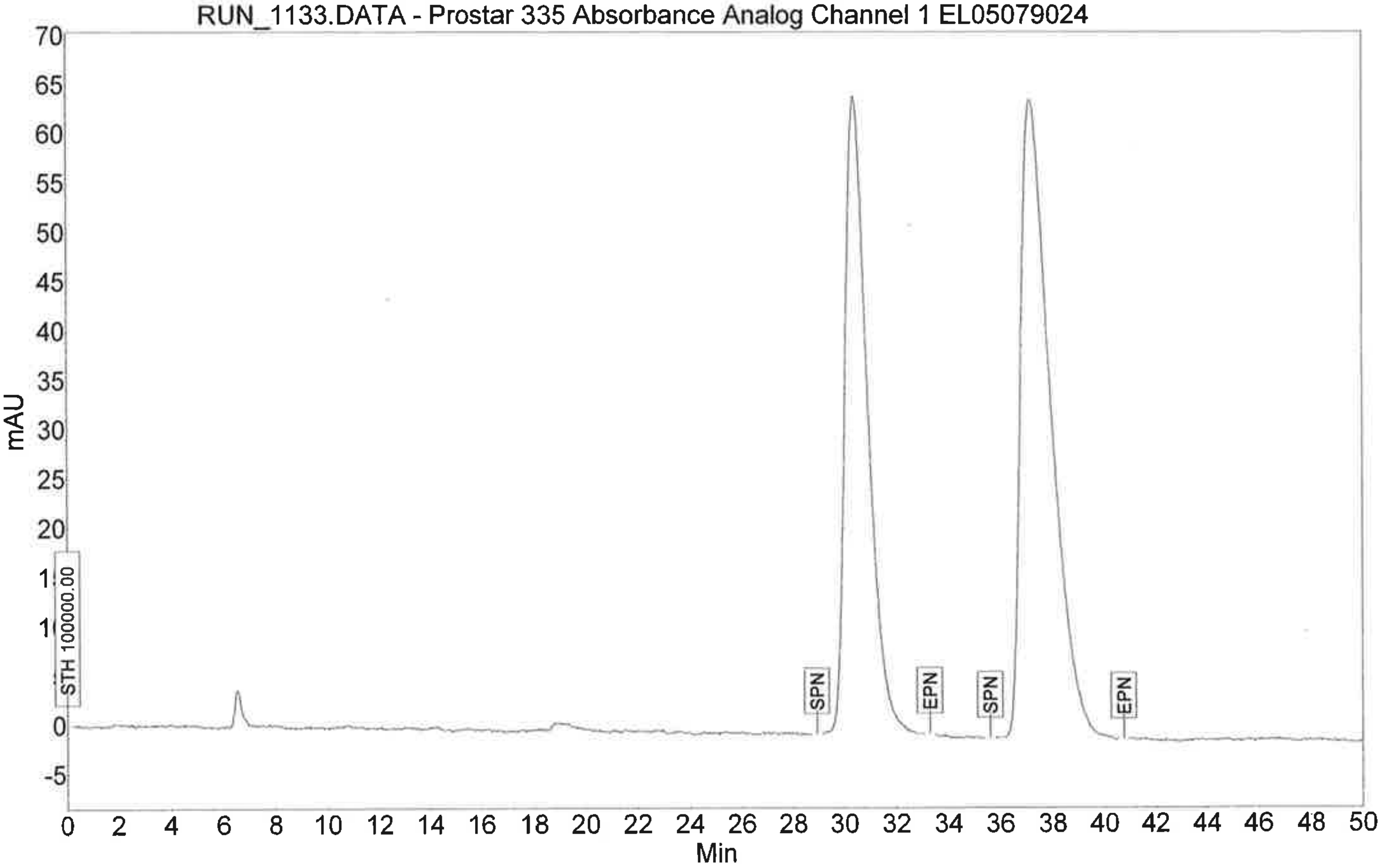

Index	Name	Time [Min]	Quantity [% Area]	Height [mAU]	Area [mAU.Min]	Area % [%]
1	UNKNOWN	30.40	42.98	64.8	64.8	42.983
2	UNKNOWN	37.17	57.02	64.9	85.9	57.017
Total			100.00	129.7	150.7	100.000

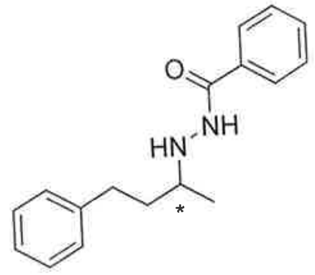

3q

RUN_1188.DATA - Prostar 335 Absorbance Analog Channel 2 EL05079024

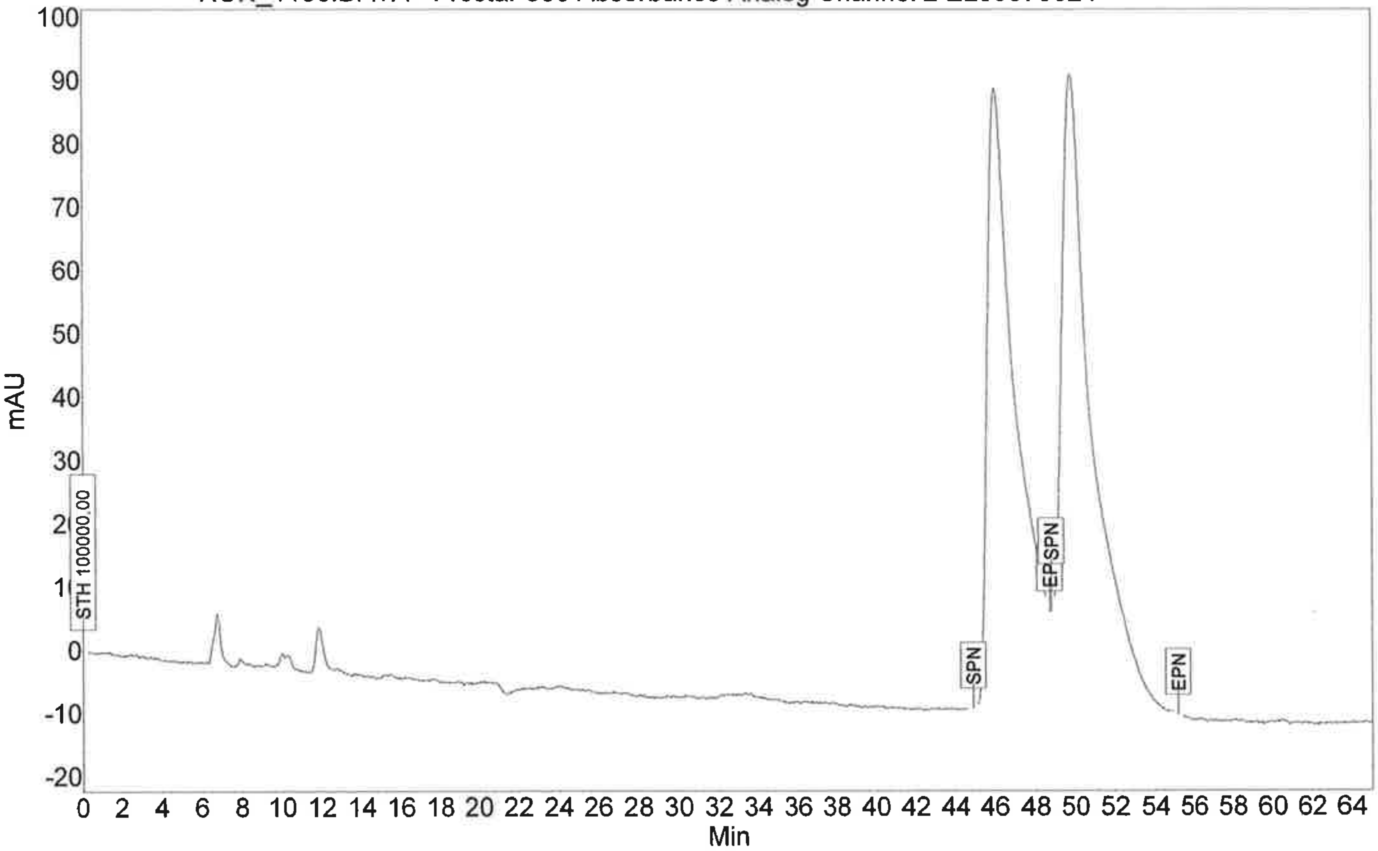

Index	Name	Time [Min]	Quantity [% Area]	Height [mAU]	Area [mAU.Min]	Area % [%]
1	UNKNOWN	45.99	49.70	93.6	135.3	49.697
2	UNKNOWN	49.79	50.30	87.6	136.9	50.303
Total			100.00	181.2	272.2	100.000

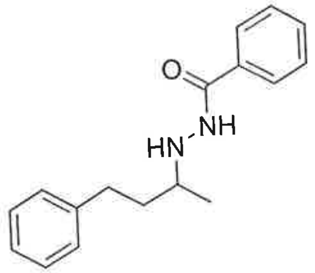

(±)

RUN_1189.DATA - Prostar 335 Absorbance Analog Channel 2 EL05079024

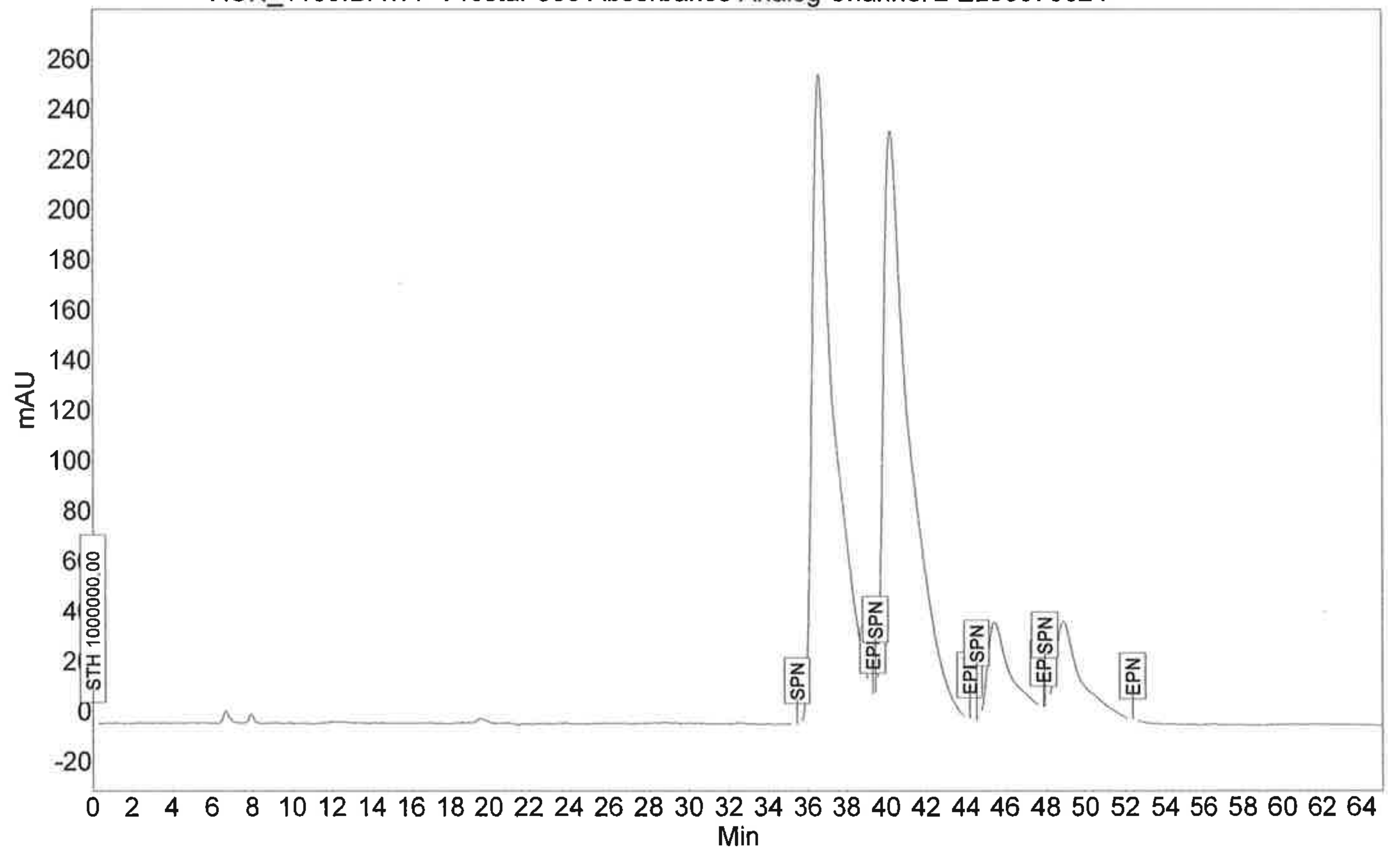

Index	Name	Time [Min]	Quantity [% Area]	Height [mAU]	Area [mAU.Min]	Area % [%]
1	UNKNOWN	36.56	43.45	255.5	332.7	43.446
2	UNKNOWN	40.21	44.01	226.0	337.1	44.014
3	UNKNOWN	45.41	6.12	37.6	46.8	6.115
4	UNKNOWN	48.85	6.43	35.1	49.2	6.426
Total			100.00	554.3	765.8	100.000

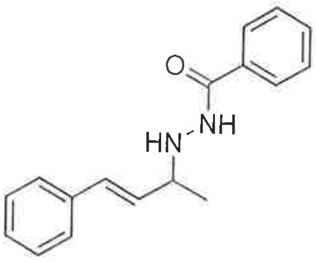

(±)

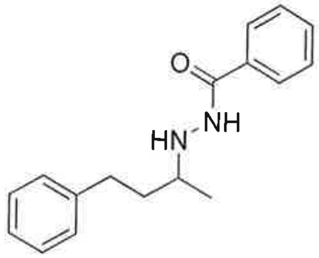

(±)

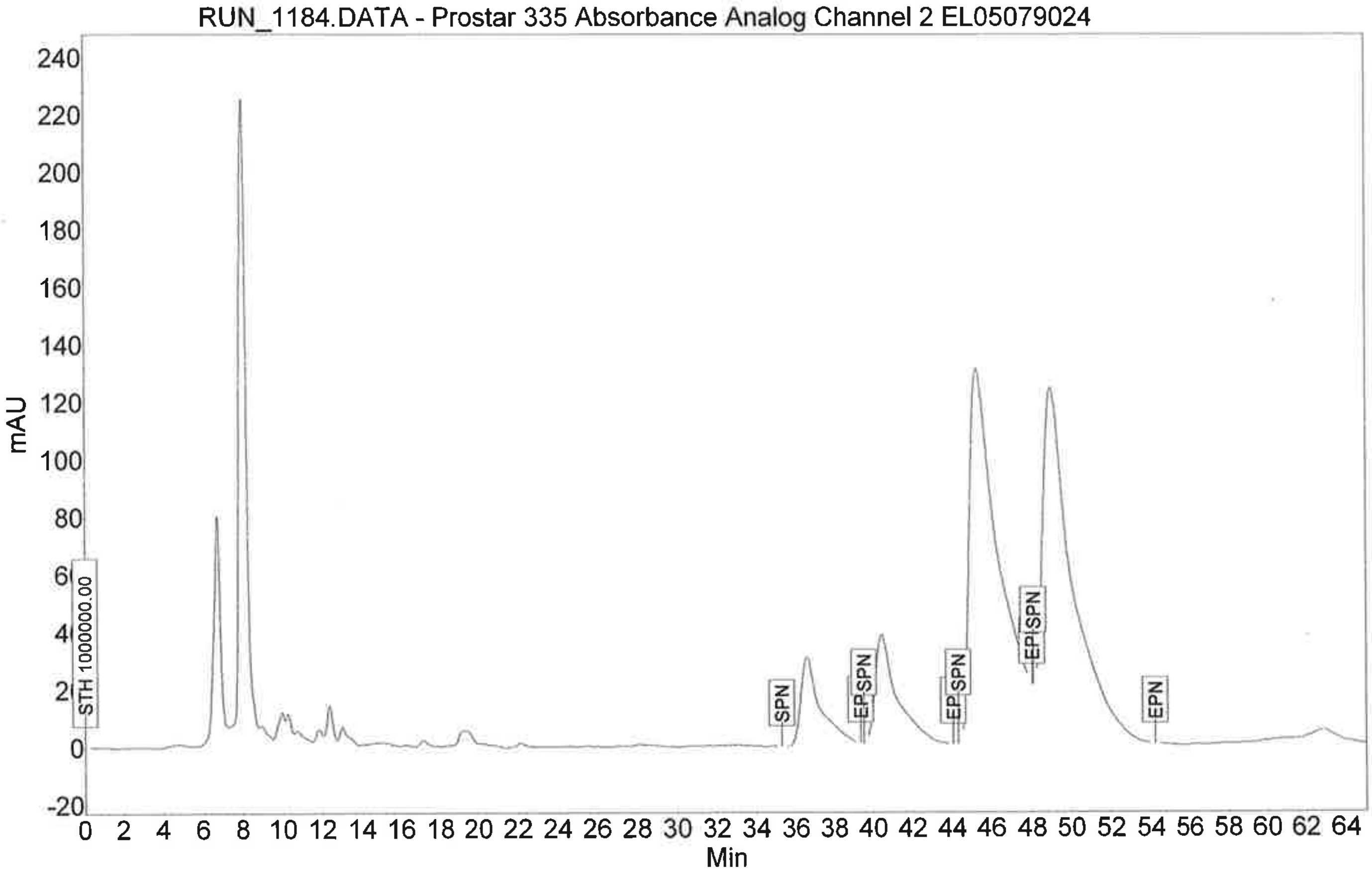

Index	Name	Time [Min]	Quantity [% Area]	Height [mAU]	Area [mAU.Min]	Area % [%]
1	UNKNOWN	36.56	8.30	30.7	36.0	8.301
2	UNKNOWN	40.47	11.28	37.9	48.9	11.282
3	UNKNOWN	45.25	43.01	125.4	186.3	43.008
4	UNKNOWN	48.99	37.41	106.8	162.1	37.409
Total			100.00	300.7	433.2	100.000

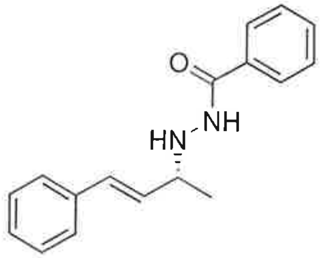

3r

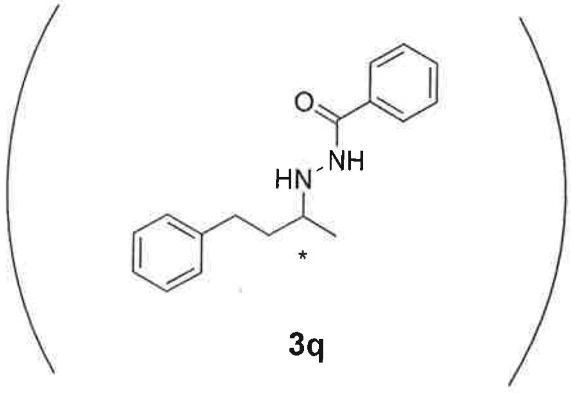

3q
